# Long-term safety and efficacy of upadacitinib compared with adalimumab in patients with rheumatoid arthritis: 7-year data from the SELECT-COMPARE study

**DOI:** 10.1136/rmdopen-2025-006657

**Published:** 2026-06-24

**Authors:** Roy Fleischmann, Jerzy Swierkot, Patrick Durez, Louis Bessette, Ricardo Blanco, Filip Van den Bosch, Duke Geem, Lingfeng Luo, Lauren D Smith, Diane Caballero, Sebastian Meerwein, Charles Peterfy, Yoshiya Tanaka, Eduardo Mysler

**Affiliations:** 1 University of Texas Southwestern Medical Center, Dallas, Texas, USA; 2 Metroplex Clinical Research Center, Dallas, Texas, USA; 3 Department of Rheumatology and Internal Medicine, Wroclaw Medical University, Wroclaw, Poland; 4 Institut de Recherche Expérimentale et Clinique, UCLouvain Saint-Luc, Pôle de Recherche en Rhumatologie, Brussels, Belgium; 5 Centre de recherche du CHU de Québec, Laval University, Quebec, Québec, Canada; 6 Rheumatology Division, Hospital University Marqués de Valdecilla, Immunopathology Group, IDIVAL, Santander, Spain; 7 Department of Internal Medicine and Pediatrics, VIB-UGent Center for Inflammation Research, Ghent University, Ghent, Belgium; 8 Immunology, AbbVie, North Chicago, Illinois, USA; 9 AbbVie Deutschland GmbH & Co KG, Ludwigshafen, Germany; 10 Spire Sciences Inc, Boca Raton, Florida, USA; 11 Department of Molecular Targeted Therapeutics, University of Occupational and Environmental Health, Kitakyushu, Japan; 12 Rheumatology, Organización Medica de Investigación, Buenos Aires, Argentina

**Keywords:** Antirheumatic Agents, Arthritis, Rheumatoid, Biological Therapy, Inflammation

## Abstract

**Objectives:**

To assess the safety and efficacy of upadacitinib versus adalimumab through 7 years in the ongoing SELECT-COMPARE study.

**Methods:**

Patients with rheumatoid arthritis (RA) and inadequate response to methotrexate (MTX) were randomised to placebo, upadacitinib 15 mg once daily or adalimumab 40 mg every other week (on background MTX). Inadequate responders were rescued to the alternate therapy, with placebo recipients switching to upadacitinib by week 26. Patients completing 48 weeks were eligible to enter the 10-year extension. Safety was assessed as treatment-emergent adverse events (TEAEs); efficacy was analysed by randomised group (non-responder imputation [NRI]) or treatment sequence (as observed [AO]).

**Results:**

Upadacitinib was generally well tolerated, displaying TEAE rates comparable to adalimumab, but numerically higher rates of herpes zoster, creatine phosphokinase elevation, non-melanoma skin cancer, lymphopenia and hepatic disorders. Responses with continuous upadacitinib or adalimumab were maintained through 372 weeks; week 372 Clinical Disease Activity Index remission (≤2.8) and 28-joint Disease Activity Score based on C-reactive protein<2.6 were achieved by 145/230 (63.0%) and 178/204 (83.2%) (AO)/142/651 (21.8%) and 172/651 (26.4%) (NRI) of patients with upadacitinib versus 46/86 (53.5%) and 59/81 (72.8%) (AO)/43/327 (13.1%) and 55/327 (16.8%) (NRI) with adalimumab. Initial non-responders/incomplete responders benefited from switching, with improvements in efficacy endpoints maintained through week 336 post switch, without additional safety concerns.

**Conclusion:**

The safety profile of upadacitinib remained consistent with previous analyses, with no new safety concerns through 7 years. Upadacitinib and adalimumab (continuous or rescue treatment) maintained disease activity targets throughout the 7-year treatment period. Upadacitinib exhibited an acceptable benefit–risk profile for long-term RA treatment.

**Trial registration number:**

NCT02629159.

**WHAT IS ALREADY KNOWN ON THIS TOPIC**

- Upadacitinib has demonstrated efficacy with an acceptable safety profile in the SELECT phase III clinical trial programme of patients with rheumatoid arthritis (RA), including in the SELECT-COMPARE long-term extension through 5 years.

**WHAT THIS STUDY ADDS**

- This open-label extension study of upadacitinib in the SELECT-COMPARE RA programme provides long-term safety and efficacy data compared with adalimumab through 7 years. There was no evidence of increased risk of adverse events with longer exposure to upadacitinib, while favourable efficacy of upadacitinib compared with adalimumab was maintained.

**HOW THIS STUDY MIGHT AFFECT RESEARCH, PRACTICE OR POLICY**

- The results further support the acceptable benefit–risk profile for upadacitinib in the long-term management of RA.

## Introduction

 Rheumatoid arthritis (RA) is a chronic, systemic, progressive inflammatory disease primarily affecting the joints and causing cartilage and bone damage. These symptoms may lead to disability and decreased quality of life if not treated effectively.^1^,^4^ Despite advances in therapy, there remains an unmet need for treatments that offer long-term symptom control with a reasonable safety profile and improve patients’ quality of life.^5^,^7^ Current guidelines from the American College of Rheumatology (ACR)^8^ and European Alliance of Associations for Rheumatology (EULAR)^9^ recommend conventional synthetic disease-modifying anti-rheumatic drugs (csDMARDs), preferably methotrexate (MTX), as first-line therapy, with biologic DMARDs (bDMARDs) used as second-line therapy due to their established effectiveness and safety profile. Patients with insufficient response to bDMARDs,^10 11^ including tumour necrosis factor (TNF) inhibitors,^12^,^14^ may benefit from switching to Janus kinase (JAK) inhibitors, a class of small-molecule drugs administered orally and classified as targeted synthetic DMARDs. JAK inhibitors are established as an alternative therapy for RA,^1215^,^19^ provided that patients are adequately screened and monitored for risk factors such as infection, cardiovascular disorders, thrombosis and malignancies.^5 12^

The safety and efficacy of the JAK inhibitor upadacitinib in RA have been extensively evaluated in the Study of Efficacy and Long-term Evaluation of Corticosteroid-free Therapy (SELECT) clinical trial programme.^1416 20^,^24^ In the SELECT-COMPARE phase III study, upadacitinib has been actively compared with the TNF inhibitor adalimumab, each in combination with background MTX, in patients with moderately to severely active RA and inadequate response to MTX.^14^ The clinical responses previously observed with upadacitinib at 3 years^3^ were maintained through 5 years^25^ and were generally numerically higher compared with the responses observed with adalimumab. The safety profile has remained consistent throughout the trial, with similar adverse event (AE) rates between the two treatment groups.^3 25^ In addition, a subanalysis of the SELECT-COMPARE study showed clinical improvements through 5 years with no new safety concerns in patients who had insufficient response to their initial randomised treatment and were switched to the alternate therapy.^13 26 27^

Long-term treatment safety and efficacy are paramount, as lifelong treatment is usually required to control disease activity in RA.^28^ Here, we present the 7-year findings on the safety and efficacy of upadacitinib versus adalimumab, each in combination with background MTX, from the ongoing open-label long-term extension (LTE) of the SELECT-COMPARE study.

## Methods

### Study design and patients

The study design and eligibility criteria of the ongoing SELECT-COMPARE trial (NCT02629159) have been previously published.^14 27^ Briefly, eligible patients were ≥18 years old with a diagnosis of RA (meeting the 2010 ACR/EULAR classification criteria)^29^ and inadequate response to MTX (received for ≥3 months at a stable dose of 15–25 mg/week, or ≥10 mg/week in patients intolerant to MTX at ≥12.5 mg/week, for ≥4 weeks prior to the first dose of study drug). Patients with prior exposure to a JAK inhibitor or adalimumab, or inadequate response to a previous bDMARD, were excluded.

The initial 48-week double-blind phase (placebo-controlled for the first 26 weeks) was followed by an active comparator-controlled, open-label phase continuing into an ongoing 10-year LTE phase. Eligible patients were randomised 2:2:1 to receive either upadacitinib 15 mg once daily (QD), adalimumab 40 mg every other week (EOW) or placebo, while on background MTX. During the initial 26 weeks, a rescue protocol, where patients were immediately switched from upadacitinib to adalimumab or from adalimumab to upadacitinib without a washout period, was implemented for non-responders (NR; patients who failed to achieve ≥20% improvement from baseline in tender joint count based on 68 joints [TJC68] and swollen joint count based on 66 joints [SJC66]; switched at weeks 14, 18 or 22). Additionally, incomplete responders (IR; patients who did not achieve low disease activity [LDA] defined as Clinical Disease Activity Index [CDAI] ≤10) were rescued at week 26. All patients on placebo who were not previously rescued were switched to upadacitinib at week 26. Only one treatment switch was allowed per patient, and no switching was permitted after week 26.

Starting at week 48, initiation or modification of concomitant csDMARDs (restricted to oral or parenteral MTX, sulfasalazine, hydroxychloroquine, chloroquine and leflunomide) was allowed, but limited to concomitant use of ≤2 csDMARDs, except for the combination of MTX and leflunomide. Following completion of the 48-week double-blind period, patients could enter the ongoing LTE (which became open-label after the last patient completed their week 48 visit) and continue to receive upadacitinib 15 mg QD or adalimumab 40 mg EOW.

### Safety

Safety was evaluated by calculating exposure-adjusted event rates (EAERs) and exposure-adjusted incidence rates (EAIRs) of treatment-emergent adverse events (TEAEs) per 100 patient-years (PY) through 7 years up to the cut-off date (19 September 2024), for all patients receiving ≥1 dose of upadacitinib 15 mg or adalimumab 40 mg. Overall TEAEs and TEAEs of special interest (including, but not limited to, serious infections, opportunistic infections, herpes zoster, malignancies, major adverse cardiovascular events [MACE] and venous thromboembolic events [VTE]), and the proportions of patients experiencing potentially clinically significant (grade 3 or 4) abnormalities in laboratory parameters, were evaluated through week 372 in all patients who received ≥1 dose of upadacitinib or adalimumab.

An independent cardiovascular adjudication committee adjudicated deaths, MACE and VTE in a blinded manner. All TEAEs were categorised and graded based on predefined criteria, such as the Medical Dictionary for Regulatory Activities V.27.0, Rheumatology Common Toxicity Criteria V.2.0 and the National Cancer Institute Common Toxicity Criteria. The severity of AEs was assessed by the investigators and as per the study protocol. TEAEs were classified as transient if an end date was available for the event.

### Efficacy endpoints

Efficacy endpoints evaluated through week 372 included the proportions of patients achieving CDAI LDA (≤10) or remission (≤2.8),^30^ 28-joint Disease Activity Score based on C-reactive protein (DAS28[CRP]) ≤3.2 or <2.6^31 32^ and ≥20/50/70% improvement in ACR criteria (ACR20/50/70 responses).^33^ Changes from baseline in ACR components, including TJC68, SJC66, patient’s global assessment of disease activity (PtGA), physician’s global assessment of disease activity (PhGA), patient’s assessment of pain (PtPain; 0–100 mm scale), Health Assessment Questionnaire-Disability Index (HAQ-DI; 0–3 scale) and high-sensitivity CRP (hsCRP; mg/L) were also evaluated.

Cumulative steroid usage (any systemic use regardless of indication, excluding topical and intradermal) was calculated in patients who entered the LTE on study drug. Cumulative steroid usage was calculated at each analysis visit by summing up prednisone daily equivalent steroid from baseline to the target date of each analysis visit up to week 372. For each steroid record, prednisone daily equivalent dose was calculated using the formula below:


$$\frac{Oral\;steroid\;dose}{Equivalent\;dose\;}\times 5\;mg\times Frequency$$

Details on the equivalent steroid dose and equivalent dose frequency are provided in the online supplemental methods.

### Statistical analysis

Safety data were assessed through 7 years in all patients receiving ≥1 dose of upadacitinib 15 mg or adalimumab 40 mg. TEAEs were assigned based on the time of event occurrence to either ‘any upadacitinib’ (patients on continuous upadacitinib and those rescued to upadacitinib from placebo or adalimumab) or ‘any adalimumab’ (patients on continuous adalimumab and those rescued to adalimumab from upadacitinib). Key safety endpoints, such as MACE, VTE and malignancies excluding non-melanoma skin cancer (NMSC), were also analysed in the continuous upadacitinib and adalimumab groups.

Efficacy data for CDAI, DAS28(CRP) and ACR20/50/70 responses were evaluated through week 372 for all patients receiving ≥1 dose of upadacitinib or adalimumab, based on the originally randomised and treatment sequence groups (continuous upadacitinib, placebo to upadacitinib, adalimumab to upadacitinib, continuous adalimumab and upadacitinib to adalimumab).

For binary endpoints and continuous endpoints analysed by treatment sequence, as observed (AO) analyses were conducted based on patients continuing in the LTE, without imputation for missing data. Binary endpoints in the randomised treatment groups were also evaluated using non-responder imputation (NRI) to account for rescue, study drug discontinuation and missing data. All p values are nominal.

## Results

### Patient disposition and demographics

**Figure 1:**
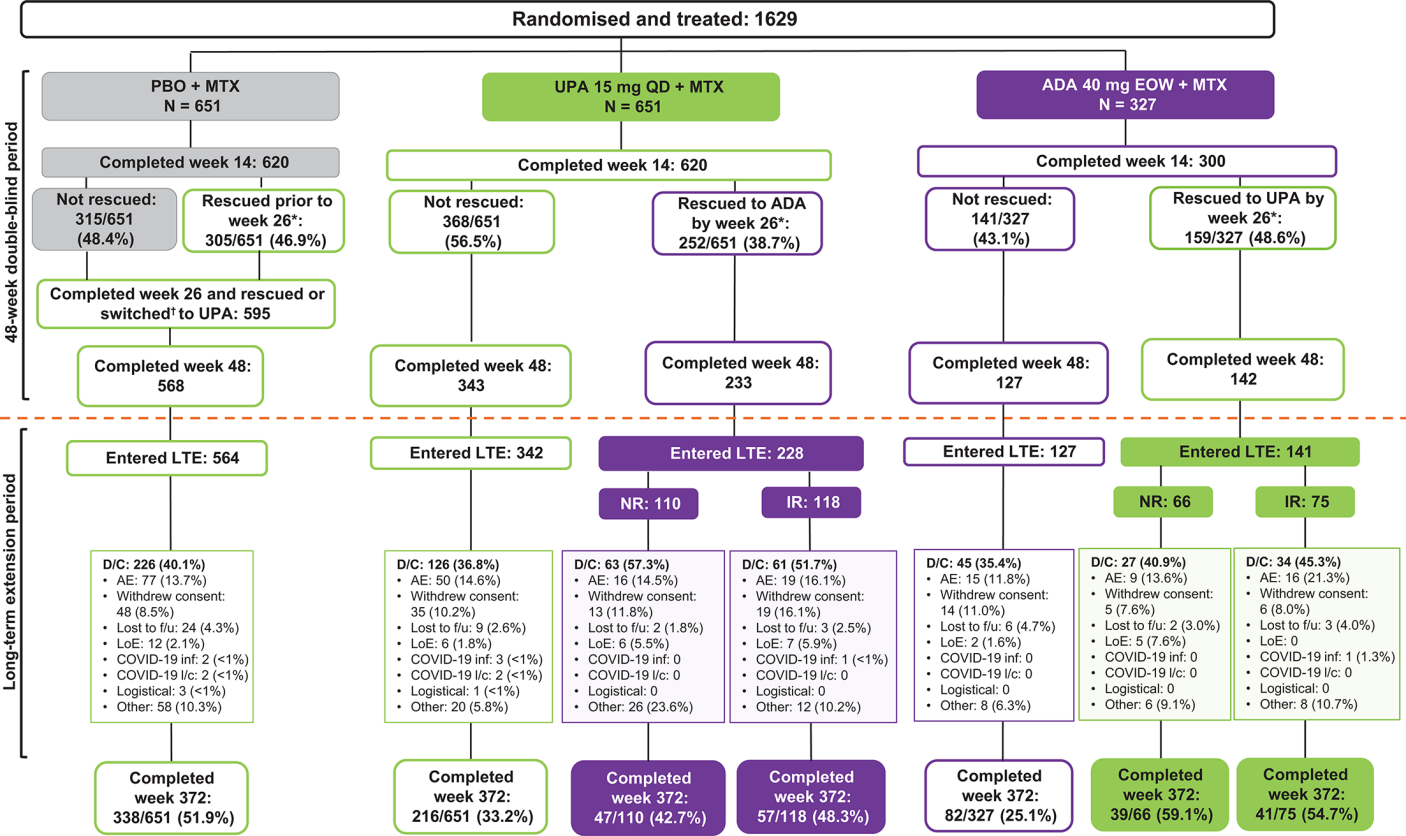
Patient disposition. Numbers of patients on study drug are shown, with primary reason for discontinuation summarised during the long-term extension through week 372. Patients who discontinued the study drug are counted under each reason given for discontinuation; therefore, the sum of the counts given for the reasons may be greater than the overall number of discontinuations. *Rescue occurred only before or at week 26; no rescue was allowed after week 26. †All patients on PBO not previously rescued (at weeks 14, 18 or 22) were switched to UPA at week 26. ADA, adalimumab; AE, adverse event; D/C, discontinued; EOW, every other week; f/u, follow-up; inf, infection; l/c, logistical restrictions; IR, incomplete responders; LoE, lack of efficacy; LTE, long-term extension; MTX, methotrexate; NR, non-responders; PBO, placebo; QD, once daily; UPA, upadacitinib; wk, week.

A total of 1629 patients were initially randomised to placebo (n=651), upadacitinib (n=651) or adalimumab (n=327) (figure 1). Of initially randomised patients, 342/651 entered the LTE on continuous upadacitinib and 127/327 on continuous adalimumab treatment. A numerically greater proportion of patients completed the 7-year study period on continuous upadacitinib (216/651; 33.2%) compared with those on continuous adalimumab (82/327; 25.1%). At the 7-year cut-off, 820 patients remained on upadacitinib or adalimumab across treatment groups: placebo to upadacitinib, n=338; continuous upadacitinib, n=216; continuous adalimumab, n=82; adalimumab to upadacitinib, n=80 (NR: n=39; IR: n=41); and upadacitinib to adalimumab, n=104 (NR: n=47; IR: n=57).

The demographics and baseline characteristics of patients have been previously reported^14^ and were generally well balanced across treatment groups, including between patients who switched treatment^26^ and the overall study population.^14^ Briefly, 79% of the study population were female, with a mean age (±SD) of 54 (±12) years, a mean disease duration of 8 (±8) years and a CDAI Score (scale, 0–76) of 40 (±13) at baseline. Among patients receiving glucocorticoids at baseline, 37.3% (57/153) of those on continuous upadacitinib and 40.4% (23/57) of those on continuous adalimumab had discontinued glucocorticoids by week 372. Comparable proportions were observed among rescued patients (placebo to upadacitinib: 28.5% [63/221]; upadacitinib to adalimumab: 29.2% [21/72]; and adalimumab to upadacitinib: 29.7% [19/64]).

At baseline, similar proportions of patients in the placebo, upadacitinib and adalimumab groups (73.3% [478/652], 77.7% [505/650] and 73.7% [241/327], respectively; safety analysis set) had ≥1 cardiovascular risk factor (including history of hypertension, diabetes mellitus, dyslipidaemia and tobacco/nicotine use); 14% (91/652) of patients had ≥3 cardiovascular risk factors at baseline (safety analysis set; online supplemental table S1).

### Safety

#### All study drug exposure

A total of 1417 patients received any upadacitinib, and 579 patients received any adalimumab over the 7-year study period. The cumulative exposures for upadacitinib and adalimumab were 6228.0 and 2004.3 PY, respectively (table 1). The rates of overall TEAEs, serious TEAEs and TEAEs leading to discontinuation of the study drug were similar between the two groups. The most common TEAEs (>5 E/100 PY) reported in both groups were urinary tract infection, upper respiratory tract infection and nasopharyngitis (online supplemental table S2).

**Table 1:** Exposure-adjusted event rates of treatment-emergent adverse events through 372 weeks

EAER, E (E/100 PY) (95% CI)	Any upadacitinib 15 mg QD (n=1417) PY=6228.0	Any adalimumab 40 mg EOW (n=579) PY=2004.3
Any TEAE	10902 (175.0) (171.8 to 178.4)	3628 (181.0) (175.2 to 187.0)
Serious TEAE	693 (11.1) (10.0 to 12.0)	257 (12.8) (11.3 to 14.5)
TEAE leading to study drug D/C	248 (4.0) (3.5 to 4.5)	99 (4.9) (4.0 to 6.0)
Any COVID-19-related AE	324 (5.2) (4.7 to 5.8)	104 (5.2) (4.2 to 6.3)
All deaths*	47 (0.8) (0.6 to 1.0)	17 (0.8) (0.5 to 1.4)
Serious infection	208 (3.3) (2.9 to 3.8)	64 (3.2) (2.5 to 4.1)
Opportunistic infection†	13 (0.2) (0.1 to 0.4)	3 (0.1) (0.0 to 0.4)
Herpes zoster	161 (2.6) (2.2 to 3.0)	19 (0.9) (0.6 to 1.5)
Active tuberculosis	3 (<0.1) (0.0 to 0.1)	4 (0.2) (0.1 to 0.5)
Malignancy (excluding NMSC)	38 (0.6) (0.4 to 0.8)	18 (0.9) (0.5 to 1.4)
NMSC	34 (0.5) (0.4 to 0.8)	3 (0.1) (0.0 to 0.4)
Lymphoma	0	3 (0.1) (0.0 to 0.4)
Adjudicated MACE‡	19 (0.3) (0.2 to 0.5)	8 (0.4) (0.2 to 0.8)
Adjudicated VTE§	19 (0.3) (0.2 to 0.5)	7 (0.3) (0.1 to 0.7)
Adjudicated GI perforation	2 (<0.1) (0.0 to 0.1)	0
Renal dysfunction	14 (0.2) (0.1 to 0.4)	6 (0.3) (0.1 to 0.7)
Anaemia	158 (2.5) (2.2 to 3.0)	61 (3.0) (2.3 to 3.9)
Lymphopenia	166 (2.7) (2.3 to 3.1)	17 (0.8) (0.5 to 1.4)
Neutropenia	130 (2.1) (1.7 to 2.5)	42 (2.1) (1.5 to 2.8)
CPK elevation	221 (3.5) (3.1 to 4.0)	33 (1.6) (1.1 to 2.3)
Hepatic disorder	533 (8.6) (7.8 to 9.3)	106 (5.3) (4.3 to 6.4)

Safety was assessed up to week 372, through the cut-off date of 19 September 2024. TEAEs included any AE with an onset date on or after the first dose of study drug and up to 30 days after the last dose of placebo or upadacitinib and 70 days for adalimumab, if patients discontinued prematurely. Data are for all patients receiving upadacitinib or adalimumab, including exposure to rescue treatment after switching, with assignment based on drug exposure at the time of event.

* Includes treatment-emergent (occurring ≤30 days after the last dose of upadacitinib or ≤70 days after the last dose of adalimumab).

† Excluding tuberculosis and herpes zoster.

‡ Defined as cardiovascular death (includes acute myocardial infarction, sudden cardiac death, heart failure, cardiovascular procedure-related death, death due to cardiovascular haemorrhage, fatal stroke, pulmonary embolism and other cardiovascular causes), non-fatal myocardial infarction and non-fatal stroke.

§ Includes deep vein thrombosis and pulmonary embolism (fatal and non-fatal).

AE, adverse event; CI, confidence interval; CPK, creatine phosphokinase; D/C, discontinued; E, events; EAER, exposure-adjusted event rate; EOW, every other week; GI, gastrointestinal; MACE, major adverse cardiovascular event; NMSC, non-melanoma skin cancer; PY, patient-years; QD, once daily; TEAE, treatment-emergent adverse event; VTE, venous thromboembolic events.

Among AEs of special interest, MACE, VTE, malignancy excluding NMSC and neutropenia occurred at similar rates in both treatment groups. Numerically higher rates of herpes zoster (2.6 vs 0.9 events [E]/100 PY), creatine phosphokinase (CPK) elevation (3.5 vs 1.6 E/100 PY), NMSC (0.5 vs 0.1 E/100 PY), lymphopenia (2.7 vs 0.8 E/100 PY) and hepatic disorder (8.6 vs 5.3 E/100 PY) were observed with upadacitinib compared with adalimumab (table 1). Two events of adjudicated gastrointestinal perforation (<0.1 E/100 PY) were reported (one in the continuous upadacitinib group [day 2583] and one in the adalimumab to upadacitinib group [day 168 after switching]; table 2). Most cases of herpes zoster infection were non-serious and non-disseminated, involving a single dermatome. One patient receiving upadacitinib exhibited central nervous system involvement (Ramsay Hunt syndrome), and ophthalmic involvement was observed in 11 cases, with two occurring in the adalimumab group and nine in the upadacitinib group. A total of 12 events (0.2 E/100 PY) of serious herpes zoster occurred in the any upadacitinib group. None of the serious events of herpes zoster occurred in vaccinated patients.

**Table 2:** Exposure-adjusted event rates of treatment-emergent adverse events through 372 weeks in patients who received continuous upadacitinib and adalimumab and those who switched to the alternate treatment by week 26

EAER, E (E/100 PY) (95% CI)	Continuous upadacitinib 15 mg QD (n=398) PY=2161.8	Adalimumab switched to upadacitinib (n=159) PY=794.7	Continuous adalimumab 40 mg EOW (n=168) PY=810.3	Upadacitinib switched to adalimumab (n=252) PY=1130.6
Any TEAE	3918 (181.2) (175.6 to 187.0)	1256 (158.0) (149.4 to 167.0)	1309 (161.5) (152.9 to 170.5)	2112 (186.8) (178.9 to 194.9)
Serious TEAE	224 (10.4) (9.0 to 11.8)	124 (15.6) (13.0 to 18.6)	112 (13.8) (11.4 to 16.6)	142 (12.6) (10.6 to 14.8)
TEAE leading to study drug D/C	99 (4.6) (3.7 to 5.6)	38 (4.8) (3.4 to 6.6)	51 (6.3) (4.7 to 8.3)	48 (4.2) (3.1 to 5.6)
Any COVID-19-related AE	139 (6.4) (5.4 to 7.6)	43 (5.4) (3.9 to 7.3)	48 (5.9) (4.4 to 7.9)	56 (5.0) (3.7 to 6.4)
All deaths*	15 (0.7) (0.4 to 1.1)	8 (1.0) (0.4 to 2.0)	9 (1.1) (0.5 to 2.1)	8 (0.7) (0.3 to 1.4)
Serious infection	80 (3.7) (2.9 to 4.6)	36 (4.5) (3.2 to 6.3)	35 (4.3) (3.0 to 6.0)	28 (2.5) (1.6 to 3.6)
Opportunistic infection†	5 (0.2) (0.1 to 0.5)	1 (0.1) (0.0 to 0.7)	2 (0.2) (0.0 to 0.9)	1 (<0.1) (0.0 to 0.5)
Herpes zoster	50 (2.3) (1.7 to 3.0)	30 (3.8) (2.5 to 5.4)	7 (0.9) (0.3 to 1.8)	12 (1.1) (0.5 to 1.9)
Active tuberculosis	1 (<0.1) (0.0 to 0.3)	0	1 (0.1) (0.0 to 0.7)	3 (0.3) (0.1 to 0.8)
Malignancy (excluding NMSC)	14 (0.6) (0.4 to 1.1)	6 (0.8) (0.3 to 1.6)	6 (0.7) (0.3 to 1.6)	12 (1.1) (0.5 to 1.9)
NMSC	14 (0.6) (0.4 to 1.1)	2 (0.3) (0.0 to 0.9)	1 (0.1) (0.0 to 0.7)	2 (0.2) (0.0 to 0.6)
Lymphoma	0	0	2 (0.2) (0.0 to 0.9)	1 (<0.1) (0.0 to 0.5)
Adjudicated MACE‡	2 (<0.1) (0.0 to 0.3)	3 (0.4) (0.1 to 1.1)	4 (0.5) (0.1 to 1.3)	4 (0.4) (0.1 to 0.9)
Adjudicated VTE§	5 (0.2) (0.1 to 0.5)	6 (0.8) (0.3 to 1.6)	3 (0.4) (0.1 to 1.1)	4 (0.4) (0.1 to 0.9)
Adjudicated GI perforation	1 (<0.1) (0.0 to 0.3)	1 (0.1) (0.0 to 0.7)	0	0
Renal dysfunction	5 (0.2) (0.1 to 0.5)	3 (0.4) (0.1 to 1.1)	2 (0.2) (0.0 to 0.9)	4 (0.4) (0.1 to 0.9)
Anaemia	47 (2.2) (1.6 to 2.9)	25 (3.1) (2.0 to 4.6)	18 (2.2) (1.3 to 3.5)	37 (3.3) (2.3 to 4.5)
Lymphopenia	61 (2.8) (2.2 to 3.6)	14 (1.8) (1.0 to 3.0)	2 (0.2) (0.0 to 0.9)	9 (0.8) (0.4 to 1.5)
Neutropenia	53 (2.5) (1.8 to 3.2)	14 (1.8) (1.0 to 3.0)	7 (0.9) (0.3 to 1.8)	33 (2.9) (2.0 to 4.1)
CPK elevation	79 (3.7) (2.9 to 4.6)	19 (2.4) (1.4 to 3.7)	12 (1.5) (0.8 to 2.6)	21 (1.9) (1.1 to 2.8)
Hepatic disorder	185 (8.6) (7.4 to 9.9)	37 (4.7) (3.3 to 6.4)	39 (4.8) (3.4 to 6.6)	59 (5.2) (4.0 to 6.7)

Safety was assessed up to week 372, through the cut-off date of 19 September 2024. TEAEs included any AE with an onset date on or after the first dose of study drug and up to 30 days after the last dose of placebo or upadacitinib and 70 days for adalimumab, if patients discontinued prematurely. Data are for all patients receiving upadacitinib or adalimumab, including exposure to rescue treatment after switching, with assignment based on drug exposure at the time of event.

* Includes treatment-emergent (occurring ≤30 days after the last dose of upadacitinib or ≤70 days after the last dose of adalimumab).

† Excluding tuberculosis and herpes zoster.

‡ Defined as cardiovascular death (includes acute myocardial infarction, sudden cardiac death, heart failure, cardiovascular procedure-related death, death due to cardiovascular haemorrhage, fatal stroke, pulmonary embolism and other cardiovascular causes), non-fatal myocardial infarction and non-fatal stroke.

§ Includes deep vein thrombosis and pulmonary embolism (fatal and non-fatal).

AE, adverse event; CI, confidence interval; CPK, creatine phosphokinase; D/C, discontinuation; E, event; EAER, exposure-adjusted event rate; EOW, every other week; GI, gastrointestinal; MACE, major adverse cardiovascular events; NMSC, non-melanoma skin cancer; PY, patient-years; QD, once daily; TEAE, treatment-emergent adverse event; VTE, venous thromboembolic events.

The rates for malignancies excluding NMSC were 0.6 E/100 PY for upadacitinib and 0.9 E/100 PY for adalimumab. The rates of NMSC were numerically higher for upadacitinib (0.5 E/100 PY) than for adalimumab (0.1 E/100 PY). Details on malignancy events, adjudicated MACE and adjudicated VTE are provided in online supplemental table S3. There was no notable pattern in the types of malignancies that were observed, except for higher EAER for NMSC with upadacitinib (0.5 E/100 PY vs <0.1 E/100 PY with adalimumab). In total, there were 21 basal cell carcinomas in 17 patients (18 events in 16 patients who received at least one dose of upadacitinib; 5 events in 3 patients who received at least one dose of adalimumab); and 22 squamous cell carcinomas in 13 patients who received at least one dose of upadacitinib. All cases were treated by surgical resection; information on adjuvant treatments after resection is not available.

The rate of CPK elevations was 3.5 E/100 PY with upadacitinib compared with 1.6 E/100 PY with adalimumab, with most cases being mild to moderate in severity, without necessitating study drug discontinuation. The presence of myopathy associated with elevated CPK levels was not verified. There were two cases of rhabdomyolysis, one in the any upadacitinib group and one in the any adalimumab group (<0.1 E/100 PY). Most cases of hepatic disorder involved mild-to-moderate alanine aminotransferase (ALT) or aspartate aminotransferase (AST) elevations. There were 21 hepatic disorder events that led to permanent study drug discontinuation in the any upadacitinib group and 7 events in the any adalimumab group (0.3 E/100 PY for both). Transaminase elevations accounted for the majority of hepatic disorders leading to discontinuation. The proportions of patients experiencing potentially clinically significant (grade 3 or 4) abnormalities in laboratory parameters were evaluated through week 372 in all patients who received ≥1 dose of upadacitinib or adalimumab and are summarised in online supplemental table S4.

A total of 64 deaths (59 of which were treatment emergent) occurred in patients receiving any upadacitinib (47 deaths) and any adalimumab (17 deaths) (0.8 E/100 PY for both groups). Among TEAEs leading to death, the most common in the any upadacitinib group were cardiac events (n=14, 0.2 E/100 PY) and infections and infestations (n=17, 0.3 E/100 PY), including six events of COVID-19 pneumonia and five events of COVID-19 (both EAERs <0.1). In the any adalimumab group, the most common TEAEs leading to death were infections and infestations (n=4, 0.2 E/100 PY) and malignancy (n=3, 0.1 E/100 PY). The TEAEs leading to death are summarised in online supplemental table S5.

EAIRs of TEAEs through 372 weeks were similar between the two groups, with the exception of herpes zoster (2.3 vs 0.8 n/100 PY), CPK elevation (2.3 vs 1.2 n/100 PY), hepatic disorder (4.6 vs 2.9 n/100 PY) and lymphopenia (1.5 vs 0.5 n/100 PY), which were higher in the any upadacitinib group (online supplemental table S6).

#### Long-term study drug exposure in patients who switched treatment

Among patients who switched to the alternate treatment by week 26, the overall exposure-adjusted TEAE rate at week 372 was lower among patients who switched to upadacitinib compared with those who switched to adalimumab (158.0 vs 186.8 E/100 PY, respectively). The rate of serious infection was 4.5 E/100 PY among patients who switched to upadacitinib and 2.5 E/100 PY among those who switched to adalimumab, and the rate of herpes zoster infection was 3.8 E/100 PY among patients who switched to upadacitinib and 1.1 E/100 PY among those who switched to adalimumab (table 2). Among AEs of special interest, the most frequent in both groups was hepatic disorder (4.7 and 5.2 E/100 PY in patients who switched to upadacitinib and those who switched to adalimumab, respectively). The rates of CPK elevation, lymphopenia and VTE were higher among patients who switched to upadacitinib (2.4, 1.8 and 0.8 E/100 PY, respectively), compared with those who switched to adalimumab (1.9, 0.8 and 0.4 E/100 PY, respectively), while neutropenia was more frequent in the group that switched to adalimumab (2.9 vs 1.8 E/100 PY, respectively). The EAERs for most other TEAEs were largely similar between the two switch groups (table 2).

### Efficacy

#### All treatment sequences

**Figure 2:**
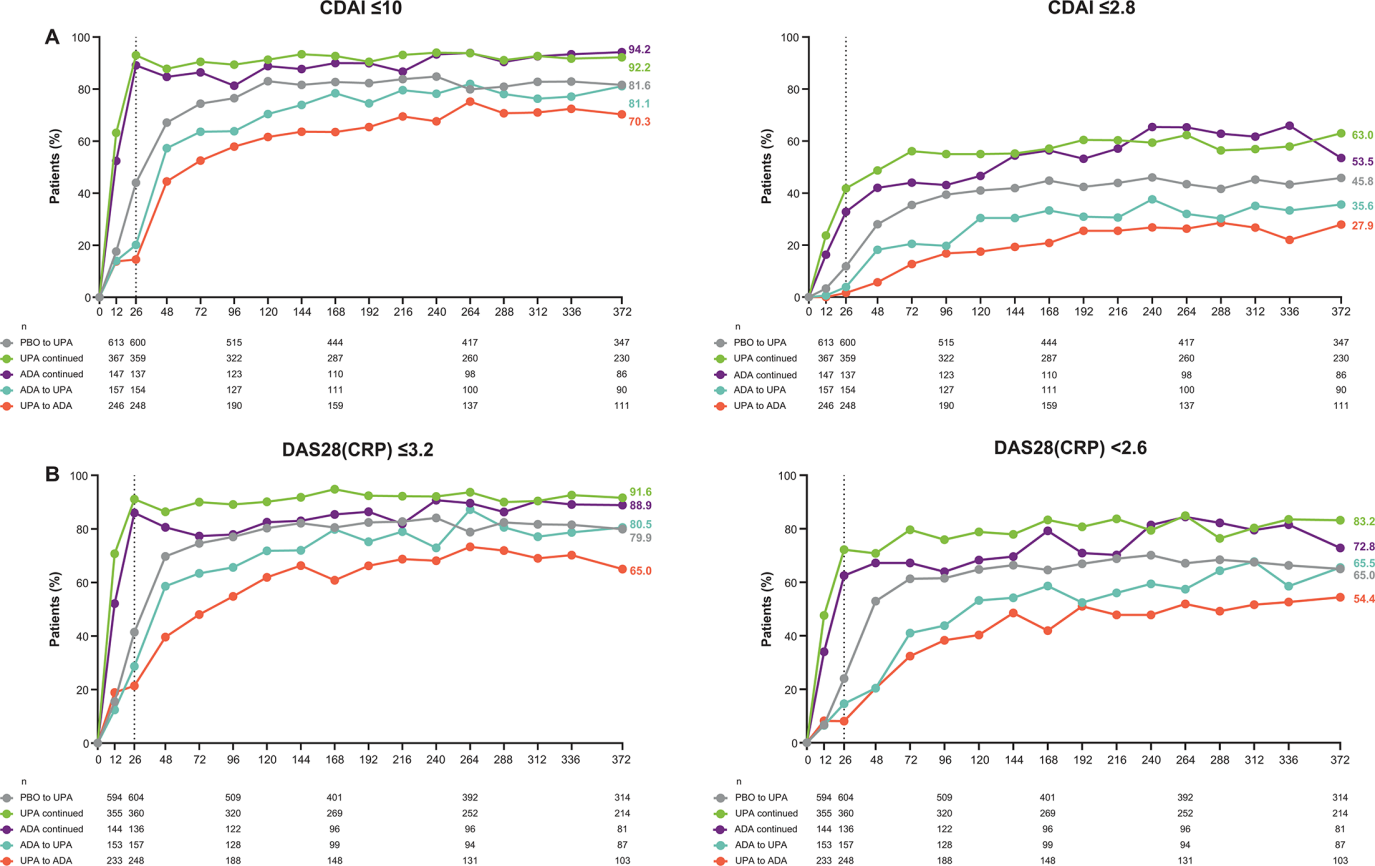
Proportions of patients achieving (A) CDAI LDA (≤10) and CDAI remission (≤2.8) and (B) DAS28(CRP) ≤3.2 and DAS28(CRP) ≤2.6 through 372 weeks (AO). Groups are shown by treatment sequence AO, without imputation for missing data. All patients in the PBO group who were not previously rescued were switched to UPA at week 26. Sample sizes (n) are shown below each graph. Data points plotted here are shown in online supplemental table S7. ADA, adalimumab; AO, as observed; CDAI, Clinical Disease Activity Index; DAS28(CRP), 28-joint Disease Activity Score based on C-reactive protein; LDA, low disease activity; PBO, placebo; UPA, upadacitinib.

The proportions of patients who achieved disease activity targets were consistent across different treatment sequence groups over the 7-year LTE period, with higher proportions of patients on continuous upadacitinib and continuous adalimumab achieving treatment targets over time compared with patients who received rescue treatment (AO data; figure 2 and online supplemental table S7). Similar proportions of patients receiving continuous upadacitinib and continuous adalimumab achieved CDAI LDA and remission throughout the 7-year LTE period, with numerical differences mostly in favour of upadacitinib (AO data; figure 2A). A similar trend was observed for DAS28(CRP) over time, with numerical differences in favour of upadacitinib throughout the 7-year LTE (AO data; figure 2B).

On NRI analysis, patients initially randomised to upadacitinib showed consistently greater efficacy across timepoints compared with those initially randomised to adalimumab. At week 372, CDAI LDA was achieved by 31.2% (203/651) vs 23.5% (77/327); CDAI remission by 21.8% (142/651) vs 13.1% (43/327); DAS28(CRP) ≤3.2 by 28.7% (187/651) vs 20.8% (68/327); and DAS28(CRP) <2.6 by 26.4% (172/651) vs 16.8% (55/327) of patients initially randomised to upadacitinib and to adalimumab, respectively (NRI data; online supplemental figure S1 and online supplemental table S11).

**Figure 3:**
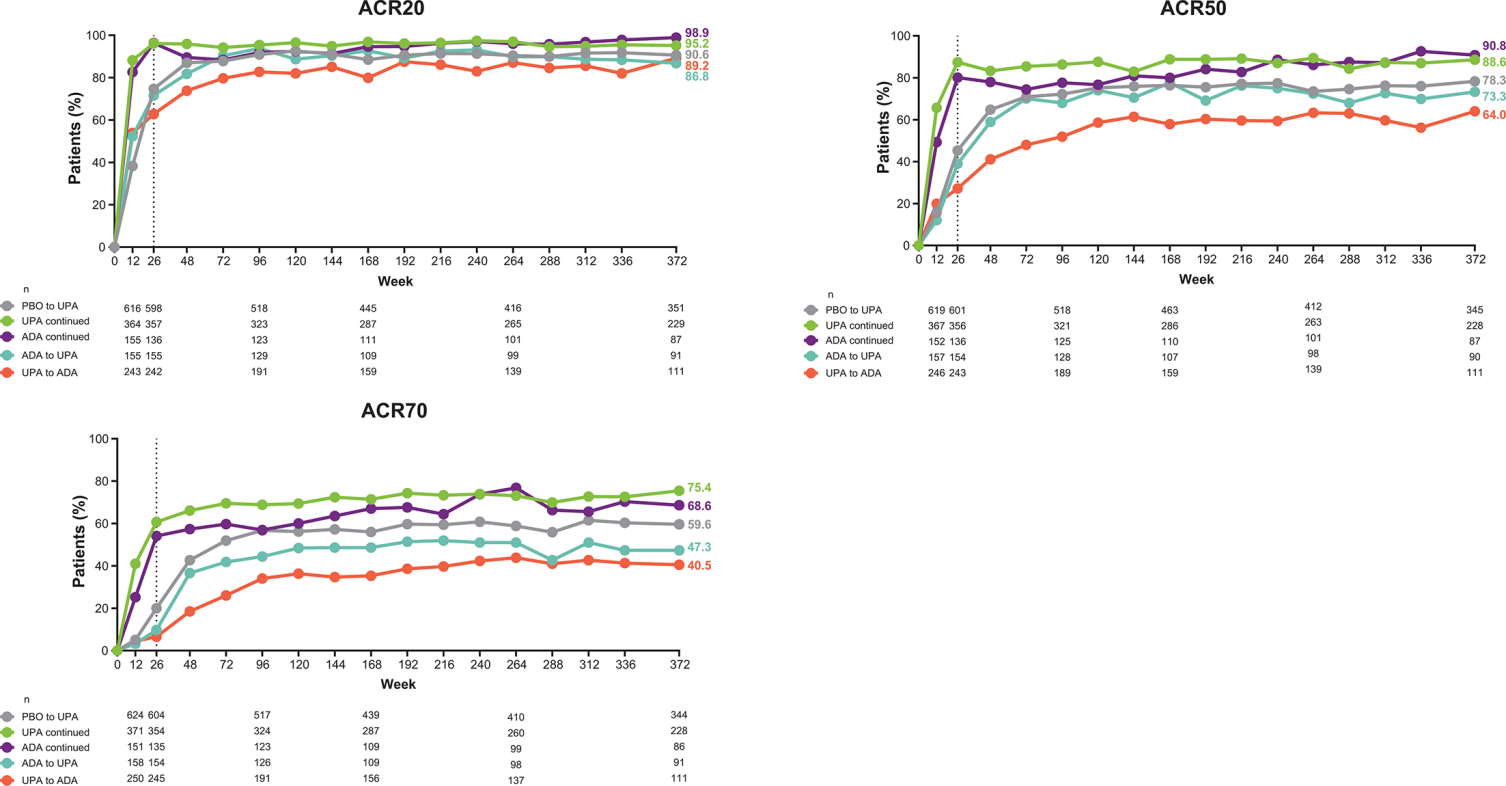
Proportions of patients achieving ACR20, ACR50 and ACR70 through 372 weeks (AO). Groups are shown by treatment sequence AO, without imputation for missing data. All patients in the placebo group who were not previously rescued were switched to UPA at week 26. Sample sizes (n) are shown below each graph. Data points plotted here are shown in online supplemental table S8. ACR20/50/70, ≥20/50/70% improvement in American College of Rheumatology response criteria; ADA, adalimumab; AO, as observed; PBO, placebo; UPA, upadacitinib.

Higher proportions of patients on continuous upadacitinib and continuous adalimumab achieved ACR response rates over time compared with patients who received rescue treatment. The AO treatment analysis showed that ACR20 and ACR50 response rates were generally similar for patients receiving continuous upadacitinib and continuous adalimumab over the 7-year LTE period, with 95.2% (218/229) vs 98.9% (86/87) achieving ACR20 and 88.6% (202/228) vs 90.8% (79/87) achieving ACR50, respectively, at week 372. The ACR70 response rate was numerically higher at most timepoints for patients receiving continuous upadacitinib compared with those receiving continuous adalimumab (75.4% [172/228] vs 68.6% [59/86], respectively, at week 372) (AO data; figure 3 and online supplemental table S8).

When analysed using NRI, a consistently higher proportion of patients initially randomised to upadacitinib achieved ACR20, ACR50 and ACR70 responses over the whole 7-year period compared with those initially randomised to adalimumab. At week 372, ACR20 was achieved by 32.1% (209/651) vs 25.1% (82/327); ACR50 was achieved by 30.0% (195/651) vs 22.9% (75/327); and ACR70 was achieved by 25.7% (167/651) vs 17.1% (56/327) of patients on upadacitinib compared with those on adalimumab, respectively (NRI data; online supplemental figure S2 and online supplemental table S12).

When looking across timepoints at changes from baseline in ACR components, including TJC68, SJC66, PtGA, PhGA, PtPain (0–100 mm scale), HAQ-DI (0–3 scale) and hsCRP (mg/L), patients on continuous upadacitinib showed numerically greater improvements in most ACR components compared with continuous adalimumab, with the exception of TJC68, for which improvements were similar across timepoints. The mean changes from baseline in TJC68, SJC66, PtGA, PhGA, PtPain, HAQ-DI and hsCRP at week 372 are shown in online supplemental figure S3 (AO data).

#### Patients receiving rescue therapy

**Figure 4:**
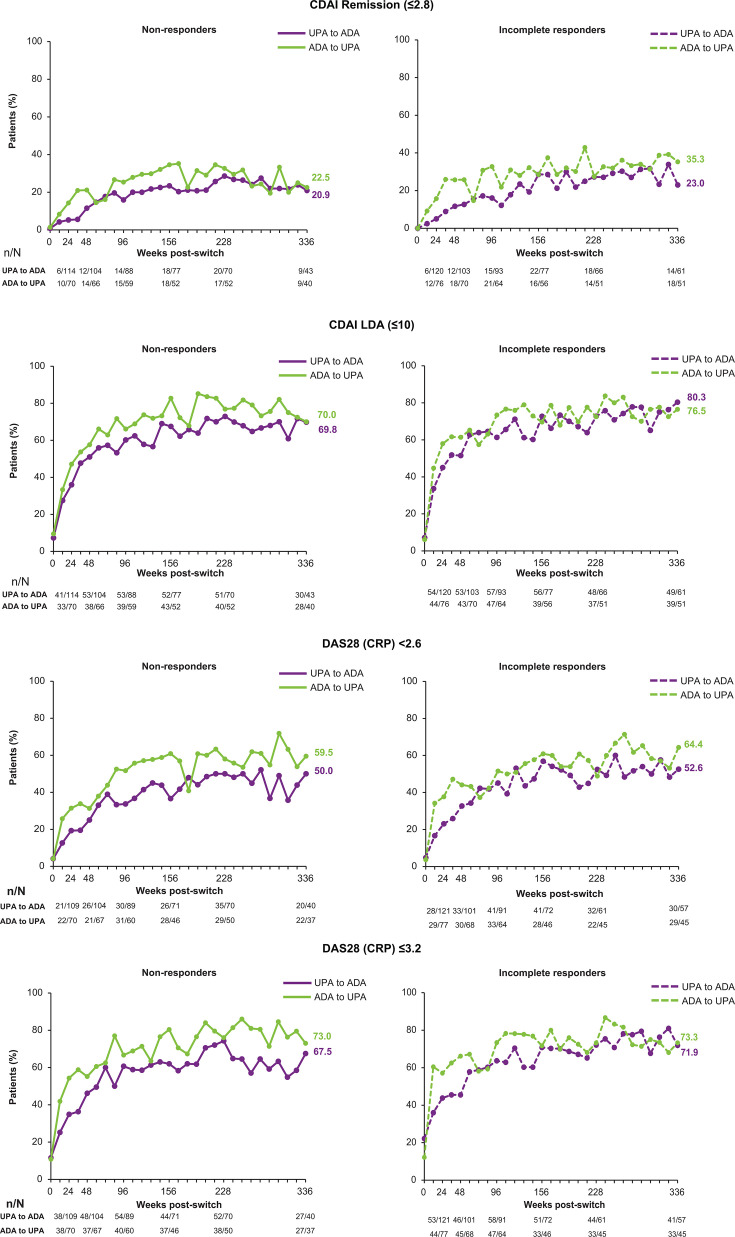
Proportions of patients who were non-responders or incomplete responders to the initial therapy and switched to the alternate therapy by week 26, who achieved CDAI LDA (≤10), CDAI remission (≤2.8), DAS28(CRP) ≤3.2 and DAS28(CRP) <2.6 through 336 weeks post switch (AO). Sample sizes (n) are shown below each graph. Data points plotted here are shown in online supplemental table S9. ADA, adalimumab; AO, as observed; CDAI, Clinical Disease Activity Index; DAS28(CRP), 28-joint Disease Activity Score based on C reactive protein; LDA, low disease activity; UPA, upadacitinib.

In patients who switched from upadacitinib to adalimumab and from adalimumab to upadacitinib, the group switching to upadacitinib exhibited overall numerically higher CDAI and DAS28(CRP) responses across timepoints and at week 336 post switch: CDAI remission, 22.5% (9/40) vs 20.9% (9/43) for the NR subgroup and 35.3% (18/51) vs 23.0% (14/61) for the IR subgroup; CDAI LDA, 70.0% (28/40) vs 69.8% (30/43) for the NR subgroup and 76.5% (39/51) vs 80.3% (49/61) for the IR subgroup; DAS28(CRP) <2.6, 59.5% (22/37) vs 50.0% (20/40) for the NR subgroup and 64.4% (29/45) vs 52.6% (30/57) for the IR subgroup; and DAS28(CRP) ≤3.2, 73.0% (27/37) vs 67.5% (27/40) for the NR subgroup and 73.3% (33/45) vs 71.9% (41/57) for the IR subgroup, respectively, at week 336 post switch (AO data; figure 4 and online supplemental table S9).

**Figure 5:**
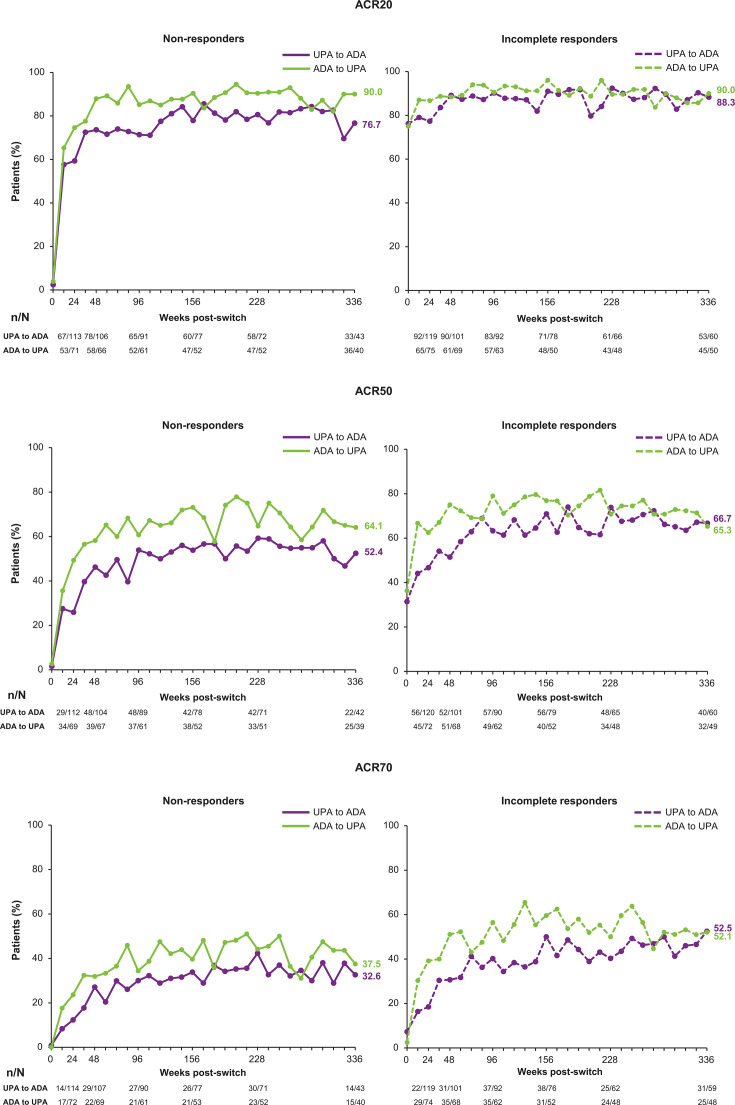
Proportions of patients who were non-responders or incomplete responders to the initial therapy and switched to the alternate therapy by week 26, who achieved ACR20, ACR50 and ACR70 through 336 weeks post switch (AO). Sample sizes (n) are shown below each graph. Data points plotted here are shown in online supplemental table S10. ACR20/50/70, ≥20/50/70% improvement in American College of Rheumatology response criteria; ADA, adalimumab; AO, as observed; UPA, upadacitinib.

The group switching from adalimumab to upadacitinib exhibited overall numerically higher ACR20, ACR50 and ACR70 responses compared with the group switching from upadacitinib to adalimumab across timepoints and at week 336 post switch, with more notable differences observed in the NR compared with the IR subgroup: ACR20, 90.0% (36/40) vs 76.7% (33/43); ACR50, 64.1% (25/39) vs 52.4% (22/42); and ACR70, 37.5% (15/40) vs 32.6% (14/43) in the group switching to upadacitinib vs the group switching to adalimumab, respectively, at week 336 post switch (AO data; figure 5 and online supplemental table S10).

In patients who switched from adalimumab to upadacitinib and from upadacitinib to adalimumab, improvements from baseline were observed in all ACR components, with the changes being generally numerically greater in the group switching to upadacitinib, and in the IR switch subgroup compared with the NR subgroup. The mean changes from baseline in the adalimumab to upadacitinib versus the upadacitinib to adalimumab groups at week 336 post switch are shown in online supplemental figure S4 and online supplemental table S14 (AO data).

At week 372, the mean (median) cumulative steroid dose for patients who entered the LTE on study drug was the highest in the group that switched from adalimumab to upadacitinib at 18 398.7 (14 661.8) mg, and the lowest in the continuous upadacitinib group at 14 588.3 (13 230.0) mg. The cumulative steroid dose at week 372 was comparable in the other three groups (online supplemental figure S5 and online supplemental table S15).

## Discussion

The analysis of the 7-year data of the ongoing LTE of the SELECT-COMPARE study builds on earlier assessments from this study and provides deeper insights into the long-term safety and efficacy of upadacitinib.^3 13 14 25 27^ To the best of our knowledge, this trial provides the longest-term head-to-head (H2H) evaluation of a JAK inhibitor with an active comparator in patients with RA to date, as the comparator arm of most other H2H studies involving JAK inhibitors has not exceeded 1 year.^34^,^36^ Long-term data, particularly safety considerations, are highly clinically relevant, given that RA is a chronic disease requiring lifelong treatment. In addition, regular reporting with timely dissemination of evidence in long-term clinical trials ensures that the data remain relevant to current clinical practice.

No new safety risks were observed with long-term upadacitinib exposure up to the 7-year cut-off date compared with previously reported SELECT-COMPARE safety data through 3 and 5 years.^3 25^ In addition, the safety profile of upadacitinib was consistent with the results of the SELECT phase III integrated safety analysis, as well as a long-term safety analysis of upadacitinib across indications,^20 21^ with narrow confidence intervals (CIs) for EAERs and EAIRs indicating a high degree of precision of the estimates.

The rates of any TEAEs, serious TEAEs, TEAEs leading to discontinuation and COVID-19-related TEAEs were in line with those reported at 5 years^25^ and were similar between upadacitinib and adalimumab. Higher rates of herpes zoster, CPK elevation, NMSC, lymphopenia and hepatic disorders (mainly ALT/AST elevations) were observed with upadacitinib compared with adalimumab, which is consistent with the known safety profile of JAK inhibitors.^2037^,^40^ Regarding herpes zoster, only a small percentage of patients (~3%) were vaccinated at baseline, and none of the 12 serious events of herpes zoster in this study occurred in vaccinated patients. In a retrospective observational cohort study on the use of the Shingrix vaccine among patients with inflammatory arthritis, herpes zoster occurred in 3.3% (4342/132 672) of patients, of which only 360 cases occurred after vaccination.^41^ These findings support the recommendation of herpes zoster vaccination for all adult patients with inflammatory arthritis, especially those receiving treatment with JAK inhibitors.

No COVID-19-related deaths were reported in the adalimumab group, whereas five deaths from COVID-19 and six deaths from COVID-19 pneumonia (both <0.1 E/100 PY) were reported in the upadacitinib group, which is consistent with previously reported results^42^ and may be explained by the larger number of patients treated with upadacitinib. COVID-19 vaccination is recommended in patients with inflammatory rheumatic diseases, such as RA, receiving immunomodulatory agents.^42^,^44^

The rates of TEAEs of special interest, such as MACE, VTE and malignancy excluding NMSC, were similar between the any upadacitinib and any adalimumab treatment groups. The findings were consistent with those reported in the post hoc analysis evaluating the safety of upadacitinib across the SELECT phase III RA programme, which included patients with baseline risk factors similar to those enrolled in the Oral Rheumatoid Arthritis Trial (ORAL) Surveillance study,^45^ and showed that the incidence of MACE, malignancy excluding NMSC, VTE and mortality was typically higher in patients with increased cardiovascular risk compared with the overall RA patient population, but with comparable rates between upadacitinib and adalimumab.^42^ In addition, a post hoc analysis evaluating the benefit–risk profiles of upadacitinib and adalimumab in patients with lower or higher cardiovascular risk over the short term (~6 months) and long term (~5 years) revealed generally comparable safety risks over 5 years between upadacitinib and adalimumab, except for higher rates of herpes zoster in both risk groups, and higher rates of NMSC and serious infections for upadacitinib-treated patients in the higher CV risk group.^46^ The clinical and functional benefits of upadacitinib relative to adalimumab were observed in both the lower and higher cardiovascular risk groups, providing valuable insights into the benefit–risk balance of upadacitinib and adalimumab for patients with RA.^46^ These findings are particularly important given the need for careful consideration of the benefit–risk profile when prescribing JAK inhibitors to high-risk patients with RA requiring long-term treatment.

Regarding MACE, the results of this study are similar for upadacitinib when compared with adalimumab. These findings are in line with observational data reported in the German Rheumatoide Arthritis - Beobachtung der Biologika-Therapie (RABBIT) register, which are comparable with other available observational data and do not confirm the signal of MACE interpreted from the ORAL Surveillance trial results. Treatment with JAK inhibitors in routine care was not associated with increased risk of MACE, not even in subgroups with higher risk profiles.^47^ Lower rates of MACE were observed in RABBIT in patients treated with upadacitinib (incidence rate: 0.53; 95% CI 0.15 to 1.36) and baricitinib (0.49; 95% CI 0.25 to 0.85) compared with tofacitinib (0.98; 95% CI 0.58 to 1.55).^47^ For malignancies, recent data from the same registry suggest an overall small increase in malignancy risk for JAK inhibitors (predominantly baricitinib and tofacitinib) versus bDMARDs, while rates with upadacitinib appear comparatively lower.^48^ These findings suggest the notion of a potentially safer cardiovascular profile for certain JAK inhibitors, but these observations are preliminary and should be interpreted with caution, as further long-term evidence is needed.

On AO analysis by treatment sequence, similar CDAI LDA and remission responses were observed between continuous upadacitinib and continuous adalimumab during the 7-year LTE period, while numerically higher proportions of patients on continuous upadacitinib achieved DAS28(CRP) ≤3.2 and DAS28(CRP) <2.6 compared with continuous adalimumab at week 372. Similarities in clinical response may be explained by the AO analysis including only patients who continued therapy, likely because they experienced both sustained efficacy and good tolerability, as well as by upadacitinib having a direct effect on CRP, which does not occur with adalimumab.

By contrast, the NRI analysis included all randomised patients, regardless of response or discontinuation of the study drug, and patients who did not achieve CDAI LDA and were rescued at week 26 were considered as NR for all binary endpoints after rescue. Therefore, although the results of the NRI analysis suggest a greater benefit with upadacitinib compared with adalimumab, the response rates are more conservative after week 26 for endpoints less stringent than CDAI LDA.

In patients who were NR or IR to their initial randomised therapy and were rescued by switching to the alternate therapy by week 26, the improvements observed at 228 weeks^13^ were generally maintained across all efficacy endpoints through week 336 post switch, showing the durability of this approach. Numerical differences in these improvements mostly favoured switching from adalimumab to upadacitinib for most outcomes, particularly for ACR20, ACR50 and ACR70 responses in the NR subgroup. Improvements from baseline were also observed in all ACR components in both switch groups and in both the NR and IR subgroups, further supporting the long-term benefit of switching to a therapy with a different mechanism of action (MoA) for patients with RA who fail to meet their treatment goals.

Limitations of this study include not being powered or designed to detect the statistical significance of differences in safety events, and that it was not designed with parallel groups in the LTE due to rescue switching; therefore, caution is advised when interpreting long-term comparative efficacy data. The NRI approach assumes that all missing data represent non-response, which may lead to underestimation of treatment effects over a long follow-up period, particularly when discontinuations are unrelated to lack of efficacy. In addition, NRI does not differentiate between the reasons for missing data. Furthermore, in LTE studies, AO analyses carry an additional risk of bias because patients who remain in the study are often those experiencing sustained benefit and tolerability, thereby inflating observed response rates. In addition, the analysis was not powered for statistical comparisons between switch groups or between the NR and IR groups, and the study design did not include in-class switching. This limits the ability to draw conclusions on whether switching MoA has superior efficacy to an in-class switch. Finally, analysis of disease activity measures did not take into account the potential effects of any modifications to background RA medications, including MTX, on meeting therapeutic targets.

In summary, the safety profile of upadacitinib 15 mg QD throughout the 7-year LTE phase of the ongoing SELECT-COMPARE study was consistent with previously reported findings from SELECT-COMPARE and integrated safety analyses of upadacitinib, with no newly identified safety risks and no discernible trend towards a time-dependent increase in the risk for any events. In addition, upadacitinib exhibited a more favourable efficacy profile compared with adalimumab 40 mg EOW for most outcome measures, including CDAI remission, through 7 years. The long-term treatment benefits and consistent safety profile were extended to patients with RA who did not initially meet their treatment goals and were switched to a drug with a different MoA. The results of this analysis highlight the continued favourable benefit–risk profile of upadacitinib and provide further support for its use for the long-term treatment of patients with RA.

## Supplementary material

10.1136/rmdopen-2025-006657 online supplemental file 1

## Data Availability

Data are available upon reasonable request.

## References

[1] Alivernini S, Firestein GS, McInnes IB (2022). The pathogenesis of rheumatoid arthritis. Immunity.

[2] Di Matteo A, Bathon JM, Emery P (2023). Rheumatoid arthritis. Lancet.

[3] Fleischmann R, Mysler E, Bessette L (2022). Long-term safety and efficacy of upadacitinib or adalimumab in patients with rheumatoid arthritis: results through 3 years from the SELECT-COMPARE study. RMD Open.

[4] McInnes IB, Gravallese EM (2021). Immune-mediated inflammatory disease therapeutics: past, present and future. Nat Rev Immunol.

[5] Tanaka Y (2021). Recent progress in treatments of rheumatoid arthritis: an overview of developments in biologics and small molecules, and remaining unmet needs. Rheumatology (Oxford).

[6] Winthrop KL, Mease P, Kerschbaumer A (2024). Unmet need in rheumatology: reports from the Advances in Targeted Therapies meeting, 2023. Ann Rheum Dis.

[7] Smolen JS (2025). Poor prognostic factors and unmet needs in rheumatoid arthritis. Rheumatology (Oxford).

[8] Fraenkel L, Bathon JM, England BR (2021). 2021 American College of Rheumatology Guideline for the Treatment of Rheumatoid Arthritis. Arthritis Care Res (Hoboken).

[9] Smolen JS, Landewé RBM, Bergstra SA (2023). EULAR recommendations for the management of rheumatoid arthritis with synthetic and biological disease-modifying antirheumatic drugs: 2022 update. Ann Rheum Dis.

[10] Fleischmann R, Meerwein S, Charles-Schoeman C (2024). Efficacy and safety of upadacitinib in patients with rheumatoid arthritis and inadequate response or intolerance to biological treatments: results through 5 years from the SELECT-BEYOND study. RMD Open.

[11] Strand V, Schiff M, Tundia N (2019). Effects of upadacitinib on patient-reported outcomes: results from SELECT-BEYOND, a phase 3 randomized trial in patients with rheumatoid arthritis and inadequate responses to biologic disease-modifying antirheumatic drugs. Arthritis Res Ther.

[12] Szekanecz Z, Buch MH, Charles-Schoeman C (2024). Efficacy and safety of JAK inhibitors in rheumatoid arthritis: update for the practising clinician. Nat Rev Rheumatol.

[13] Fleischmann R, Blanco R, Van den Bosch F (2024). Long-term Efficacy and Safety Following Switch Between Upadacitinib and Adalimumab in Patients with Rheumatoid Arthritis: 5-Year Data from SELECT-COMPARE. Rheumatol Ther.

[14] Fleischmann R, Pangan AL, Song I-H (2019). Upadacitinib Versus Placebo or Adalimumab in Patients With Rheumatoid Arthritis and an Inadequate Response to Methotrexate: Results of a Phase III, Double-Blind, Randomized Controlled Trial. Arthritis Rheumatol.

[15] Biggioggero M, Becciolini A, Crotti C (2019). Upadacitinib and filgotinib: the role of JAK1 selective inhibition in the treatment of rheumatoid arthritis. Drugs Context.

[16] Smolen JS, Pangan AL, Emery P (2019). Upadacitinib as monotherapy in patients with active rheumatoid arthritis and inadequate response to methotrexate (SELECT-MONOTHERAPY): a randomised, placebo-controlled, double-blind phase 3 study. Lancet.

[17] Smolen JS, van der Heijde D, Machold KP (2014). Proposal for a new nomenclature of disease-modifying antirheumatic drugs. Ann Rheum Dis.

[18] Winthrop KL (2017). The emerging safety profile of JAK inhibitors in rheumatic disease. Nat Rev Rheumatol.

[19] Kubo S, Nakayamada S, Tanaka Y (2023). JAK inhibitors for rheumatoid arthritis. Expert Opin Investig Drugs.

[20] Cohen SB, van Vollenhoven RF, Winthrop KL (2021). Safety profile of upadacitinib in rheumatoid arthritis: integrated analysis from the SELECT phase III clinical programme. Ann Rheum Dis.

[21] Burmester GR, Cohen SB, Winthrop KL (2023). Safety profile of upadacitinib over 15 000 patient-years across rheumatoid arthritis, psoriatic arthritis, ankylosing spondylitis and atopic dermatitis. RMD Open.

[22] Burmester GR, Kremer JM, Van den Bosch F (2018). Safety and efficacy of upadacitinib in patients with rheumatoid arthritis and inadequate response to conventional synthetic disease-modifying anti-rheumatic drugs (SELECT-NEXT): a randomised, double-blind, placebo-controlled phase 3 trial. Lancet.

[23] Genovese MC, Fleischmann R, Combe B (2018). Safety and efficacy of upadacitinib in patients with active rheumatoid arthritis refractory to biologic disease-modifying anti-rheumatic drugs (SELECT-BEYOND): a double-blind, randomised controlled phase 3 trial. Lancet.

[24] van Vollenhoven R, Takeuchi T, Pangan AL (2020). Efficacy and Safety of Upadacitinib Monotherapy in Methotrexate-Naive Patients With Moderately-to-Severely Active Rheumatoid Arthritis (SELECT-EARLY): A Multicenter, Multi-Country, Randomized, Double-Blind, Active Comparator-Controlled Trial. Arthritis Rheumatol.

[25] Fleischmann R, Swierkot J, Penn SK (2024). Long-term safety and efficacy of upadacitinib versus adalimumab in patients with rheumatoid arthritis: 5-year data from the phase 3, randomised SELECT-COMPARE study. RMD Open.

[26] Fleischmann RM, Blanco R, Hall S (2021). Switching between Janus kinase inhibitor upadacitinib and adalimumab following insufficient response: efficacy and safety in patients with rheumatoid arthritis. Ann Rheum Dis.

[27] Fleischmann RM, Genovese MC, Enejosa JV (2019). Safety and effectiveness of upadacitinib or adalimumab plus methotrexate in patients with rheumatoid arthritis over 48 weeks with switch to alternate therapy in patients with insufficient response. Ann Rheum Dis.

[28] O’Neil LJ, Alpízar-Rodríguez D, Deane KD (2024). Rheumatoid Arthritis: The Continuum of Disease and Strategies for Prediction, Early Intervention, and Prevention. J Rheumatol.

[29] Aletaha D, Neogi T, Silman AJ (2010). 2010 Rheumatoid arthritis classification criteria: an American College of Rheumatology/European League Against Rheumatism collaborative initiative. Arthritis Rheum.

[30] Aletaha D, Smolen J (2005). The Simplified Disease Activity Index (SDAI) and the Clinical Disease Activity Index (CDAI): a review of their usefulness and validity in rheumatoid arthritis. Clin Exp Rheumatol.

[31] Prevoo ML, van ’t Hof MA, Kuper HH (1995). Modified disease activity scores that include twenty-eight-joint counts. Development and validation in a prospective longitudinal study of patients with rheumatoid arthritis. Arthritis Rheum.

[32] Wells G, Becker J-C, Teng J (2009). Validation of the 28-joint Disease Activity Score (DAS28) and European League Against Rheumatism response criteria based on C-reactive protein against disease progression in patients with rheumatoid arthritis, and comparison with the DAS28 based on erythrocyte sedimentation rate. Ann Rheum Dis.

[33] Felson DT, Anderson JJ (1995). Methodological and statistical approaches to criteria development in rheumatic diseases. Baillieres Clin Rheumatol.

[34] Combe B, Kivitz A, Tanaka Y (2021). Filgotinib versus placebo or adalimumab in patients with rheumatoid arthritis and inadequate response to methotrexate: a phase III randomised clinical trial. Ann Rheum Dis.

[35] Taylor PC, Keystone EC, van der Heijde D (2017). Baricitinib versus Placebo or Adalimumab in Rheumatoid Arthritis. N Engl J Med.

[36] Fleischmann R, Mysler E, Hall S (2017). Efficacy and safety of tofacitinib monotherapy, tofacitinib with methotrexate, and adalimumab with methotrexate in patients with rheumatoid arthritis (ORAL Strategy): a phase 3b/4, double-blind, head-to-head, randomised controlled trial. Lancet.

[37] Charles-Schoeman C, Buch MH, Dougados M (2023). Risk of major adverse cardiovascular events with tofacitinib versus tumour necrosis factor inhibitors in patients with rheumatoid arthritis with or without a history of atherosclerotic cardiovascular disease: a post hoc analysis from ORAL Surveillance. Ann Rheum Dis.

[38] Cohen SB, Tanaka Y, Mariette X (2017). Long-term safety of tofacitinib for the treatment of rheumatoid arthritis up to 8.5 years: integrated analysis of data from the global clinical trials. Ann Rheum Dis.

[39] Smolen JS, Genovese MC, Takeuchi T (2019). Safety Profile of Baricitinib in Patients with Active Rheumatoid Arthritis with over 2 Years Median Time in Treatment. J Rheumatol.

[40] Sunzini F, McInnes I, Siebert S (2020). JAK inhibitors and infections risk: focus on herpes zoster. Ther Adv Musculoskelet Dis.

[41] Curtis JR, Conrad DM, Krueger WS (2025). Real-world data on the use of the Shingrix vaccine among patients with inflammatory arthritis and risk of cardiovascular events following herpes zoster. Arthritis Res Ther.

[42] Fleischmann R, Curtis JR, Charles-Schoeman C (2023). Safety profile of upadacitinib in patients at risk of cardiovascular disease: integrated post hoc analysis of the SELECT phase III rheumatoid arthritis clinical programme. Ann Rheum Dis.

[43] Yap RXL, Lai YW, Wei C (2024). Impact of Immunomodulatory Therapy on COVID-19 Vaccine Response in Patients with Autoimmune Inflammatory Rheumatic Diseases. *Vaccines (Basel*).

[44] Bass AR, Chakravarty E, Akl EA (2023). 2022 American College of Rheumatology Guideline for Vaccinations in Patients With Rheumatic and Musculoskeletal Diseases. Arthritis Care &Amp; Research.

[45] Ytterberg SR, Bhatt DL, Mikuls TR (2022). Cardiovascular and Cancer Risk with Tofacitinib in Rheumatoid Arthritis. N Engl J Med.

[46] Burmester GR, Mysler E, Taylor P (2025). Benefit-risk analysis of upadacitinib versus adalimumab in patients with rheumatoid arthritis and higher or lower risk of cardiovascular disease. RMD Open.

[47] Meissner Y, Schäfer M, Albrecht K (2023). Risk of major adverse cardiovascular events in patients with rheumatoid arthritis treated with conventional synthetic, biologic and targeted synthetic disease-modifying antirheumatic drugs: observational data from the German RABBIT register. RMD Open.

[48] Schaefer M, Purschke A, Zietemann V (2025). Comparative risk of incident malignancies in rheumatoid arthritis patients treated with Janus kinase inhibitors or bDMARDs: observational data from the German RABBIT register. Ann Rheum Dis.

