# Supplementary material for: Long-term safety and efficacy of upadacitinib compared with adalimumab in patients with rheumatoid arthritis: 7-year data from the SELECT-COMPARE study
Source: RMD Open. 2026 Jun 24;12(2):e006657. doi: 10.1136/rmdopen-2025-006657 (PMC13296005; doi:10.1136/rmdopen-2025-006657)
Supplement: online supplemental file 1 [file rmdopen-12-2-s001.docx]

**Long-term safety and efficacy of upadacitinib compared with adalimumab in patients with rheumatoid arthritis: 7-year data from the SELECT-COMPARE study**

Roy Fleischmann,^1,2^ Jerzy Swierkot,^3^ Patrick Durez,^4^ Louis Bessette,^5^ Ricardo Blanco,^6^ Filip Van den Bosch,^7^ Duke Geem,^8^ Lingfeng Luo,^8^ Lauren D. Smith,^8^ Diane Caballero,^8^ Sebastian Meerwein,^9^ Charles Peterfy,^10^ Yoshiya Tanaka,^11^ Eduardo Mysler^12^

^1^University of Texas Southwestern Medical Center, Dallas, TX, USA

^2^Metroplex Clinical Research Center, Dallas, TX, USA

^3^Wroclaw Medical University, Department of Rheumatology and Internal Medicine, Wroclaw, Poland

^4^Institut de Recherche Expérimentale et Clinique, UCLouvain Saint-Luc, Pôle de Recherche en Rhumatologie, Brussels, Belgium

^5^Centre de recherche du CHU de Québec, Laval University, Québec, Canada

^6^Rheumatology Division, Hospital University Marqués de Valdecilla, Immunopathology Group, IDIVAL, Santander, Spain

^7^VIB-UGent Center for Inflammation Research, Department of Internal Medicine and Pediatrics, Ghent University, Ghent, Belgium

^8^AbbVie, Immunology, North Chicago, IL, USA

^9^AbbVie Deutschland GmbH & Co KG, Ludwigshafen, Germany

^10^Spire Sciences Inc, Boca Raton, FL, USA

^11^Department of Molecular Targeted Therapeutics, University of Occupational and Environmental Health, Kitakyushu, Japan

^12^Organización Medica de Investigación, Rheumatology, Buenos Aires, Argentina

**Correspondence to:** Professor Roy Fleischmann

Metroplex Clinical Research Center, 8144 Walnut Hill Lane, Suite 810, Dallas, TX 75231, USA

Email: [rfleischmann@arthdocs.com](mailto:rfleischmann@arthdocs.com)

**SUPPLEMENTARY METHODS**

**Calculation of cumulative steroid dose** (all patients in the full analysis set)

(i) Prednisone daily equivalent steroid use was calculated based on records in the ADCM data set with CMCOCORT = ‘Y’ and CMROUTE in (‘INTRA-ARTICULAR’, ‘INTRASYNOVIAL’, ‘INTRAVENOUS’, ‘INTRAVENOUS BOLUS’, ‘INTRAVENOUS DRIP’, ‘ORAL’, ‘OROPHARYNGEAL’, ‘PERIARTICULAR’).
(ii) Cumulative steroid usage was calculated at each analysis visit until the target date by summing up prednisone daily equivalent steroid dose from baseline to target dates of each analysis visit up to week 372. For each steroid record, prednisone daily equivalent dose will be calculated using the formula:

$\frac{Oral Steroid dose}{Equivalent dose}\times5 mg\times Frequency$ (a)

**Equivalent steroid dose**

| **Steroid** | **Equivalent dose (mg) used in the formula** |
| --- | --- |
| Beclomethasone | 5 |
| Betamethasone/betamethasone sodium phosphate/betamethasone dipropionate; betamethasone sodium phosphate/betamethasone acetate; betamethasone sodium phosphate; bupivacaine hydrochloride /betamethasone dipropionate: betamethasone sodium phosphate/betamethasone phosphate/betamethasone; chlorphenamine maleate/betamethasone; cyanocobalamin; diclofenac sodium/betamethasone; desloratadine/betamethasone; indomethacin; methocarbamol/betamethasone; loratadine | 0.6 |
| Budesonide | 1 |
| Cortisone/cortisone acetate | 25 |
| Deflazacort | 7.5 |
| Dexamethasone/dexamethasone palmitate/dexamethasone sodium metasulfobenzoate/dexamethasone sodium phosphate/chlorphenamine; dexamethasone/dexamethasone acetate; dexamethasone sodium phosphate | 0.75 |
| Hydrocortisone/hydrocortisone sodium succinate/hydrocortisone acetate | 20 |
| Meprednisone | 5 |
| Methylprednisolone/methylprednisolone acetate/methylprednisolone sodium succinate/lidocaine; methylprednisolone acetate/lidocaine hydrochloride; methylprednisolone acetate | 4.0 |
| Prednisolone/prednisolone farnesylate/prednisolone acetate/prednisolone hemisuccinate/prednisolone metasulfobenzoate sodium/prednisone/prednisone acetate/prednisolone sodium succinate/phenylbutazone; prednisolone/prednisolone sodium phosphate | 5 |
| Triamcinolone/triamcinolone acetonide/triamcinolone hexacetonide | 4.0 |

**Equivalent dose frequency**

In general, the dose frequency was converted to daily dose frequency, which was used as a factor in equation (a). If the frequency could not be quantified (eg, Occasional) after site query and/or medical adjudication, the record was not used in the calculation.

| **Dosing frequency per interval in the database** | **Definition** | **Equivalent daily dose frequency used in formula** |
| --- | --- | --- |
| QD | Once a day | 1 |
| BID | Twice a day | 2 |
| TID | Three times a day | 3 |
| QID | Four times a day | 4 |
| QAM | Every morning | 1 |
| QM | Every morning | 1 |
| Every afternoon |  | 1 |
| QPM | Every evening | 1 |
| QHS | Every night at bedtime | 1 |
| Q8H | Every 8 hours | 3 |
| Q12H | Every 12 hours | 2 |
| Q24H | Every 24 hours | 1 |
| Q48H | Every 48 hours | 0.5 |
| Q3D | Every 3 days | 1/3 |
| QOD | Every other day | 0.5 |
| 1 time per week |  | 1/7 |
| 2 times per week |  | 2/7 |
| 3 times per week |  | 3/7 |
| 4 times per week |  | 4/7 |
| 5 times per week |  | 5/7 |
| 6 times per week |  | 6/7 |
| 7 times per week |  | 1 |
| Every 2 weeks |  | 1/14 |
| Every 4 weeks |  | 1/28 |
| Every 12 weeks |  | 1/84 |
| PRN | As needed | 1 |
| Once |  | 1 |

**SUPPLEMENTARY TABLES AND FIGURES**

**Table S1**  Patients with cardiovascular risk factors at baseline (safety analysis set)

| **Number of patients (%)** | **Placebo**  (N=652) | | **Upadacitinib**  **15 mg QD**  (N=650) | | **Adalimumab**  **40 mg EOW**  (N=327) | |
| --- | --- | --- | --- | --- | --- | --- |
|  | All patients | ≥50 years  (n=432) | All patients | ≥50 years  (n=445) | All patients | ≥50 years  (n=223) |
| Patients with ≥1 CV risk factor | 478 (73.3) | 350 (53.7) | 505 (77.7) | 382 (58.8) | 241 (73.7) | 193 (59.0) |
| 1 | 209 (32.1) | 134 (20.6) | 248 (38.2) | 175 (26.9) | 114 (34.9) | 83 (25.4) |
| 2 | 178 (27.3) | 138 (21.2) | 175 (26.9) | 132 (20.3) | 84 (25.7) | 71 (21.7) |
| ≥3 | 91 (14.0) | 78 (12.0) | 82 (12.6) | 75 (11.5) | 43 (13.1) | 39 (11.9) |
| CV risk factors |  |  |  |  |  |  |
| Hypertension | 241 (37.0) | 208 (31.9) | 276 (42.5) | 233 (35.8) | 136 (41.6) | 123 (37.6) |
| DM | 45 (6.9) | 38 (5.8) | 53 (8.2) | 43 (6.6) | 19 (5.8) | 17 (5.2) |
| Smoking^*^ | 218 (33.4) | 157 (24.1) | 225 (34.6) | 159 (24.5) | 118 (36.1) | 97 (29.7) |
| High LDL-C^†^ | 187 (28.7) | 135 (20.7) | 178 (27.4) | 143 (22.0) | 91 (27.8) | 73 (22.3) |
| Low HDL-C^‡^ | 88 (13.5) | 56 (8.6) | 61 (9.4) | 36 (5.5) | 29 (8.9) | 19 (5.8) |
| History of CV event | 79 (12.1) | 67 (10.3) | 81 (12.5) | 78 (12.0) | 26 (8.0) | 20 (6.1) |

^*^Current or former smoker. ^†^LDL-C >100 mg/dL. ^‡^HDL-C <40 mg/dL.

CV, cardiovascular; DM, diabetes mellitus; EOW, every other week; HDL-C, high-density lipoprotein cholesterol; LDL-C, low-density lipoprotein cholesterol; QD, once daily.

**Table S2** Most common TEAEs through 372 weeks

| **EAER, E (E/100 PY)** | **Any upadacitinib 15 mg QD**  (N=1417)  PY=6228.0 | **Any adalimumab 40 mg EOW**  (N=579)  PY=2004.3 |
| --- | --- | --- |
| Urinary tract infection | 436 (7.0) | 148 (7.4) |
| Upper respiratory tract infection | 351 (5.6) | 95 (4.7) |
| Nasopharyngitis | 346 (5.6) | 93 (4.6) |
| Bronchitis | 246 (3.9) | 86 (4.3) |
| COVID-19 | 242 (3.9) | 95 (4.7) |
| CPK elevation | 221 (3.5) | 33 (1.6) |
| ALT elevation | 219 (3.5) | 45 (2.2) |
| RA | 218 (3.5) | 98 (4.9) |
| Hypertension | 215 (3.5) | 61 (3.0) |
| AST elevation | 172 (2.8) | 31 (1.5) |
| Leukopenia | 171 (2.7) | 29 (1.4) |
| Lymphopenia | 153 (2.5) | 17 (0.8) |

Safety was assessed up to week 372, through the cut-off date of 19 September 2024. TEAEs included any adverse event with an onset date on or after the first dose of study drug and up to 30 days after the last dose of placebo or upadacitinib and 70 days for ADA, if patients discontinued prematurely. Data through 372 weeks include all patients receiving upadacitinib or ADA, including rescue groups, with assignment based on drug exposure at the time of the event.

ADA, adalimumab; ALT, alanine aminotransferase; AST, aspartate aminotransferase; CPK, creatine phosphokinase; E, event; EAER, exposure-adjusted event rate; EOW, every other week; PY, patient-years; QD, once daily; RA, rheumatoid arthritis; TEAE, treatment-emergent adverse event.

**Table S3** Treatment-emergent malignancies, adjudicated MACE and adjudicated VTE

| **E (E/100 PY)** | **Any upadacitinib**  **15 mg QD**  (N=1417)  PY=6228.0 | **Any adalimumab**  **40 mg EOW**  (N=579)  PY=2004.3 |
| --- | --- | --- |
| **Malignancies (SOC MedDRA 27.0 preferred term)** | | |
| **Gastrointestinal** |  |  |
| Adenocarcinoma gastric | 1 (<0.1) | 1 (<0.1) |
| Adenocarcinoma of colon | 2 (<0.1) | 1 (<0.1) |
| Anal cancer | 1 (<0.1) | 0 |
| Colon cancer | 1 (<0.1) | 2 (<0.1) |
| Colon cancer metastatic | 0 | 1 (<0.1) |
| Gastric cancer | 1 (<0.1) | 0 |
| Rectal adenocarcinoma | 1 (<0.1) | 0 |
| **Skin** |  |  |
| Basal cell carcinoma* | 15 (0.2) | 2 (<0.1) |
| Squamous cell carcinoma of skin** | 13 (0.2) | 1 (<0.1) |
| Malignant melanoma | 3 (<0.1) | 1 (<0.1) |
| Lentigo maligna | 0 | 1 (<0.1) |
| Superficial spreading melanoma stage I | 0 | 1 (<0.1) |
| Bowen's disease | 1 (<0.1) | 0 |
| Carcinoma *in situ* of skin | 1 (<0.1) | 0 |
| Neuroendocrine carcinoma of the skin | 1 (<0.1) | 0 |
| Skin squamous cell carcinoma metastatic | 1 (<0.1) | 0 |
| **Breast and gynaecological** |  |  |
| Breast cancer | 2 (<0.1) | 0 |
| Breast cancer metastatic | 0 | 1 (<0.1) |
| Invasive ductal breast carcinoma | 1 (<0.1) | 0 |
| Intraductal proliferative breast lesion | 1 (<0.1) | 0 |
| Endometrial adenocarcinoma | 1 (<0.1) | 0 |
| Ovarian endometrioid carcinoma | 1 (<0.1) | 0 |
| Squamous cell carcinoma of the cervix | 2 (<0.1) | 0 |
| Squamous cell carcinoma of the vulva | 1 (<0.1) | 0 |
| Vulval cancer | 1 (<0.1) | 0 |
| **Lung** |  |  |
| Lung adenocarcinoma | 5 (<0.1) | 0 |
| Large cell lung cancer | 0 | 1 (<0.1) |
| Lung carcinoma cell type unspecified stage IV | 1 (<0.1) | 0 |
| Lung neoplasm malignant | 1 (<0.1) | 0 |
| Lung squamous cell carcinoma stage IV | 0 | 1 (<0.1) |
| Small cell lung cancer | 0 | 1 (<0.1) |
| Small cell lung cancer metastatic | 1 (<0.1) | 0 |
| Squamous cell carcinoma of lung | 1 (<0.1) | 0 |
| **Haematological** |  |  |
| B-cell lymphoma | 0 | 1 (<0.1) |
| Diffuse large B-cell lymphoma | 0 | 1 (<0.1) |
| Hodgkin's disease stage IV | 0 | 1 (<0.1) |
| **Kidney and urinary tract** |  |  |
| Bladder transitional cell carcinoma | 1 (<0.1) | 0 |
| Clear cell renal cell carcinoma | 1 (<0.1) | 0 |
| Renal cell carcinoma | 0 | 1 (<0.1) |
| **Other** |  |  |
| Squamous cell carcinoma (SOC not specified) | 1 (<0.1) | 0 |
| Adenocarcinoma (SOC not specified) | 1 (<0.1) | 0 |
| Prostate cancer | 1 (<0.1) | 0 |
| Squamous cell carcinoma of the oral cavity | 1 (<0.1) | 0 |
| Glioblastoma | 1 (<0.1) | 0 |
| Laryngeal cancer | 1 (<0.1) | 0 |
| Malignant neoplasm of unknown primary site | 1 (<0.1) | 0 |
| Metastases to bone | 0 | 1 (<0.1) |
| Metastases to bone marrow | 0 | 1 (<0.1) |
| Metastases to lymph nodes | 1 (<0.1) | 0 |
| Myxoid liposarcoma | 1 (<0.1) | 0 |
| **Adjudicated MACE and other CV events** | | |
| Any adjudicated CV event | 39 (0.6) | 15 (0.7) |
| MACE^††^ | 19 (0.3) | 8 (0.4) |
| Cardiovascular death | 8 (0.1) | 2 (<0.1) |
| Fatal acute myocardial infarction | 1 (0.1) | 0 |
| Sudden cardiac death | 6 (<0.1) | 1 (<0.1) |
| Heart failure | 0 | 1 (<0.1) |
| Cardiovascular procedure-related death | 0 | 0 |
| Death due to cardiovascular haemorrhage | 0 | 0 |
| Fatal stroke | 0 | 0 |
| Pulmonary embolism^††^ | 1 (<0.1) | 0 |
| Venous embolism^††^ | 0 | 0 |
| Non-cardiac/non-neurologic arterial  thrombosis | 0 | 0 |
| Other | 0 | 0 |
| Non-fatal myocardial infarction | 6 (<0.1) | 2 (<0.1) |
| Non-fatal stroke | 5 (<0.1) | 4 (0.2) |
| Undetermined/UNK cause of death | 8 (0.1) | 1 (<0.1) |
| Other cardiovascular events (non-fatal) | 12 (0.2) | 6 (0.3) |
| Cardiovascular procedures | 2 (<0.1) | 2 (<0.1) |
| Unstable angina requiring hospitalisation | 1 (<0.1) | 0 |
| Hospital-based treatment for heart failure | 5 (<0.1) | 2 (<0.1) |
| Transient ischaemic attack | 4 (<0.1) | 2 (<0.1) |
| **Adjudicated VTE** | | |
| Any adjudicated thrombotic events | 19 (0.3) | 7 (0.3) |
| Fatal^‡^ | 1 (<0.1) | 0 |
| Pulmonary embolism | 1 (<0.1) | 0 |
| Venous thrombosis | 0 | 0 |
| Non-fatal^‡‡^ | 18 (0.3) | 7 (0.3) |
| Deep vein thrombosis (DVT) | 10 (0.2) | 3 (0.1) |
| Pulmonary embolism (PE) | 12 (0.2) | 4 (0.2) |
| DVT and PE (concurrent) | 4 (<0.1) | 0 |

Safety was assessed up to week 372, through the cut-off date of 19 September 2024. TEAEs included any adverse event with an onset date on or after the first dose of study drug and up to 30 days after the last dose of placebo or upadacitinib and 70 days for adalimumab, if patients discontinued prematurely.

Note: patients in the switch groups experiencing events may be listed under both any upadacitinib and any adalimumab; more than one events were observed in some patients.

*18 BCCs in 16 patients who received at least one dose of upadacitinib and 5 BCCs in 3 patients who received at least one dose of adalimumab.

**22 SCCs in 13 patients who received at least one dose of upadacitinib.

^†^Defined as cardiovascular death, non-fatal myocardial infarction and non-fatal stroke.

^††^Pulmonary embolism and venous thrombosis (under cardiovascular death) is also included in the adjudicated VTE section of the table.

^‡^VTEs (fatal) are also counted under cardiovascular death in the adjudicated MACE section of the table.

^‡‡^VTEs (non-fatal) include deep DVT and PE. Concurrent DVTs and PEs are counted in the ‘DVT’ and ‘PE’ sections of the table, as well as in the ‘DVT and PE (concurrent)’ section of the table.

CV, cardiovascular; DVT, deep vein thrombosis; E, event; EOW, every other week; MACE, major adverse cardiovascular events; MedDRA, Medical Dictionary for Regulatory Activities; PE, pulmonary embolism; PY, patient-years; QD, once daily; SOC, System Organ Class; TEAE, treatment-emergent adverse event; UNK, unknown; VTE, venous thromboembolic events.

**Table S4** Grade 3 or 4 laboratory abnormalities through 372 weeks

| **Parameter** | **Any upadacitinib 15 mg QD**  (N=1417)  % of patients | **Any adalimumab 40 mg EOW**  (N=579)  % of patients |
| --- | --- | --- |
| Haemoglobin (g/L) |  |  |
| Grade 3 (decrease 21–29**^*^** or Hb ≥70 to <80) | 10.1 | 6.8 |
| Grade 4 (decrease ≥30**^*^** or Hb <70) | 5.4 | 5.6 |
| Lymphocytes (×10^9^/L) |  |  |
| Grade 3 (0.5 to <1.0) | 37.4 | 13.2 |
| Grade 4 (<0.5) | 6.9 | 1.4 |
| Neutrophils (×10^9^/L) |  |  |
| Grade 3 (0.5 to <1.0) | 2.3 | 1.2 |
| Grade 4 (<0.5) | 0.4 | 0.5 |
| ALT (U/L) |  |  |
| Grade 3 (3.0–8.0×ULN) | 7.9 | 3.3 |
| Grade 4 (>8.0×ULN) | 1.1 | 1.0 |
| AST (U/L) |  |  |
| Grade 3 (3.0–8.0×ULN) | 5.0 | 1.7 |
| Grade 4 (>8.0×ULN) | 0.9 | 1.6 |
| CPK (U/L) |  |  |
| Grade 3 (>5.0–10.0×ULN) | 3.2 | 1.2 |
| Grade 4 (>10.0×ULN) | 1.3 | 0.7 |
| Creatinine (μmol/L) |  |  |
| Grade 3 (>3.0–6.0×ULN) | 0.2 | 0.5 |
| Grade 4 (>6.0×ULN) | 0.1 | 0.0 |

Safety was assessed up to week 372, through the cut-off date of 19 September 2024. Data are for patients with worsening in grade severity for laboratory parameters. Grading is based on Outcome Measures in Rheumatology criteria, except for CPK and creatinine, for which National Cancer Institute Common Terminology Criteria were used.

**^*^**Decrease from baseline. Baseline is defined as the last observation on or before the date of the first dose of study drug in the corresponding treatment group.

ALT, alanine aminotransferase; AST, aspartate aminotransferase; CPK, creatine phosphokinase; EOW, every other week; Hb, haemoglobin; QD, once daily; ULN, upper limit of normal.

**Table S5** Treatment-emergent adverse events leading to death through 372 weeks

| **System organ class MedDRA 27.0 preferred term, E (E/100 PY)** | **Any upadacitinib 15 mg QD**  (N=1417)  PY=6228.0 | **Any adalimumab 40 mg EOW**  (N=579)  PY=2004.3 |
| --- | --- | --- |
| Any AE | 45 (0.7) | 14 (0.7) |
| Cardiac disorders | 14 (0.2) | 2 (<0.1) |
| Acute myocardial infarction | 3 (<0.1) | 0 |
| Cardiac arrest | 1 (<0.1) | 0 |
| Cardiac failure | 4 (<0.1) | 0 |
| Cardiorespiratory arrest | 2 (<0.1) | 0 |
| Cardiogenic shock | 1 (<0.1) | 0 |
| Cardiopulmonary failure | 0 | 1 (<0.1) |
| Left ventricular failure | 0 | 1 (<0.1) |
| Myocardial infarction | 3 (<0.1) | 0 |
| General disorders and administration site conditions | 8 (0.1) | 2 (<0.1) |
| Death | 4 (<0.1) | 1 (<0.1) |
| Multiple organ dysfunction syndrome | 2 (<0.1) | 1 (<0.1) |
| Sudden death | 2 (<0.1) | 0 |
| Infections and infestations | 17 (0.3) | 4 (0.2) |
| COVID-19 | 5 (<0.1) | 0 |
| COVID-19 pneumonia | 6 (<0.1) | 0 |
| Empyema | 0 | 1 (<0.1) |
| Influenza | 1 (<0.1) | 0 |
| Medical device site joint infection | 0 | 1 (<0.1) |
| Meningitis listeria | 1 (<0.1) | 0 |
| Nosocomial infection | 1 (<0.1) | 0 |
| Pneumonia | 1 (<0.1)* | 1 (<0.1)^†^ |
| Sepsis | 1 (<0.1) | 0 |
| Injury, poisoning and procedural complications | 1 (<0.1) | 1 (<0.1) |
| Craniocerebral injury | 0 | 1 (<0.1) |
| Pelvic fracture | 1 (<0.1) | 0 |
| Musculoskeletal and connective tissue disorders | 0 | 1 (<0.1) |
| Mixed connective tissue disease | 0 | 1 (<0.1) |
| Neoplasms benign, malignant and unspecified (including cysts and polyps) | 3 (<0.1) | 3 (0.1) |
| Colon cancer metastatic | 0 | 1 (<0.1) |
| Diffuse large B-cell lymphoma | 0 | 1 (<0.1) |
| Lung carcinoma cell type unspecified stage IV | 1 (<0.1) | 0 |
| Lung squamous cell carcinoma stage IV | 0 | 1 (<0.1) |
| Malignant neoplasm of unknown primary | 1 (<0.1) | 0 |
| Myelodysplastic syndrome with excess blasts | 1 (<0.1) | 0 |
| Respiratory, thoracic and mediastinal disorders | 2 (<0.1) | 0 |
| Acute respiratory distress syndrome | 1 (<0.1) | 0 |
| Respiratory failure^‡^ | 1 (<0.1) | 0 |

Safety was assessed up to week 372, through the cut-off date of 19 September 2024. Treatment-emergent adverse events included any AE with an onset date on or after the first dose of study drug and up to 30 days after the last dose of placebo or upadacitinib and 70 days for adalimumab, if patients discontinued prematurely. Data through 372 weeks include all patients receiving upadacitinib or adalimumab, including rescue groups, with assignment based on drug exposure at the time of the event.

*Pneumonia bacterial.

^†^Pneumonia legionella.

^‡^Respiratory failure related to COVID-19.

AE, adverse event; E, events; EOW, every other week; MedDRA, Medical Dictionary for Regulatory Activities; PY, patient-years; QD, once daily.

**Table S6** Exposure-adjusted incidence rates of treatment-emergent adverse events through 372 weeks

| **EAIR (n/100 PY)**  **[95% CI]** | **Any upadacitinib 15 mg QD**  **(N=1417)** | **Any adalimumab 40 mg EOW (N=579)** |
| --- | --- | --- |
| Any TEAE | 110.3 [104.3, 116.6] | 126.3 [115.1, 138.3] |
| Serious TEAE | 7.7 [7.0, 8.5] | 8.8 [7.5, 10.4] |
| TEAE leading to study drug D/C | 3.5 [3.1, 4.0] | 4.2 [3.4, 5.2] |
| Any COVID-19-related AE | 4.7 [4.2, 5.3] | 5.0 [4.0, 6.1] |
| Any death**^*^** | 0.7 [0.5, 0.9] | 0.6 [0.3, 1.1] |
| Serious infection | 2.7 [2.3, 3.1] | 2.4 [1.8, 3.2] |
| Opportunistic infection^†^ | 0.2 [0.1, 0.3] | 0.2 [0.0, 0.4] |
| Herpes zoster | 2.3 [1.9, 2.7] | 0.8 [0.5, 1.3] |
| Active tuberculosis | <0.1 [0.0, 0.1] | 0.2 [0.1, 0.5] |
| Malignancy (excluding NMSC) | 0.6 [0.4, 0.8] | 0.8 [0.5, 1.3] |
| NMSC | 0.4 [0.2, 0.6] | 0.8 [0.5, 1.3] |
| Lymphoma | 0 | 0.1 [0.0, 0.4] |
| Adjudicated MACE^‡^ | 0.3 [0.2, 0.5] | 0.4 [0.2, 0.8] |
| Adjudicated VTE^**^ | 0.3 [0.2, 0.5] | 0.3 [0.1, 0.7] |
| Adjudicated GI perforation | <0.1 [0.0, 0.1] | 0 |
| Renal dysfunction | 0.2 [0.1, 0.4] | 0.2 [0.2, 0.5] |
| Anaemia | 2.0 [1.7, 2.4] | 2.4 [1.7, 3.2] |
| Lymphopenia | 1.5 [1.2, 1.9] | 0.5 [0.2, 0.9] |
| Neutropenia | 1.5 [1.2, 1.9] | 1.2 [0.8, 1.8] |
| CPK elevation | 2.3 [1.9, 2.7] | 1.2 [0.8, 1.8] |
| Hepatic disorder | 4.6 [4.0, 5.2] | 2.9 [2.2, 3.8] |

Safety was assessed up to week 372, through the cut-off date of 19 September 2024. TEAEs included any adverse event with an onset date on or after the first dose of study drug and up to 30 days after the last dose of placebo or upadacitinib and 70 days for adalimumab, if patients discontinued prematurely. Data are for all patients receiving upadacitinib or adalimumab, including exposure to rescue treatment after switching, with assignment based on drug exposure at time of event.

**^*^**Includes treatment-emergent (occurring ≤30 days after the last dose of upadacitinib or ≤70 days after the last dose of adalimumab).

^†^Excluding tuberculosis and herpes zoster.

^‡^Defined as cardiovascular death (includes acute myocardial infarction, sudden cardiac death, heart failure, cardiovascular procedure-related death, death due to cardiovascular haemorrhage, fatal stroke, pulmonary embolism and other cardiovascular causes), non-fatal myocardial infarction and non-fatal stroke.

^**^Includes deep vein thrombosis and pulmonary embolism (fatal and non-fatal).

CI, confidence interval; CPK, creatine phosphokinase; E, event; EAIR, exposure-adjusted incidence rate; EOW, every other week; GI, gastrointestinal; MACE, major adverse cardiovascular events; NMSC, non-melanoma skin cancer; PY, patient-years; QD, once daily; TEAE, treatment-emergent adverse event; VTE, venous thromboembolic events.

**Table S7** Proportions of patients achieving CDAI LDA (≤10)/remission (≤2.8), DAS28(CRP) ≤3.2/<2.6 through 372 weeks (AO)

| **Weeks** | | | | | | | | | | | |
| --- | --- | --- | --- | --- | --- | --- | --- | --- | --- | --- | --- |
| **n/N (%)** | **12** | **26** | **48** | **60** | **72** | **84** | **96** | **108** | **120** | **132** | **144** |
| **CDAI ≤10** |  |  |  |  |  |  |  |  |  |  |  |
| PBO to UPA  ADA continued  ADA to UPA  UPA continued  UPA to ADA | 108/613 (17.6)  77/147 (52.4)  22/157 (14.0)  232/367 (63.2)  34/246 (13.8) | 264/600 (44.0)  122/137 (89.1)  31/154 (20.1)  334/359 (93.0)  36/248 (14.5) | 383/571 (67.1)  111/131 (84.7)  82/143 (57.3)  301/343 (87.8)  102/229 (44.5) | 387/553 (70.0)  111/130 (85.4)  82/139 (59.0)  303/338 (89.6)  115/217 (53.0) | 401/539 (74.4)  108/125 (86.4)  84/132 (63.6)  297/328 (90.5)  107/204 (52.5) | 400/532 (75.2)  102/127 (80.3)  82/129 (63.6)  294/326 (90.2)  123/200 (61.5) | 394/515 (76.5)  100/123 (81.3)  81/127 (63.8)  288/322 (89.4)  110/190 (57.9) | 399/506 (78.9)  105/120 (87.5)  81/124 (65.3)  279/310 (90.0)  113/183 (61.7) | 411/495 (83.0)  103/116 (88.8)  88/125 (70.4)  282/309 (91.3)  109/177 (61.6) | 396/488 (81.1)  104/114 (91.2)  95/125 (76.0)  276/308 (89.6)  113/180 (62.8) | 372/456 (81.6)  100/114 (87.7)  85/115 (73.9)  271/290 (93.4)  112/176 (63.6) |
| **CDAI ≤2.8** |  |  |  |  |  |  |  |  |  |  |  |
| PBO to UPA  ADA continued  ADA to UPA  UPA continued  UPA to ADA | 20/613 (3.3)  24/147 (16.3)  1/157 (0.6)  87/367 (23.7)  0/246 (0) | 71/600 (11.8)  45/137 (32.8)  6/154 (3.9)  150/359 (41.8)  4/248 (1.6) | 160/571 (28.0)  55/131 (42.0)  26/143 (18.2)  167/343 (48.7)  13/229 (5.7) | 170/553 (30.7)  51/130 (39.2)  33/139 (23.7)  161/338 (47.6)  22/217 (10.1) | 191/539 (35.4)  55/125 (44.0)  27/132 (20.5)  184/328 (56.1)  26/204 (12.7) | 197/532 (37.0)  56/127 (44.1)  28/129 (21.7)  177/326 (54.3)  32/200 (16.0) | 203/515 (39.4)  53/123 (43.1)  25/127 (19.7)  177/322 (55.0)  32/190 (16.8) | 191/506 (37.7)  54/120 (45.0)  35/124 (28.2)  164/310 (52.9)  31/183 (16.9) | 203/495 (41.0)  54/116 (46.6)  38/125 (30.4)  170/309 (55.0)  31/177 (17.5) | 208/488 (42.6)  56/114 (49.1)  32/125 (25.6)  172/308 (55.8)  31/180 (17.2) | 191/456 (41.9)  62/114 (54.4)  35/115 (30.4)  160/290 (55.2)  34/176 (19.3) |
| **DAS28(CRP) ≤3.2** | |  |  |  |  |  |  |  |  |  |  |
| PBO to UPA  ADA continued  ADA to UPA  UPA continued  UPA to ADA | 92/594 (15.5)  75/144 (52.1)  19/153 (12.4)  251/355 (70.7)  44/233 (18.9) | 250/604 (41.4)  117/136 (86.0)  45/157 (28.7)  328/360 (91.1)  53/248 (21.4) | 402/577 (69.7)  108/134 (80.6)  85/145 (58.6)  299/346 (86.4)  93/235 (39.6) | 402/561 (71.7)  103/130 (79.2)  81/139 (58.3)  288/334 (86.2)  100/217 (46.1) | 409/548 (74.6)  99/128 (77.3)  85/134 (63.4)  296/329 (90.0)  98/204 (48.0) | 408/527 (77.4)  96/127 (75.6)  85/131 (64.9)  286/324 (88.3)  116/199 (58.3) | 392/509 (77.0)  95/122 (77.9)  84/128 (65.6)  285/320 (89.1)  103/188 (54.8) | 394/500 (78.8)  108/123 (87.8)  77/120 (64.2)  288/314 (91.7)  107/182 (58.8) | 399/497 (80.3)  99/120 (82.5)  89/124 (71.8)  281/312 (90.1)  112/181 (61.9) | 383/473 (81.0)  96/115 (83.5)  87/116 (75.0)  274/308 (89.0)  108/177 (61.0) | 354/431 (82.1)  93/112 (83.0)  77/107 (72.0)  258/281 (91.8)  108/163 (66.3) |
| **DAS28(CRP) <2.6** | |  |  |  |  |  |  |  |  |  |  |
| PBO to UPA  ADA continued  ADA to UPA  UPA continued  UPA to ADA | 40/594 (6.7)  49/144 (34.0)  10/153 (6.5)  169/355 (47.6)  19/233 (8.2) | 145/604 (24.0)  85/136 (62.5)  23/157 (14.6)  260/360 (72.2)  20/248 (8.1) | 305/577 (52.9)  90/134 (67.2)  53/145 (36.6)  245/346 (70.8)  48/235 (20.4) | 319/561 (56.9)  81/130 (62.3)  55/139 (39.6)  249/334 (74.6)  57/217 (26.3) | 336/548 (61.3)  86/128 (67.2)  55/134 (41.0)  262/329 (79.6)  66/204 (32.4) | 317/527 (60.2)  86/127 (67.7)  57/131 (43.5)  257/324 (79.3)  73/199 (36.7) | 313/509 (61.5)  78/122 (63.9)  56/128 (43.8)  243/320 (75.9)  72/188 (38.3) | 319/500 (63.8)  87/123 (70.7)  57/120 (47.5)  240/314 (76.4)  69/182 (37.9) | 322/497 (64.8)  82/120 (68.3)  66/124 (53.2)  246/312 (78.8)  73/181 (40.3) | 299/473 (63.2)  80/115 (69.6)  63/116 (54.3)  239/308 (77.6)  71/177 (40.1) | 286/431 (66.4)  78/112 (69.6)  58/107 (54.2)  219/281 (77.9)  79/163 (48.5) |
| **Weeks** | | | | | | | | | | | |
| **n/N (%)** | **156** | **168** | **180** | **192** | **204** | **216** | **228** | **240** | **252** | **264** | **276** |
| **CDAI ≤10** |  |  |  |  |  |  |  |  |  |  |  |
| PBO to UPA  ADA continued  ADA to UPA  UPA continued  UPA to ADA | 357/440 (81.1)  100/109 (91.7)  84/114 (73.7)  260/281 (92.5)  101/157 (64.3) | 367/444 (82.7)  99/110 (90.0)  87/111 (78.4)  266/287 (92.7)  101/159 (63.5) | 364/434 (83.9)  99/110 (90.0)  78/109 (71.6)  263/287 (91.6)  103/151 (68.2) | 359/436 (82.3)  98/109 (89.9)  82/110 (74.5)  256/283 (90.5)  100/153 (65.4) | 360/439 (82.0)  97/108 (89.8)  83/110 (75.5)  246/278 (88.5)  102/147 (69.4) | 361/431 (83.8)  91/105 (86.7)  86/108 (79.6)  258/277 (93.1)  98/141 (69.5) | 358/432 (82.9)  94/103 (91.3)  79/106 (74.5)  248/275 (90.2)  101/144 (70.1) | 358/422 (84.8)  97/104 (93.3)  79/101 (78.2)  250/266 (94.0)  96/142 (67.6) | 338/414 (81.6)  89/95 (93.7)  78/102 (76.5)  241/263 (91.6)  100/136 (73.5) | 333/417 (79.9)  92/98 (93.9)  82/100 (82.0)  244/260 (93.8)  103/137 (75.2) | 342/410 (83.4)  93/99 (93.9)  81/99 (81.8)  243/260 (93.5)  96/141 (68.1) |
| **CDAI ≤2.8** |  |  |  |  |  |  |  |  |  |  |  |
| PBO to UPA  ADA continued  ADA to UPA  UPA continued  UPA to ADA | 203/440 (46.1)  57/109 (52.3)  34/114 (29.8)  159/281 (56.6)  36/157 (22.9) | 199/444 (44.8)  62/110 (56.4)  37/111 (33.3)  164/287 (57.1)  33/159 (20.8) | 186/434 (42.9)  59/110 (53.6)  35/109 (32.1)  166/287 (57.8)  37/151 (24.5) | 185/436 (42.4)  58/109 (53.2)  34/110 (30.9)  171/283 (60.4)  39/153 (25.5) | 198/439 (45.1)  59/108 (54.6)  32/110 (29.1)  167/278 (60.1)  30/147 (20.4) | 189/431 (43.9)  60/105 (57.1)  33/108 (30.6)  167/277 (60.3)  36/141 (25.5) | 186/432 (43.1)  62/103 (60.2)  34/106 (32.1)  171/275 (62.2)  34/144 (23.6) | 194/422 (46.0)  68/104 (65.4)  38/101 (37.6)  158/266 (59.4)  38/142 (26.8) | 166/414 (40.1)  61/95 (64.2)  31/102 (30.4)  160/263 (60.8)  38/136 (27.9) | 181/417 (43.4)  64/98 (65.3)  32/100 (32.0)  162/260 (62.3)  36/137 (26.3) | 184/410 (44.9)  58/99 (58.6)  28/99 (28.3)  168/260 (64.6)  37/141 (26.2) |
| **DAS28(CRP) ≤3.2** | |  |  |  |  |  |  |  |  |  |  |
| PBO to UPA  ADA continued  ADA to UPA  UPA continued  UPA to ADA | 331/405 (81.7)  89/100 (89.0)  78/106 (73.6)  247/265 (93.2)  92/152 (60.5) | 323/401 (80.5)  82/96 (85.4)  79/99 (79.8)  255/269 (94.8)  90/148 (60.8) | 320/399 (80.2)  82/95 (86.3)  68/95 (71.6)  241/260 (92.7)  95/145 (65.5) | 341/414 (82.4)  89/103 (86.4)  79/105 (75.2)  244/264 (92.4)  96/145 (66.2) | 329/409 (80.4)  85/98 (86.7)  73/101 (72.3)  232/259 (89.6)  89/135 (65.9) | 326/394 (82.7)  77/94 (81.9)  79/100 (79.0)  238/258 (92.2)  92/134 (68.7) | 336/413 (81.4)  82/92 (89.1)  76/102 (74.5)  242/268 (90.3)  99/139 (71.2) | 338/402 (84.1)  88/97 (90.7)  70/96 (72.9)  232/252 (92.1)  94/138 (68.1) | 310/384 (80.7)  79/89 (88.8)  73/94 (77.7)  229/253 (90.5)  91/130 (70.0) | 309/392 (78.8)  86/96 (89.6)  82/94 (87.2)  236/252 (93.7)  96/131 (73.3) | 316/387 (81.7)  86/96 (89.6)  76/90 (84.4)  230/247 (93.1)  85/129 (65.9) |
| **DAS28(CRP) <2.6** | |  |  |  |  |  |  |  |  |  |  |
| PBO to UPA  ADA continued  ADA to UPA  UPA continued  UPA to ADA | 267/405 (65.9)  73/100 (73.0)  60/106 (56.6)  216/265 (81.5)  68/152 (44.7) | 259/401 (64.6)  76/96 (79.2)  58/99 (58.6)  224/269 (83.3)  62/148 (41.9) | 263/399 (65.9)  66/95 (69.5)  57/95 (60.0)  217/260 (83.5)  71/145 (49.0) | 277/414 (66.9)  73/103 (70.9)  55/105 (52.4)  213/264 (80.7)  74/145 (51.0) | 272/409 (66.5)  68/98 (69.4)  56/101 (55.4)  213/259 (82.2)  65/135 (48.1) | 271/394 (68.8)  66/94 (70.2)  56/100 (56.0)  216/258 (83.7)  64/134 (47.8) | 280/413 (67.8)  68/92 (73.9)  61/102 (59.8)  214/268 (79.9)  66/139 (47.5) | 282/402 (70.1)  79/97 (81.4)  57/96 (59.4)  200/252 (79.4)  66/138 (47.8) | 255/384 (66.4)  69/89 (77.5)  52/94 (55.3)  202/253 (79.8)  64/130 (49.2) | 263/392 (67.1)  81/96 (84.4)  54/94 (57.4)  214/252 (84.9)  68/131 (51.9) | 267/387 (69.0)  79/96 (82.3)  60/90 (66.7)  207/247 (83.8)  69/129 (53.5) |
| **Weeks** | | | | | | | | | | | |
| **n/N (%)** | **288** | **300** | **312** | **324** | **336** | **348** | **360** | **372** |  |  |  |
| **CDAI ≤10** |  |  |  |  |  |  |  |  |  |  |  |
| PBO to UPA  ADA continued  ADA to UPA  UPA continued  UPA to ADA | 327/404 (80.9)  85/94 (90.4)  75/96 (78.1)  234/257 (91.1)  94/133 (70.7) | 324/389 (83.3)  90/96 (93.8)  74/99 (74.7)  231/254 (90.9)  92/125 (73.6) | 313/378 (82.8)  87/94 (92.6)  74/97 (76.3)  228/246 (92.7)  93/131 (71.0) | 309/375 (82.4)  85/92 (92.4)  73/97 (75.3)  228/250 (91.2)  82/123 (66.7) | 301/363 (82.9)  85/91 (93.4)  74/96 (77.1)  222/242 (91.7)  89/123 (72.4) | 284/351 (80.9)  85/91 (93.4)  72/98 (73.5)  219/237 (92.4)  85/117 (72.6) | 287/352 (81.5)  81/87 (93.1)  72/96 (75.0)  210/231 (90.9)  92/117 (78.6) | 283/347 (81.6)  81/86 (94.2)  73/90 (81.1)  212/230 (92.2)  78/111 (70.3) |  |  |  |
| **CDAI ≤2.8** |  |  |  |  |  |  |  |  |  |  |  |
| PBO to UPA  ADA continued  ADA to UPA  UPA continued  UPA to ADA | 168/404 (41.6)  59/94 (62.8)  29/96 (30.2)  145/257 (56.4)  38/133 (28.6) | 172/389 (44.2)  60/96 (62.5)  29/99 (29.3)  154/254 (60.6)  32/125 (25.6) | 171/378 (45.2)  58/94 (61.7)  34/97 (35.1)  140/246 (56.9)  35/131 (26.7) | 179/375 (47.7)  54/92 (58.7)  26/97 (26.8)  157/250 (62.8)  34/123 (27.6) | 157/363 (43.3)  60/91 (65.9)  32/96 (33.3)  140/242 (57.9)  27/123 (22.0) | 158/351 (45.0)  55/91 (60.4)  31/98 (31.6)  152/237 (64.1)  33/117 (28.2) | 168/352 (47.7)  47/87 (54.0)  28/96 (29.2)  139/231 (60.2)  27/117 (23.1) | 159/347 (45.8)  46/86 (53.5)  32/90 (35.6)  145/230 (63.0)  31/111 (27.9) |  |  |  |
| **DAS28(CRP) ≤3.2** | |  |  |  |  |  |  |  |  |  |  |
| PBO to UPA  ADA continued  ADA to UPA  UPA continued  UPA to ADA | 310/376 (82.4)  82/95 (86.3)  79/98 (80.6)  225/250 (90.0)  92/128 (71.9) | 312/375 (83.2)  83/90 (92.2)  71/96 (74.0)  221/244 (90.6)  83/118 (70.3) | 294/360 (81.7)  85/94 (90.4)  74/96 (77.1)  216/239 (90.4)  87/126 (69.0) | 289/353 (81.9)  79/92 (85.9)  70/93 (75.3)  215/240 (89.6)  76/117 (65.0) | 273/335 (81.5)  82/92 (89.1)  74/94 (78.7)  213/230 (92.6)  80/114 (70.2) | 262/324 (80.9)  82/88 (93.2)  65/90 (72.2)  214/231 (92.6)  83/111 (74.8) | 264/318 (83.0)  74/84 (88.1)  67/90 (74.4)  204/219 (93.2)  77/107 (72.0) | 251/314 (79.9)  72/81 (88.9)  70/87 (80.5)  196/214 (91.6)  67/103 (65.0) |  |  |  |
| **DAS28(CRP) <2.6** | |  |  |  |  |  |  |  |  |  |  |
| PBO to UPA  ADA continued  ADA to UPA  UPA continued  UPA to ADA | 257/376 (68.4)  75/95 (78.9)  63/98 (64.3)  191/250 (76.4)  63/128 (49.2) | 262/375 (69.9)  74/90 (82.2)  58/96 (60.4)  195/244 (79.9)  55/118 (46.6) | 243/360 (67.5)  75/94 (79.8)  65/96 (67.7)  192/239 (80.3)  65/126 (51.6) | 244/353 (69.1)  72/92 (78.3)  57/93 (61.3)  183/240 (76.3)  54/117 (46.2) | 222/335 (66.3)  75/92 (81.5)  55/94 (58.5)  192/230 (83.5)  60/114 (52.6) | 220/324 (67.9)  72/88 (81.8)  52/90 (57.8)  191/231 (82.7)  56/111 (50.5) | 218/318 (68.6)  62/84 (73.8)  57/90 (63.3)  182/219 (83.1)  54/107 (50.5) | 204/314 (65.0)  59/81 (72.8)  57/87 (65.5)  178/214 (83.2)  56/103 (54.4) |  |  |  |

ADA, adalimumab; AO, as observed; CDAI, Clinical Disease Activity Index; DAS28(CRP), 28-joint Disease Activity Score based on C-reactive protein; LDA, low disease activity; PBO, placebo; UPA, upadacitinib.

**Table S8**  Proportions of patients achieving ACR20, ACR50 and ACR70 through 372 weeks (AO)

| **Weeks** | | | | | | | | | | | | |
| --- | --- | --- | --- | --- | --- | --- | --- | --- | --- | --- | --- | --- |
| **n/N (%)** | **12** | **26** | **48** | **60** | **72** | **84** | **96** | **108** | **120** | **132** | **144** |  |
| **ACR20** |  |  |  |  |  |  |  |  |  |  |  |  |
| PBO to UPA  ADA continued  ADA to UPA  UPA continued  UPA to ADA | 236/616 (38.3)  128/155 (82.6)  81/155 (52.3)  321/364 (88.2)  131/243 (53.9) | 447/598 (74.7)  131/136 (96.3)  111/155 (71.6)  343/357 (96.1)  152/242 (62.8) | 502/577 (87.0)  119/133 (89.5)  117/143 (81.8)  330/344 (95.9)  169/229 (73.8) | 481/564 (85.3)  122/130 (93.8)  119/136 (87.5)  319/337 (94.7)  173/218 (79.4) | 481/548 (87.8)  114/129 (88.4)  121/134 (90.3)  308/327 (94.2)  161/202 (79.7) | 473/537 (88.1)  112/128 (87.5)  112/129 (86.8)  312/325 (96.0)  162/200 (81.0) | 471/518 (90.9)  113/123 (91.9)  121/129 (93.8)  308/323 (95.4)  158/191 (82.7) | 459/512 (89.6)  113/122 (92.6)  114/127 (89.8)  304/314 (96.8)  143/185 (77.3) | 464/501 (92.6)  110/119 (92.4)  110/124 (88.7)  303/314 (96.5)  150/183 (82.0) | 447/484 (92.4)  109/118 (92.4)  107/120 (89.2)  292/309 (94.5)  147/180 (81.7) | 418/459 (91.1)  106/116 (91.4)  103/114 (90.4)  277/292 (94.9)  148/174 (85.1) |  |
| **ACR50** |  |  |  |  |  |  |  |  |  |  |  |  |
| PBO to UPA  ADA continued  ADA to UPA  UPA continued  UPA to ADA | 97/619 (15.7)  75/152 (49.3)  19/157 (12.1)  241/367 (65.7)  49/246 (19.9) | 272/601 (45.3)  109/136 (80.1)  60/154 (39.0)  311/356 (87.4)  66/243 (27.2) | 373/576 (64.8)  103/132 (78.0)  83/141 (58.9)  285/342 (83.3)  96/232 (41.4) | 374/563 (66.4)  103/132 (78.0)  87/137 (63.5)  281/332 (84.6)  111/216 (51.4) | 387/546 (70.9)  96/129 (74.4)  94/134 (70.1)  281/329 (85.4)  97/202 (48.0) | 381/529 (72.0)  99/128 (77.3)  85/130 (65.4)  285/325 (87.7)  107/200 (53.5) | 374/518 (72.2)  97/125 (77.6)  87/128 (68.0)  277/321 (86.3)  98/189 (51.9) | 378/507 (74.6)  97/123 (78.9)  81/125 (64.8)  264/312 (84.6)  107/179 (59.8) | 372/495 (75.2)  92/120 (76.7)  91/123 (74.0)  275/314 (87.6)  106/181 (58.6) | 361/481 (75.1)  96/118 (81.4)  80/119 (67.2)  256/305 (83.9)  99/178 (55.6) | 346/456 (75.9)  93/115 (80.9)  79/112 (70.5)  242/292 (82.9)  105/171 (61.4) |  |
| **ACR70** |  |  |  |  |  |  |  |  |  |  |  |  |
| PBO to UPA  ADA continued  ADA to UPA  UPA continued  UPA to ADA | 32/624 (5.1)  38/151 (25.2)  5/158 (3.2)  152/371 (41.0)  11/250 (4.4) | 121/604 (20.0)  73/135 (54.1)  15/154 (9.7)  215/354 (60.7)  16/245 (6.5) | 246/576 (42.7)  75/131 (57.3)  52/142 (36.6)  226/342 (66.1)  43/233 (18.5) | 256/561 (45.6)  71/131 (54.2)  50/139 (36.0)  227/336 (67.6)  63/220 (28.6) | 284/547 (51.9)  77/129 (59.7)  56/134 (41.8)  228/328 (69.5)  53/204 (26.0) | 273/537 (50.8)  75/124 (60.5)  58/129 (45.0)  224/324 (69.1)  60/199 (30.2) | 293/517 (56.7)  70/123 (56.9)  56/126 (44.4)  223/324 (68.8)  65/191 (34.0) | 271/504 (53.8)  75/122 (61.5)  50/123 (40.7)  221/312 (70.8)  60/182 (33.0) | 278/495 (56.2)  72/120 (60.0)  60/124 (48.4)  218/314 (69.4)  66/182 (36.3) | 276/483 (57.1)  74/117 (63.2)  56/119 (47.1)  211/306 (69.0)  58/180 (32.2) | 259/453 (57.2)  73/115 (63.5)  54/111 (48.6)  210/290 (72.4)  60/173 (34.7) |  |
| **Weeks** | | | | | | | | | | | | |
| **n/N (%)** | **156** | **168** | **180** | **192** | **204** | **216** | **228** | **240** | **252** | **264** | **276** |  |
| **ACR20** |  |  |  |  |  |  |  |  |  |  |  |  |
| PBO to UPA  ADA continued  ADA to UPA  UPA continued  UPA to ADA | 404/442 (91.4)  102/109 (93.6)  101/115 (87.8)  272/283 (96.1)  136/163 (83.4) | 394/445 (88.5)  105/111 (94.6)  101/109 (92.7)  278/287 (96.9)  127/159 (79.9) | 401/438 (91.6)  105/110 (95.5)  95/104 (91.3)  276/285 (96.8)  136/154 (88.3) | 404/446 (90.6)  107/113 (94.7)  99/111 (89.2)  272/283 (96.1)  133/152 (87.5) | 400/441 (90.7)  102/109 (93.6)  97/109 (89.0)  270/278 (97.1)  123/145 (84.8) | 393/430 (91.4)  100/104 (96.2)  99/107 (92.5)  266/276 (96.4)  124/144 (86.1) | 395/436 (90.6)  100/104 (96.2)  96/107 (89.7)  266/279 (95.3)  117/148 (79.1) | 389/426 (91.3)  102/105 (97.1)  95/102 (93.1)  264/271 (97.4)  122/147 (83.0) | 377/418 (90.2)  94/98 (95.9)  90/99 (90.9)  254/266 (95.5)  117/138 (84.8) | 376/416 (90.4)  97/101 (96.0)  89/99 (89.9)  257/265 (97.0)  121/139 (87.1) | 372/412 (90.3)  95/100 (95.0)  91/98 (92.9)  251/257 (97.7)  120/140 (85.7) |  |
| **ACR50** |  |  |  |  |  |  |  |  |  |  |  |  |
| PBO to UPA  ADA continued  ADA to UPA  UPA continued  UPA to ADA | 326/434 (75.1)  91/111 (82.0)  85/114 (74.6)  250/284 (88.0)  93/159 (58.5) | 333/436 (76.4)  88/110 (80.0)  83/107 (77.6)  254/286 (88.8)  92/159 (59.7) | 331/432 (76.6)  95/110 (86.4)  76/104 (73.1)  254/284 (89.4)  99/155 (63.9) | 332/440 (75.5)  95/113 (84.1)  76/110 (69.1)  253/285 (88.8)  91/151 (60.3) | 324/430 (75.3)  91/109 (83.5)  75/107 (70.1)  244/280 (87.1)  90/145 (62.1) | 330/428 (77.1)  86/104 (82.7)  80/105 (76.2)  245/275 (89.1)  84/141 (59.6) | 329/431 (76.3)  93/104 (89.4)  79/105 (75.2)  239/277 (86.3)  84/145 (57.9) | 326/421 (77.4)  93/105 (88.6)  75/100 (75.0)  234/269 (87.0)  85/143 (59.4) | 306/415 (73.7)  87/99 (87.9)  71/99 (71.7)  236/268 (88.1)  93/136 (68.4) | 303/412 (73.5)  87/101 (86.1)  71/98 (72.4)  235/263 (89.4)  88/139 (63.3) | 306/407 (75.2)  89/100 (89.0)  67/95 (70.5)  227/257 (88.3)  84/137 (61.3) |  |
| **ACR70** |  |  |  |  |  |  |  |  |  |  |  |  |
| PBO to UPA  ADA continued  ADA to UPA  UPA continued  UPA to ADA | 257/436 (58.9)  71/107 (66.4)  60/113 (53.1)  211/285 (74.0)  54/162 (33.3) | 246/439 (56.0)  73/109 (67.0)  53/109 (48.6)  205/287 (71.4)  55/156 (35.3) | 254/430 (59.1)  74/108 (68.5)  57/106 (53.8)  213/284 (75.0)  61/152 (40.1) | 264/442 (59.7)  75/111 (67.6)  56/109 (51.4)  211/284 (74.3)  59/153 (38.6) | 245/429 (57.1)  74/107 (69.2)  53/107 (49.5)  201/278 (72.3)  61/145 (42.1) | 252/424 (59.4)  67/104 (64.4)  54/104 (51.9)  203/277 (73.3)  56/141 (39.7) | 251/429 (58.5)  76/102 (74.5)  51/101 (50.5)  199/279 (71.3)  53/146 (36.3) | 258/424 (60.8)  76/103 (73.8)  50/98 (51.0)  201/272 (73.9)  60/142 (42.3) | 236/413 (57.1)  70/97 (72.2)  47/99 (47.5)  194/269 (72.1)  50/133 (37.6) | 241/410 (58.8)  76/99 (76.8)  50/98 (51.0)  190/260 (73.1)  60/137 (43.8) | 245/408 (60.0)  70/98 (71.4)  51/97 (52.6)  187/257 (72.8)  56/139 (40.3) |  |
| **Weeks** | | | | | | | | | | | | |
| **n/N (%)** | **288** | **300** | **312** | **324** | **336** | **348** | **360** | **372** |  |  |  |  |
| **ACR20** |  |  |  |  |  |  |  |  |  |  |  |  |
| PBO to UPA  ADA continued  ADA to UPA  UPA continued  UPA to ADA | 360/400 (90.0)  92/96 (95.8)  89/99 (89.9)  246/260 (94.6)  115/136 (84.6) | 355/394 (90.1)  92/96 (95.8)  82/97 (84.5)  246/253 (97.2)  116/129 (89.9) | 349/381 (91.6)  92/95 (96.8)  86/97 (88.7)  239/252 (94.8)  113/132 (85.6) | 344/374 (92.0)  90/95 (94.7)  82/96 (85.4)  238/250 (95.2)  103/123 (83.7) | 335/365 (91.8)  91/93 (97.8)  84/95 (88.4)  232/243 (95.5)  100/122 (82.0) | 320/350 (91.4)  89/92 (96.7)  84/96 (87.5)  228/240 (95.0)  100/120 (83.3) | 319/351 (90.9)  88/90 (97.8)  86/96 (89.6)  222/232 (95.7)  100/115 (87.0) | 318/351 (90.6)  86/87 (98.9)  79/91 (86.8)  218/229 (95.2)  99/111 (89.2) |  |  |  |  |
| **ACR50** |  |  |  |  |  |  |  |  |  |  |  |  |
| PBO to UPA  ADA continued  ADA to UPA  UPA continued  UPA to ADA | 296/397 (74.6)  84/96 (87.5)  66/97 (68.0)  220/261 (84.3)  85/135 (63.0) | 303/390 (77.7)  87/96 (90.6)  67/97 (69.1)  222/253 (87.7)  84/129 (65.1) | 285/374 (76.2)  82/94 (87.2)  69/95 (72.6)  219/251 (87.3)  77/129 (57.9) | 283/374 (75.7)  78/94 (83.0)  66/94 (70.2)  213/249 (85.5)  74/123 (60.2) | 275/362 (76.0)  87/94 (92.6)  65/93 (69.9)  208/239 (87.0)  68/121 (56.2) | 263/351 (74.9)  81/93 (87.1)  65/96 (67.7)  206/239 (86.2)  71/119 (59.7) | 268/350 (76.6)  77/90 (85.6)  62/94 (66.0)  196/234 (83.8)  72/115 (62.6) | 270/345 (78.3)  79/87 (90.8)  66/90 (73.3)  202/228 (88.6)  71/111 (64.0) |  |  |  |  |
| **ACR70** |  |  |  |  |  |  |  |  |  |  |  |  |
| PBO to UPA  ADA continued  ADA to UPA  UPA continued  UPA to ADA | 223/399 (55.9)  63/95 (66.3)  41/96 (42.7)  179/256 (69.9)  55/134 (41.0) | 233/389 (59.9)  65/92 (70.7)  43/96 (44.8)  183/254 (72.0)  51/128 (39.8) | 230/374 (61.5)  61/93 (65.6)  49/96 (51.0)  184/253 (72.7)  56/131 (42.7) | 228/371 (61.5)  59/91 (64.8)  44/93 (47.3)  176/250 (70.4)  45/123 (36.6) | 219/363 (60.3)  64/91 (70.3)  44/93 (47.3)  172/237 (72.6)  50/121 (41.3) | 212/349 (60.7)  65/92 (70.7)  43/95 (45.3)  170/239 (71.1)  48/116 (41.4) | 213/346 (61.6)  62/89 (69.7)  42/94 (44.7)  163/235 (69.4)  52/114 (45.6) | 205/344 (59.6)  59/86 (68.6)  43/91 (47.3)  172/228 (75.4)  45/111 (40.5) |  |  |  |  |

ACR20/50/70, ≥20/50/70% improvement in American College of Rheumatology response criteria; ADA, adalimumab; AO, as observed; LDA, low disease activity; PBO, placebo; UPA, upadacitinib.

**Table S9**  Proportions of non-responders or incomplete responders who achieved CDAI LDA (≤10), CDAI remission (≤2.8), DAS28(CRP) ≤3.2 and DAS28(CRP) ≤2.6 through 336 weeks post-switch (AO)

| **Weeks post-switch** | | | | | | | | | | | | |
| --- | --- | --- | --- | --- | --- | --- | --- | --- | --- | --- | --- | --- |
| **n/N (%)** | **Switch** | **12** | **24** | **36** | **48** | **60** | **72** | **84** | **96** | **108** | **120** |  |
| **CDAI ≤10**  UPA to ADA – NR  UPA to ADA – IR  ADA to UPA – NR  ADA to UPA – IR | 9/124 (7.3)  9/126 (7.1)  7/74 (9.5)  5/82 (6.1) | 33/120 (27.5)  41/122 (33.6)  24/72 (33.3)  34/76 (44.7) | 41/114 (36.0)  54/120 (45.0)  33/70 (47.1)  44/76 (57.9) | 51/107 (47.7)  58/112 (51.8)  36/67 (53.7)  45/73 (61.6) | 53/104 (51.0)  53/103 (51.5)  38/66 (57.6)  43/70 (61.4) | 57/102 (55.9)  64/102 (62.7)  41/62 (66.1)  43/66 (65.2) | 55/96 (57.3)  62/97 (63.9)  39/62 (62.9)  39/68 (57.4) | 49/92 (53.3)  60/93 (64.5)  43/60 (71.7)  41/65 (63.1) | 53/88 (60.2)  57/93 (61.3)  39/59 (66.1)  47/64 (73.4) | 54/86 (62.8)  59/90 (65.6)  42/61 (68.9)  49/64 (76.6) | 52/90 (57.8)  64/90 (71.1)  45/61 (73.8)  44/58 (75.9) |  |
| **CDAI ≤2.8**  UPA to ADA – NR  UPA to ADA – IR  ADA to UPA – NR  ADA to UPA – IR | 1/124 (0.8)  0/126 (0.0)  1/74 (1.4)  0/82 (0.0) | 5/120 (4.2)  3/122 (2.5)  6/72 (8.3)  7/76 (9.2) | 6/114 (5.3)  6/120 (5.0)  10/70 (14.3)  12/76 (15.8) | 6/107 (5.6)  10/112 (8.9)  14/67 (20.9)  19/73 (26.0) | 12/104 (11.5)  12/103 (11.7)  14/66 (21.2)  18/70 (25.7) | 15/102 (14.7)  13/102 (12.7)  9/62 (14.5)  17/66 (25.8) | 17/96 (17.7)  15/97 (15.5)  10/62 (16.1)  10/68 (14.7) | 18/92 (19.6)  16/93 (17.2)  16/60 (26.7)  20/65 (30.8) | 14/88 (15.9)  15/93 (16.1)  15/59 (25.4)  21/64 (32.8) | 18/86 (20.9)  11/90 (12.2)  17/61 (27.9)  14/64 (21.9) | 18/90 (20.0)  16/90 (17.8)  18/61 (29.5)  18/58 (31.0) |  |
| **DAS28(CRP)** **≤3.2**  UPA to ADA – NR  UPA to ADA – IR  ADA to UPA – NR  ADA to UPA – IR | 14/121 (11.6)  28/126 (22.2)  8/74 (10.8)  10/82 (12.2) | 30/119 (25.2)  41/114 (36.0)  31/74 (41.9)  46/76 (60.5) | 38/109 (34.9)  53/121 (43.8)  38/70 (54.3)  44/77 (57.1) | 41/113 (36.3)  51/112 (45.5)  40/68 (58.8)  45/72 (62.5) | 48/104 (46.2)  46/101 (45.5)  37/67 (55.2)  45/68 (66.2) | 51/103 (49.5)  59/102 (57.8)  40/66 (60.6)  45/67 (67.2) | 57/95 (60.0)  57/97 (58.8)  40/64 (62.5)  39/67 (58.2) | 45/90 (50.0)  56/93 (60.2)  47/61 (77.0)  35/59 (59.3) | 54/89 (60.7)  58/91 (63.7)  40/60 (66.7)  47/64 (73.4) | 53/90 (58.9)  56/89 (62.9)  42/61 (68.9)  47/60 (78.3) | 51/87 (58.6)  57/81 (70.4)  40/56 (71.4)  43/55 (78.2) |  |
| **DAS28(CRP)** **<2.6**  UPA to ADA – NR  UPA to ADA – IR  ADA to UPA – NR  ADA to UPA – IR | 5/121 (4.1)  6/126 (4.8)  3/74 (4.1)  3/82 (3.7) | 15/119 (12.6)  19/114 (16.7)  19/74 (25.7)  26/76 (34.2) | 21/109 (19.3)  28/121 (23.1)  22/70 (31.4)  29/77 (37.7) | 22/113 (19.5)  29/112 (25.9)  23/68 (33.8)  34/72 (47.2) | 26/104 (25.0)  33/101 (32.7)  21/67 (31.3)  30/68 (44.1) | 34/103 (33.0)  35/102 (34.3)  25/66 (37.9)  29/67 (43.3) | 37/95 (38.9)  41/97 (42.3)  28/64 (43.8)  25/67 (37.3) | 30/90 (33.3)  39/93 (41.9)  32/61 (52.5)  25/59 (42.4) | 30/89 (33.7)  41/91 (45.1)  31/60 (51.7)  33/64 (51.6) | 33/90 (36.7)  35/89 (39.3)  34/61 (55.7)  30/60 (50.0) | 36/87 (41.4)  43/81 (53.1)  32/56 (57.1)  28/55 (50.9) |  |
| **Weeks post-switch** | | | | | | | | | | | | |
| **n/N (%)** | **132** | **144** | **156** | **168** | **180** | **192** | **204** | **216** | **228** | **240** | **252** |  |
| **CDAI ≤10**  UPA to ADA – NR  UPA to ADA – IR  ADA to UPA – NR  ADA to UPA – IR | 47/83 (56.6)  52/85 (61.2)  41/57 (71.9)  45/57 (78.9) | 49/71 (69.0)  50/83 (60.2)  41/56 (73.2)  43/59 (72.9) | 52/77 (67.5)  56/77 (72.7)  43/52 (82.7)  39/56 (69.6) | 46/74 (62.2)  51/77 (66.2)  39/54 (72.2)  44/56 (78.6) | 50/76 (65.8)  55/75 (73.3)  36/53 (67.9)  38/56 (67.9) | 46/72 (63.9)  49/70 (70.0)  46/54 (85.2)  41/53 (77.4) | 51/71 (71.8)  49/73 (67.1)  46/55 (83.6)  37/53 (69.8) | 49/70 (70.0)  46/72 (63.9)  43/52 (82.7)  38/49 (77.6) | 51/70 (72.9)  48/66 (72.7)  40/52 (76.9)  37/51 (72.5) | 39/56 (69.6)  53/70 (75.7)  34/44 (77.3)  41/49 (83.7) | 36/53 (67.9)  51/72 (70.8)  36/44 (81.8)  40/50 (80.0) |  |
| **CDAI ≤2.8**  UPA to ADA – NR  UPA to ADA – IR  ADA to UPA – NR  ADA to UPA – IR | 18/83 (21.7)  20/85 (23.5)  17/57 (29.8)  16/57 (28.1) | 16/71 (22.5)  16/83 (19.3)  18/56 (32.1)  19/59 (32.2) | 18/77 (23.4)  22/77 (28.6)  18/52 (34.6)  16/56 (28.6) | 15/74 (20.3)  22/77 (28.6)  19/54 (35.2)  21/56 (37.5) | 16/76 (21.1)  16/75 (21.3)  12/53 (22.6)  16/56 (28.6) | 15/72 (20.8)  21/70 (30.0)  17/54 (31.5)  17/53 (32.1) | 15/71 (21.1)  16/73 (21.9)  16/55 (29.1)  16/53 (30.2) | 18/70 (25.7)  18/72 (25.0)  18/52 (34.6)  21/49 (42.9) | 20/70 (28.6)  18/66 (27.3)  17/52 (32.7)  14/51 (27.5) | 15/56 (26.8)  19/70 (27.1)  13/44 (29.5)  16/49 (32.7) | 14/53 (26.4)  21/72 (29.2)  14/44 (31.8)  16/50 (32.0) |  |
| **DAS28(CRP)** **≤3.2**  UPA to ADA – NR  UPA to ADA – IR  ADA to UPA – NR  ADA to UPA – IR | 49/80 (61.3)  47/78 (60.3)  33/52 (63.5)  42/54 (77.8) | 46/73 (63.0)  47/78 (60.3)  39/51 (76.5)  40/52 (76.9) | 44/71 (62.0)  51/72 (70.8)  37/46 (80.4)  33/46 (71.7) | 42/72 (58.3)  52/74 (70.3)  36/51 (70.6)  44/55 (80.0) | 44/71 (62.0)  47/67 (70.1)  33/49 (67.3)  35/50 (70.0) | 42/68 (61.8)  46/67 (68.7)  39/51 (76.5)  38/50 (76.0) | 48/68 (70.6)  47/70 (67.1)  42/50 (84.0)  37/51 (72.5) | 49/68 (72.1)  45/69 (65.2)  39/49 (79.6)  32/47 (68.1) | 52/70 (74.3)  44/61 (72.1)  38/50 (76.0)  33/45 (73.3) | 35/54 (64.8)  52/69 (75.4)  35/43 (81.4)  39/45 (86.7) | 31/48 (64.6)  46/65 (70.8)  37/43 (86.0)  35/42 (83.3) |  |
| **DAS28(CRP)** **<2.6**  UPA to ADA – NR  UPA to ADA – IR  ADA to UPA – NR  ADA to UPA – IR | 36/80 (45.0)  34/78 (43.6)  30/52 (57.7)  30/54 (55.6) | 32/73 (43.8)  37/78 (47.4)  30/51 (58.8)  30/52 (57.7) | 26/71 (36.6)  41/72 (56.9)  28/46 (60.9)  28/46 (60.9) | 30/72 (41.7)  40/74 (54.1)  29/51 (56.9)  33/55 (60.0) | 34/71 (47.9)  35/67 (52.2)  20/49 (40.8)  27/50 (54.0) | 30/68 (44.1)  33/67 (49.3)  31/51 (60.8)  27/50 (54.0) | 33/68 (48.5)  30/70 (42.9)  30/50 (60.0)  31/51 (60.8) | 34/68 (50.0)  31/69 (44.9)  31/49 (63.3)  27/47 (57.4) | 35/70 (50.0)  32/61 (52.5)  29/50 (58.0)  22/45 (48.9) | 26/54 (48.1)  34/69 (49.3)  24/43 (55.8)  27/45 (60.0) | 24/48 (50.0)  39/65 (60.0)  23/43 (53.5)  28/42 (66.7) |  |
| **Weeks post-switch** | | | | | | | | | | | | |
| **n/N (%)** | **264** | **276** | **288** | **300** | **312** | **324** | **336** |  |  |  |  |  |
| **CDAI ≤10**  UPA to ADA – NR  UPA to ADA – IR  ADA to UPA – NR  ADA to UPA – IR | 35/54 (64.8)  49/66 (74.2)  34/43 (79.1)  39/47 (83.0) | 34/51 (66.7)  49/63 (77.8)  30/41 (73.2)  37/51 (72.5) | 34/50 (68.0)  52/67 (77.6)  31/41 (75.6)  35/50 (70.0) | 35/50 (70.0)  41/63 (65.1)  32/39 (82.1)  39/51 (76.5) | 28/46 (60.9)  48/64 (75.0)  30/40 (75.0)  38/49 (77.6) | 33/46 (71.7)  45/59 (76.3)  29/40 (72.5)  37/51 (72.5) | 30/43 (69.8)  49/61 (80.3)  28/40 (70.0)  39/51 (76.5) |  |  |  |  |  |
| **CDAI ≤2.8**  UPA to ADA – NR  UPA to ADA – IR  ADA to UPA – NR  ADA to UPA – IR | 13/54 (24.1)  20/66 (30.3)  10/43 (23.3)  17/47 (36.2) | 14/51 (27.5)  17/63 (27.0)  10/41 (24.4)  17/51 (33.3) | 11/50 (22.0)  21/67 (31.3)  8/41 (19.5)  17/50 (34.0) | 11/50 (22.0)  20/63 (31.7)  13/39 (33.3)  16/51 (31.4) | 10/46 (21.7)  15/64 (23.4)  8/40 (20.0)  19/49 (38.8) | 11/46 (23.9)  20/59 (33.9)  10/40 (25.0)  20/51 (39.2) | 9/43 (20.9)  14/61 (23.0)  9/40 (22.5)  18/51 (35.3) |  |  |  |  |  |
| **DAS28(CRP) ≤3.2**  UPA to ADA – NR  UPA to ADA – IR  ADA to UPA – NR  ADA to UPA – IR | 28/49 (57.1)  50/64 (78.1)  34/42 (81.0)  40/49 (81.6) | 31/48 (64.6)  45/58 (77.6)  33/41 (80.5)  34/47 (72.3) | 29/49 (59.2)  50/63 (79.4)  30/42 (71.4)  35/49 (71.4) | 31/49 (63.3)  42/62 (67.7)  33/39 (84.6)  36/48 (75.0) | 23/42 (54.8)  45/59 (76.3)  29/38 (76.3)  36/49 (73.5) | 24/41 (58.5)  47/58 (81.0)  31/39 (79.5)  32/47 (68.1) | 27/40 (67.5)  41/57 (71.9)  27/37 (73.0)  33/45 (73.3) |  |  |  |  |  |
| **DAS28(CRP) <2.6**  UPA to ADA – NR  UPA to ADA – IR  ADA to UPA – NR  ADA to UPA – IR | 22/49 (44.9)  31/64 (48.4)  26/42 (61.9)  35/49 (71.4) | 25/48 (52.1)  30/58 (51.7)  25/41 (61.0)  29/47 (61.7) | 18/49 (36.7)  34/63 (54.0)  23/42 (54.8)  32/49 (65.3) | 24/49 (49.0)  31/62 (50.0)  28/39 (71.8)  28/48 (58.3) | 15/42 (35.7)  34/59 (57.6)  24/38 (63.2)  28/49 (57.1) | 18/41 (43.9)  28/58 (48.3)  21/39 (53.8)  25/47 (53.2) | 20/40 (50.0)  30/57 (52.6)  22/37 (59.5)  29/45 (64.4) |  |  |  |  |  |

ADA, adalimumab; AO, as observed; CDAI, Clinical Disease Activity Index; DAS28(CRP), 28-joint Disease Activity Score based on C-reactive protein; IR, incomplete responders; LDA, low disease activity; NR, non-responders; UPA, upadacitinib.

**Table S10**  Proportions of non-responders or incomplete responders who achieved ACR20, ACR50 and ACR70 through 336 weeks post-switch (AO)

| **Weeks post-switch** | | | | | | | | | | | | |
| --- | --- | --- | --- | --- | --- | --- | --- | --- | --- | --- | --- | --- |
| **n/N (%)** | **Switch** | **12** | **24** | **36** | **48** | **60** | **72** | **84** | **96** | **108** | **120** |  |
| **ACR20** |  |  |  |  |  |  |  |  |  |  |  |  |
| UPA to ADA – NR  UPA to ADA – IR  ADA to UPA – NR  ADA to UPA – IR | 3/125 (2.4)  93/122 (76.2)  3/77 (3.9)  60/80 (75.0) | 68/118 (57.6)  94/119 (79.0)  49/75 (65.3)  67/77 (87.0) | 67/113 (59.3)  92/119 (77.3)  53/71 (74.6)  65/75 (86.7) | 79/109 (72.5)  92/110 (83.6)  52/67 (77.6)  63/71 (88.7) | 78/106 (73.6)  90/101 (89.1)  58/66 (87.9)  61/69 (88.4) | 73/102 (71.6)  89/102 (87.3)  58/65 (89.2)  58/65 (89.2) | 71/96 (74.0)  87/98 (88.8)  55/64 (85.9)  63/67 (94.0) | 67/92 (72.8)  82/94 (87.2)  58/62 (93.5)  61/65 (93.8) | 65/91 (71.4)  83/92 (90.2)  52/61 (85.2)  57/63 (90.5) | 65/91 (71.4)  79/90 (87.8)  53/61 (86.9)  57/61 (93.4) | 69/89 (77.5)  78/89 (87.6)  51/60 (85.0)  53/57 (93.0) |  |
| **ACR50** |  |  |  |  |  |  |  |  |  |  |  |  |
| UPA to ADA – NR  UPA to ADA – IR  ADA to UPA – NR  ADA to UPA – IR | 2/125 (1.6)  38/121 (31.4)  2/77 (2.6)  29/80 (36.3) | 33/120 (27.5)  52/118 (44.1)  26/73 (35.6)  50/75 (66.7) | 29/112 (25.9)  56/120 (46.7)  34/69 (49.3)  45/72 (62.5) | 44/111 (39.6)  60/111 (54.1)  39/69 (56.5)  47/70 (67.1) | 48/104 (46.2)  52/101 (51.5)  39/67 (58.2)  51/68 (75.0) | 43/101 (42.6)  59/101 (58.4)  43/66 (65.2)  47/65 (72.3) | 48/97 (49.5)  61/97 (62.9)  39/65 (60.0)  45/65 (69.2) | 36/91 (39.6)  62/90 (68.9)  43/63 (68.3)  44/64 (48.8) | 48/89 (53.9)  57/90 (63.3)  37/61 (60.7)  49/62 (79.0) | 48/91 (52.7)  54/88 (61.4)  41/61 (67.2)  42/59 (71.2) | 45/90 (50.0)  58/85 (68.2)  39/60 (65.0)  42/56 (75.0) |  |
| **ACR70** |  |  |  |  |  |  |  |  |  |  |  |  |
| UPA to ADA – NR  UPA to ADA – IR  ADA to UPA – NR  ADA to UPA – IR | 1/125 (0.8)  9/123 (7.3)  0/77 (0.0)  2/80 (2.5) | 10/120 (8.3)  20/122 (16.4)  13/74 (17.6)  23/76 (30.3) | 14/114 (12.3)  22/119 (18.5)  17/72 (23.6)  29/74 (39.2) | 20/113 (17.7)  34/112 (30.4)  22/68 (32.4)  28/70 (40.0) | 29/107 (27.1)  31/101 (30.7)  22/69 (31.9)  35/68 (51.5) | 21/103 (20.4)  32/101 (31.7)  22/66 (33.3)  34/65 (52.3) | 29/97 (29.9)  40/97 (41.2)  23/63 (36.5)  28/65 (43.1) | 24/92 (26.1)  33/91 (36.3)  28/61 (45.9)  30/63 (47.6) | 27/90 (30.0)  37/92 (40.2)  21/61 (34.4)  35/62 (56.5) | 30/91 (33.0)  31/90 (34.4)  24/62 (38.7)  29/60 (48.3) | 26/90 (28.9)  33/86 (38.4)  28/59 (47.5)  30/54 (55.6) |  |
| **Weeks post-switch** | | | | | | | | | | | | |
| **n/N (%)** | **132** | **144** | **156** | **168** | **180** | **192** | **204** | **216** | **228** | **240** | **252** |  |
| **ACR20** |  |  |  |  |  |  |  |  |  |  |  |  |
| UPA to ADA – NR  UPA to ADA – IR  ADA to UPA – NR  ADA to UPA – IR | 68/84 (81.0)  74/85 (87.1)  50/57 (87.7)  52/57 (91.2) | 64/76 (84.2)  68/83 (81.9)  50/57 (87.7)  52/57 (91.2) | 60/77 (77.9)  71/78 (91.0)  47/52 (90.4)  48/50 (96.0) | 65/76 (85.5)  69/77 (89.6)  46/55 (83.6)  53/58 (91.4) | 61/75 (81.3)  66/72 (91.7)  46/52 (88.5)  49/55 (89.1) | 57/73 (78.1)  66/72 (91.7)  49/54 (90.7)  48/52 (92.3) | 59/72 (81.9)  59/74 (79.7)  52/55 (94.5)  47/53 (88.7) | 58/74 (78.4)  63/75 (84.0)  48/53 (90.6)  48/50 (96.0) | 58/72 (80.6)  61/66 (92.4)  47/52 (90.4)  43/48 (89.6) | 43/56 (76.8)  63/70 (90.0)  40/44 (90.9)  43/48 (89.6) | 45/55 (81.8)  62/71 (87.3)  40/44 (90.9)  45/49 (91.8) |  |
| **ACR50** |  |  |  |  |  |  |  |  |  |  |  |  |
| UPA to ADA – NR  UPA to ADA – IR  ADA to UPA – NR  ADA to UPA – IR | 44/83 (53.0)  51/83 (61.4)  37/56 (66.1)  44/56 (78.6) | 42/75 (56.0)  53/82 (64.6)  41/57 (71.9)  43/54 (79.6) | 42/78 (53.8)  56/79 (70.9)  38/52 (73.1)  40/52 (76.9) | 43/76 (56.6)  47/75 (62.7)  37/54 (68.5)  43/56 (76.8) | 43/76 (56.6)  54/73 (74.0)  30/52 (57.7)  38/54 (70.4) | 36/72 (50.0)  46/71 (64.8)  40/54 (74.1)  38/51 (74.5) | 39/70 (55.7)  44/71 (62.0)  42/54 (77.8)  41/52 (78.8) | 39/73 (53.4)  45/73 (61.6)  39/52 (75.0)  40/49 (81.6) | 42/71 (59.2)  48/65 (73.8)  33/51 (64.7)  34/48 (70.8) | 33/56 (58.9)  48/71 (67.6)  33/44 (75.0)  35/47 (74.5) | 30/54 (55.6)  47/69 (68.1)  31/44 (70.5)  35/47 (74.5) |  |
| **ACR70** |  |  |  |  |  |  |  |  |  |  |  |  |
| UPA to ADA – NR  UPA to ADA – IR  ADA to UPA – NR  ADA to UPA – IR | 26/84 (31.0)  31/85 (36.5)  24/57 (42.1)  36/55 (65.5) | 24/76 (31.6)  31/80 (38.8)  25/57 (43.9)  31/56 (55.4) | 26/77 (33.8)  38/76 (50.0)  21/53 (39.6)  31/52 (59.6) | 22/76 (28.9)  32/77 (41.6)  26/54 (48.1)  35/56 (62.5) | 28/76 (36.8)  35/72 (48.6)  19/53 (35.8)  29/54 (53.7) | 25/73 (34.2)  31/70 (44.3)  25/53 (47.2)  29/50 (58.0) | 25/71 (35.2)  28/72 (38.9)  26/54 (48.1)  26/50 (52.0) | 26/73 (35.6)  31/72 (43.1)  25/49 (51.0)  26/47 (55.3) | 30/71 (42.3)  25/62 (40.3)  23/52 (44.2)  24/48 (50.0) | 18/55 (32.7)  30/69 (43.5)  20/44 (45.5)  28/47 (59.6) | 20/54 (37.0)  35/71 (49.3)  22/44 (50.0)  30/47 (63.8) |  |
| **Weeks post-switch** | | | | | | | | | | | | |
| **n/N (%)** | **264** | **276** | **288** | **300** | **312** | **324** | **336** |  |  |  |  |  |
| **ACR20** |  |  |  |  |  |  |  |  |  |  |  |  |
| UPA to ADA – NR  UPA to ADA – IR  ADA to UPA – NR  ADA to UPA – IR | 44/54 (81.5)  59/67 (88.1)  40/43 (93.0)  45/49 (91.8) | 45/54 (83.3)  60/65 (92.3)  37/42 (88.1)  41/49 (83.7) | 43/51 (84.3)  60/67 (89.6)  34/41 (82.9)  45/50 (90.0) | 41/50 (82.0)  53/64 (82.8)  34/39 (87.2)  44/50 (88.0) | 38/46 (82.6)  54/62 (87.1)  32/39 (82.1)  42/49 (85.7) | 32/46 (69.6)  56/62 (90.3)  36/40 (90.0)  42/49 (85.7) | 33/43 (76.7)  53/60 (88.3)  36/40 (90.0)  45/50 (90.0) |  |  |  |  |  |
| **ACR50** |  |  |  |  |  |  |  |  |  |  |  |  |
| UPA to ADA – NR  UPA to ADA – IR  ADA to UPA – NR  ADA to UPA – IR | 29/53 (54.7)  48/68 (70.6)  27/42 (64.3)  37/48 (77.1) | 28/51 (54.9)  47/65 (72.3)  24/41 (58.5)  34/48 (70.8) | 28/51 (54.9)  43/65 (66.2)  27/42 (64.3)  34/48 (70.8) | 29/50 (58.0)  41/63 (65.1)  28/39 (71.8)  35/48 (72.9) | 23/46 (50.0)  40/63 (63.5)  26/39 (66.7)  34/47 (72.3) | 21/45 (46.7)  41/61 (67.2)  26/40 (65.0)  35/49 (71.4) | 22/42 (52.4)  40/60 (66.7)  25/39 (64.1)  32/49 (65.3) |  |  |  |  |  |
| **ACR70** |  |  |  |  |  |  |  |  |  |  |  |  |
| UPA to ADA – NR  UPA to ADA – IR  ADA to UPA – NR  ADA to UPA – IR | 17/53 (32.1)  31/67 (46.3)  16/44 (36.4)  26/46 (56.5) | 18/52 (34.6)  30/64 (46.9)  13/42 (31.0)  21/47 (44.7) | 15/50 (30.0)  33/66 (50.0)  17/42 (40.5)  25/48 (52.1) | 19/50 (38.0)  26/63 (41.3)  19/40 (47.5)  24/47 (51.1) | 13/45 (28.9)  29/63 (46.0)  17/39 (43.6)  25/47 (53.2) | 17/45 (37.8)  27/58 (46.6)  17/39 (43.6)  25/49 (51.0) | 14/43 (32.6)  31/59 (52.5)  15/40 (37.5)  25/48 (52.1) |  |  |  |  |  |

ACR20/50/70, ≥20/50/70% improvement in American College of Rheumatology response criteria; ADA, adalimumab; AO, as observed; CDAI, Clinical Disease Activity Index; DAS28(CRP), 28-joint Disease Activity Score based on C-reactive protein; IR, incomplete responders; LDA, low disease activity; NR, non-responders; UPA, upadacitinib.

**Table S11** Proportions of patients achieving CDAI LDA (≤10)/remission (≤2.8), DAS28(CRP) ≤3.2/<2.6 through 372 weeks (NRI)

| **Weeks** | | | | | | | | | | | | | | | | | | | | | |
| --- | --- | --- | --- | --- | --- | --- | --- | --- | --- | --- | --- | --- | --- | --- | --- | --- | --- | --- | --- | --- | --- |
| **n/N (%)** | **12** | | **26** | | **48** | | **60** | | **72** | | **84** | | **96** | | **108** | | **120** | **132** | | **144** |  |
| **CDAI ≤10** |  | |  | |  | |  | |  | |  | |  | |  | |  |  | |  |  |
| UPA  ADA | 263/651 (40.4)  98/327 (30.0) | | 343/651 (52.7)  125/327 (38.2) | | 299/651 (45.9)  107/327 (32.7) | | 299/651 (45.9)  107/327 (32.7) | | 294/651 (45.2)  104/327 (31.8) | | 290/651 (44.5)  99/327 (30.3) | | 285/651 (43.8)  98/327 (30.0) | | 276/651 (42.4)  100/327 (30.6) | | 277/651 (42.5)  99/327 (30.3) | 271/651 (41.6)  102/327 (31.2) | | 265/651 (40.7)  96/327 (29.4) |  |
| **CDAI ≤2.8** |  | |  | |  | |  | |  | |  | |  | |  | |  |  | |  |  |
| UPA  ADA | 87/651 (13.4)  25/327 (7.6) | | 150/651 (23.0)  45/327 (13.8) | | 166/651 (25.5)  55/327 (16.8) | | 160/651 (24.6)  49/327 (16.8) | | 183/651 (28.1)  54/327 (16.5) | | 176/651 (27.0)  56/327 (17.1) | | 177/651 (27.2)  53/327 (16.2) | | 163/651 (25.0)  53/327 (16.2) | | 169/651 (26.0)  51/327 (15.6) | 170/651 (26.1)  55/327 (16.8) | | 159/651 (24.4)  60/327 (18.3) |  |
| **DAS28(CRP)** **≤3.2** | |  | |  | |  | |  | |  | |  | |  | |  | |  | |  |  |
| UPA  ADA | 293/651 (45.0)  94/327 (28.7) | | 356/651 (54.7)  126/327 (38.5) | | 297/651 (45.6)  105/327 (32.1) | | 286/651 (43.9)  99/327 (30.3) | | 293/651 (45.0)  96/327 (29.4) | | 283/651 (43.5)  93/327 (28.4) | | 282/651 (43.3)  93/327 (28.4) | | 284/651 (43.6)  104/327 (31.8) | | 275/651 (42.2)  95/327 (29.1) | 269/651 (41.3)  95/327 (29.1) | | 253/651 (38.9)  90/327 (27.5) |  |
| **DAS28(CRP)** **<2.6** | |  | |  | |  | |  | |  | |  | |  | |  | |  | |  |  |
| UPA  ADA | 187/651 (28.7)  59/327 (18.0) | | 266/651 (40.9)  88/327 (26.9) | | 243/651 (37.3)  87/327 (26.6) | | 247/651 (37.9)  79/327 (24.2) | | 259/651 (39.8)  83/327 (25.4) | | 256/651 (39.3)  83/327 (25.4) | | 242/651 (37.2)  78/327 (23.9) | | 238/651 (36.6)  84/327 (25.7) | | 242/651 (37.2)  79/327 (24.2) | 236/651 (36.3)  79/327 (24.2) | | 215/651 (33.0)  76/327 (23.2) |  |
| **Weeks** | | | | | | | | | | | | | | | | | | | | | |
| **n/N (%)** | **156** | **168** | | | **180** | | **192** | | **204** | | **216** | | **228** | | **240** | | **252** | **264** | | **276** |  |
| **CDAI ≤10** |  | |  | |  | |  | |  | |  | |  | |  | |  |  | |  |  |
| UPA  ADA | 257/651 (39.5)  96/327 (29.4) | | 262/651 (40.2)  94/327 (28.7) | | 260/651 (39.9)  94/327 (28.7) | | 252/651 (38.7)  93/327 (28.4) | | 241/651 (37.0)  89/327 (27.2) | | 252/651 (38.7)  89/327 (27.2) | | 243/651 (37.3)  91/327 (27.8) | | 245/651 (37.6)  92/327 (28.1) | | 238/651 (36.6)  87/327 (26.6) | 237/651 (36.4)  88/327 (26.9) | | 237/651 (36.4)  88/327 (26.9) |  |
| **CDAI ≤2.8** |  | |  | |  | |  | |  | |  | |  | |  | |  |  | |  |  |
| UPA  ADA | 159/651 (24.4)  54/327 (16.5) | | 164/651 (25.2)  59/327 (18.0) | | 165/651 (25.3)  57/327 (17.4) | | 171/651 (26.3)  56/327 (17.1) | | 166/651 (25.5)  55/327 (16.8) | | 165/651 (25.3)  58/327 (17.7) | | 168/651 (25.8)  61/327 (18.7) | | 157/651 (24.1)  65/327 (19.9) | | 160/651 (24.6)  59/327 (18.0) | 160/651 (24.6)  61/327 (18.7) | | 167/651 (25.7)  55/327 (16.8) |  |
| **DAS28(CRP) ≤3.2** | |  | |  | |  | |  | |  | |  | |  | | |  |  | |  |  |
| UPA  ADA | 245/651 (37.6)  85/327 (26.0) | | 252/651 (38.7)  78/327 (23.9) | | 238/651 (36.6)  78/327 (23.9) | | 240/651 (36.9)  83/327 (25.4) | | 227/651 (34.9)  78/327 (23.9) | | 231/651 (35.5)  75/327 (22.9) | | 236/651 (36.3)  79/327 (24.2) | | 229/651 (35.2)  83/327 (25.4) | | 225/651 (34.6)  76/327 (23.2) | 229/651 (35.2)  82/327 (25.1) | | 224/651 (34.4)  82/327 (25.1) |  |
| **DAS28(CRP) <2.6** | |  | | |  | |  | |  | |  | |  | |  | |  | |  | |  |
| UPA  ADA | 215/651 (33.0)  71/327 (21.7) | | 223/651 (34.3)  73/327 (22.3) | | 215/651 (33.0)  63/327 (19.3) | | 210/651 (32.3)  69/327 (21.1) | | 208/651 (32.0)  64/327 (19.6) | | 212/651 (32.6)  64/327 (19.6) | | 210/651 (32.3)  67/327 (20.5) | | 198/651 (30.4)  75/327 (22.9) | | 199/651 (30.6)  67/327 (20.5) | 210/651 (32.3)  77/327 (23.5) | | 204/651 (31.3)  76/327 (23.2) |  |
| **Weeks** | | | | | | | | | | | | | | | | | | | | | |
| **n/N (%)** | **288** | **300** | | | **312** | | **324** | | **336** | | **348** | | **360** | | **372** | |  |  | |  |  |
| **CDAI ≤10** |  |  | | |  | |  | |  | |  | |  | |  | |  |  | |  |  |
| UPA  ADA | 229/651 (35.2)  81/327 (24.8) | | 224/651 (34.4)  86/327 (26.3) | | 221/651 (33.9)  84/327 (25.7) | | 222/651 (34.1)  81/327 (24.8) | | 214/651 (32.9)  83/327 (25.4) | | 211/651 (32.4)  80/327 (24.5) | | 205/651 (31.5)  76/327 (23.2) | | 203/651 (31.2)  77/327 (23.5) | |  |  | |  |  |
| **CDAI ≤2.8** |  |  | | |  | |  | |  | |  | |  | |  | |  |  | |  |  |
| UPA  ADA | 144/651 (22.1)  57/327 (17.4) | | 152/651 (23.3)  59/327 (18.0) | | 138/651 (21.2)  56/327 (17.1) | | 154/651 (23.7)  52/327 (15.9) | | 137/651 (21.0)  58/327 (17.7) | | 149/651 (22.9)  53/327 (16.2) | | 137/651 (21.0)  45/327 (13.8) | | 142/651 (21.8)  43/327 (13.1) | |  |  | |  |  |
| **DAS28(CRP) ≤3.2** | |  | | |  | |  | |  | |  | |  | |  | |  | |  | |  |
| UPA  ADA | 221/651 (33.9)  78/327 (23.9) | | 215/651 (33.0)  79/327 (24.2) | | 210/651 (32.3)  82/327 (25.1) | | 210/651 (32.3)  77/327 (23.5) | | 205/651 (31.5)  79/327 (24.2) | | 205/651 (31.5)  77/327 (23.5) | | 199/651 (30.6)  69/327 (21.1) | | 187/651 (28.7)  68/327 (20.8) | |  |  | |  |  |
| **DAS28(CRP) <2.6** | |  | | |  | |  | |  | |  | |  | |  | |  | |  | |  |
| UPA  ADA | 189/651 (29.0)  71/327 (21.7) | | 192/651 (29.5)  71/327 (21.7) | | 187/651 (28.7)  72/327 (22.0) | | 180/651 (27.6)  70/327 (21.4) | | 187/651 (28.7)  72/327 (22.0) | | 186/651 (28.6)  67/327 (20.5) | | 178/651 (27.3)  59/327 (18.0) | | 172/651 (26.4)  55/327 (16.8) | |  |  | |  |  |

ADA, adalimumab; CDAI, Clinical Disease Activity Index; DAS28(CRP), 28-joint Disease Activity Score based on C-reactive protein; LDA, low disease activity; NRI, non-responder imputation; UPA, upadacitinib.

**Table S12**  Proportions of patients achieving ACR20, ACR50 and ACR70 through 372 weeks (NRI)

| **Weeks** | | | | | | | | | | | |
| --- | --- | --- | --- | --- | --- | --- | --- | --- | --- | --- | --- |
| **n/N (%)** | **12** | **26** | **48** | **60** | **72** | **84** | **96** | **108** | **120** | **132** | **144** |
| **ACR20** |  |  |  |  |  |  |  |  |  |  |  |
| UPA  ADA | 450/651 (69.1)  206/327 (63.0) | 436/651 (67.0)  187/327 (57.2) | 328/651 (50.4)  116/327 (35.5) | 316/651 (48.5)  117/327 (35.8) | 305/651 (46.9)  110/327 (33.6) | 308/651 (47.3)  107/327 (32.7) | 306/651 (47.0)  109/327 (33.3) | 302/651 (46.4)  108/327 (33.0) | 298/651 (45.8)  106/327 (32.4) | 288/651 (44.2)  107/327 (32.7) | 271/651 (41.6)  103/327 (31.5) |
| **ACR50** |  |  |  |  |  |  |  |  |  |  |  |
| UPA  ADA | 288/651 (44.2)  93/327 (28.4) | 349/651 (53.6)  137/327 (41.9) | 284/651 (43.6)  101/327 (30.9) | 279/651 (42.9)  101/327 (30.9) | 279/651 (42.9)  93/327 (28.4) | 282/651 (43.3)  96/327 (29.4) | 277/651 (42.5)  95/327 (29.1) | 262/651 (40.2)  94/327 (28.7) | 270/651 (41.5)  88/327 (26.9) | 253/651 (38.9)  94/327 (28.7) | 240/651 (36.9)  91/327 (27.8) |
| **ACR70** |  |  |  |  |  |  |  |  |  |  |  |
| UPA  ADA | 162/651 (24.9)  43/327 (13.1) | 224/651 (34.4)  75/327 (22.9) | 225/651 (34.6)  74/327 (22.6) | 226/651 (34.7)  69/327 (21.1) | 227/651 (34.9)  75/327 (22.9) | 223/651 (34.3)  75/327 (22.9) | 223/651 (34.3)  68/327 (20.8) | 219/651 (33.6)  74/327 (22.6) | 217/651 (33.3)  68/327 (20.8) | 209/651 (32.1)  73/327 (22.3) | 208/651 (32.0)  71/327 (21.7) |
| **Weeks** | | | | | | | | | | | |
| **n/N (%)** | **156** | **168** | **180** | **192** | **204** | **216** | **228** | **240** | **252** | **264** | **276** |
| **ACR20** |  |  |  |  |  |  |  |  |  |  |  |
| UPA  ADA | 281/651 (41.6)  98/327 (30.0) | 274/651 (42.1)  100/327 (30.6) | 273/651 (41.9)  99/327 (30.3) | 269/651 (41.3)  99/327 (30.3) | 264/651 (40.6)  94/327 (28.7) | 260/651 (39.9)  95/327 (29.1) | 261/651 (40.1)  96/327 (29.4) | 260/651 (39.9)  94/327 (28.7) | 252/651 (38.7)  92/327 (28.1) | 251/651 (38.6)  93/327 (28.4) | 246/651 (37.8)  91/327 (27.8) |
| **ACR50** |  |  |  |  |  |  |  |  |  |  |  |
| UPA  ADA | 249/651 (38.2)  87/327 (26.6) | 253/651 (38.9)  84/327 (25.7) | 253/651 (38.9)  90/327 (27.5) | 252/651 (38.7)  88/327 (26.9) | 241/651 (37.0)  84/327 (25.7) | 241/651 (37.0)  84/327 (25.7) | 235/651 (26.1)  89/327 (27.2) | 233/651 (35.8)  87/327 (26.6) | 234/651 (35.9)  85/327 (26.0) | 231/651 (35.5)  84/327 (25.7) | 224/651 (34.4)  85/327 (26.0) |
| **ACR70** |  |  |  |  |  |  |  |  |  |  |  |
| UPA  ADA | 211/651 (32.4)  67/327 (20.5) | 205/651 (31.5)  70/327 (21.4) | 212/651 (32.6)  71/327 (21.7) | 210/651 (32.3)  72/327 (22.0) | 199/651 (30.6)  69/327 (21.1) | 201/651 (30.9)  65/327 (19.9) | 197/651 (30.3)  75/327 (22.9) | 200/651 (30.7)  73/327 (22.3) | 193/651 (29.6)  68/327 (20.8) | 187/651 (28.7)  73/327 (22.3) | 186/651 (28.6)  66/327 (20.2) |
| **Weeks** | | | | | | | | | | | |
| **n/N (%)** | **288** | **300** | **312** | **324** | **336** | **348** | **360** | **372** |  |  |  |
| **ACR20** |  |  |  |  |  |  |  |  |  |  |  |
| UPA  ADA | 240/651 (36.9)  88/327 (26.9) | 239/651 (36.7)  88/327 (26.9) | 232/651 (35.6)  89/327 (27.2) | 231/651 (35.5)  86/327 (26.3) | 225/651 (34.6)  89/327 (27.2) | 219/651 (33.6)  84/327 (25.7) | 216/651 (33.2)  83/327 (25.4) | 209/651 (32.1)  82/327 (25.1) |  |  |  |
| **ACR50** |  |  |  |  |  |  |  |  |  |  |  |
| UPA  ADA | 218/651 (33.5)  81/327 (24.8) | 219/651 (33.6)  83/327 (25.4) | 214/651 (32.9)  79/327 (24.2) | 210/651 (32.3)  74/327 (22.6) | 203/651 (31.2)  85/327 (26.0) | 199/651 (30.6)  76/327 (23.2) | 192/651 (29.5)  73/327 (22.3) | 195/651 (30.0)  75/327 (22.9) |  |  |  |
| **ACR70** |  |  |  |  |  |  |  |  |  |  |  |
| UPA  ADA | 178/651 (27.3)  60/327 (18.3) | 182/651 (28.0)  62/327 (19.0) | 180/651 (27.6)  59/327 (18.0) | 174/651 (26.7)  57/327 (17.4) | 169/651 (26.0)  62/327 (19.0) | 167/651 (25.7)  63/327 (19.3) | 162/651 (24.9)  59/327 (18.0) | 167/651 (25.7)  56/327 (17.1) |  |  |  |

ACR20/50/70, ≥20/50/70% improvement in American College of Rheumatology response criteria; ADA, adalimumab; LDA, low disease activity; NRI, non-responder imputation; UPA, upadacitinib.

**Table S13**  Mean change from BL in core components of ACR criteria through 372 weeks (AO)

| **Weeks** | | | | | | | | | | | |
| --- | --- | --- | --- | --- | --- | --- | --- | --- | --- | --- | --- |
| **n/N (mean change from BL)** | **12** | **26** | **48** | **60** | **72** | **84** | **96** | **108** | **120** | **132** | **144** |
| **TJC68**  PBO to UPA  ADA continued  ADA to UPA  UPA continued  UPA to ADA | 625/650 (-9.7)  155/168 (-17.6)  159/159 (-11.6)  374/399 (-18.6)  250/252 (-13.8) | 607/650 (-17.5)  137/168 (-20.9)  157/159 (-16.6)  360/399 (-21.6)  248/252 (-17.9) | 582/650 (-20.9)  134/168 (-20.6)  146/159 (-21.0)  347/399 (-21.5)  237/252 (-21.9) | 570/650 (-21.7)  132/168 (-21.2)  142/159 (-22.2)  342/399 (-21.7)  223/252 (-23.0) | 553/650 (-22.3)  130/168 (-21.1)  138/159 (-23.5)  334/399 (-21.9)  207/252 (-23.8) | 540/650 (-22.5)  128/168 (-20.7)  134/159 (-23.1)  329/399 (-21.7)  203/252 (-24.2) | 527/650 (-22.7)  127/168 (-21.1)  131/159 (-23.0)  327/399 (-21.8)  194/252 (-24.4) | 516/650 (-22.8)  124/168 (-20.9)  128/159 (-24.0)  321/399 (-21.8)  186/252 (-24.8) | 505/650 (-23.2)  122/168 (-21.4)  126/159 (-23.6)  319/399 (-21.9)  185/252 (-25.0) | 491/650 (-23.4)  118/168 (-21.1)  125/159 (-24.2)  314/399 (-21.7)  183/252 (-24.5) | 469/650 (-23.6)  117/168 (-21.1)  117/159 (-24.8)  297/399 (-21.9)  178/252 (-24.9) |
| **SJC66**  PBO to UPA  ADA continued  ADA to UPA  UPA continued  UPA to ADA | 625/650 (-7.0)  155/168 (-11.8)  159/159 (-9.0)  374/399 (-13.2)  250/252 (-9.0) | 607/650 (-11.7)  137/168 (-13.8)  157/159 (-12.3)  360/399 (-14.8)  248/252 (-11.4) | 582/650 (-13.6)  134/168 (-13.6)  146/159 (-14.4)  347/399 (-14.9)  237/252 (-13.8) | 570/650 (-14.0)  132/168 (-13.9)  142/159 (-14.6)  342/399 (-14.9)  223/252 (-13.7) | 553/650 (-14.5)  130/168 (-13.7)  138/159 (-15.4)  334/399 (-15.0)  207/252 (-14.1) | 540/650 (-14.5)  128/168 (-13.5)  134/159 (-14.8)  329/399 (-14.8)  203/252 (-14.3) | 527/650 (-14.7)  127/168 (-13.6)  131/159 (-15.2)  327/399 (-15.0)  194/252 (-14.4) | 516/650 (-14.5)  124/168 (-13.7)  128/159 (-15.4)  321/399 (-14.9)  186/252 (-14.7) | 505/650 (-14.9)  122/168 (-13.4)  126/159 (-15.8)  319/399 (-14.8)  185/252 (-14.6) | 491/650 (-15.0)  118/168 (-13.4)  125/159 (-16.0)  314/399 (-14.8)  183/252 (-14.7) | 469/650 (-15.1)  117/168 (-13.3)  117/159 (-16.2)  297/399 (-15.0)  178/252 (-14.9) |
| **PtGA**  PBO to UPA  ADA continued  ADA to UPA  UPA continued  UPA to ADA | 622/648 (-15.5)  155/168 (-29.1)  157/157 (-20.7)  373/397 (-36.7)  247/250 (-21.9) | 605/648 (-30.2)  137/168 (-41.9)  156/157 (-28.9)  358/397 (-46.1)  246/250 (-23.8) | 579/648 (-37.3)  134/168 (-42.7)  145/157 (-38.6)  345/397 (-46.4)  233/250 (-30.2) | 566/648 (-37.8)  132/168 (-41.5)  140/157 (-38.5)  339/397 (-45.9)  220/250 (-32.0) | 551/648 (-40.2)  130/168 (-42.1)  137/157 (-40.1)  331/397 (-46.7)  205/250 (-31.9) | 539/648 (-40.8)  128/168 (-41.1)  132/157 (-40.1)  327/397 (-47.2)  201/250 (-35.5) | 524/648 (-41.3)  125/168 (-41.0)  129/157 (-44.0)  325/397 (-46.7)  193/250 (-35.7) | 512/648 (-41.5)  124/168 (-44.4)  127/157 (-40.6)  318/397 (-46.3)  185/250 (-35.3) | 501/648 (-43.1)  123/168 (-41.4)  125/157(-43.4)  317/397 (-47.8)  184/250 (-35.6) | 489/648 (-42.8)  118/168 (-45.3)  124/157 (-42.0)  310/397 (-47.5)  182/250 (-36.0) | 463/648 (-42.7)  117/168 (-45.2)  116/157 (-45.4)  293/397 (-47.6)  176/250 (-36.7) |
| **PhGA**  PBO to UPA  ADA continued  ADA to UPA  UPA continued  UPA to ADA | 588/619 (-25.3)  141/160 (-39.5)  144/145 (-32.5)  347/375 (-45.5)  235/241 (-29.4) | 573/619 (-41.8)  132/160 (-48.9)  142/145 (-40.7)  339/375 (-54.7)  237/241 (-35.6) | 544/619 (-51.0)  126/160 (-50.9)  131/145 (-48.7)  324/375 (-55.2)  219/241 (-42.0) | 528/619 (-52.3)  125/160 (-51.1)  127/145 (-51.2)  320/375 (-55.7)  207/241 (-45.3) | 513/619 (-53.7)  120/160 (-52.6)  122/145 (-53.7)  311/375 (-56.1)  195/241 (-46.0) | 508/619 (-54.3)  122/160 (-52.2)  118/145 (-53.2)  309/375 (-56.5)  193/241 (-49.1) | 489/619 (-55.1)  120/160 (-51.5)  117/145 (-54.8)  307/375 (-57.0)  184/241 (-47.8) | 480/619 (-54.8)  116/160 (-53.3)  112/145 (-54.0)  295/375 (-56.1)  177/241 (-48.2) | 470/619 (-55.8)  114/160 (-51.7)  112/145 (-53.9)  294/375 (-57.0)  170/241 (-51.2) | 461/619 (-56.6)  110/160 (-52.3)  112/145 (-54.3)  293/375 (-56.5)  173/241 (-49.5) | 430/619 (-56.4)  110/160 (-53.7)  103/145 (-55.2)  277/375 (-56.7)  170/241 (-48.2) |
| **PtPain**  PBO to UPA  ADA continued  ADA to UPA  UPA continued  UPA to ADA | 622/648 (-15.6)  155/168 (-31.5)  157/157 (-21.2)  373/397 (-37.8)  247/250 (-24.1) | 605/648 (-31.4)  137/168 (-44.1)  156/157 (-28.9)  358/397 (-46.1)  246/250 (-26.2) | 579/648 (-39.3)  134/168 (-43.8)  145/157 (-39.3)  345/397 (-46.6)  233/250 (-32.5) | 566/648 (-39.4)  132/168 (-42.5)  140/157 (-38.1)  339/397 (-46.5)  220/250 (-34.3) | 551/648 (-41.5)  130/168 (-42.3)  137/157 (-41.4)  331/397 (-47.1)  205/250 (-33.3) | 539/648 (-41.9)  128/168 (-42.1)  132/157 (-40.8)  327/397 (-48.1)  201/250 (-36.4) | 524/648 (-41.9)  125/167 (-42.6)  130/157 (-44.3)  325/397 (-47.4)  193/250 (-37.9) | 512/648 (-42.5)  124/168 (-44.1)  127/157 (-41.2)  318/397 (-47.6)  185/250 (-38.2) | 501/648 (-43.2)  123/167 (-42.9)  125/157 (-41.3)  318/397 (-47.9)  184/250 (-38.9) | 489/648 (-43.0)  118/168 (-44.8)  124/157 (-41.7)  310/397 (-47.3)  182/250 (-37.7) | 463/648 (-43.1)  117/168 (-44.7)  116/157 (-43.4)  293/397 (-47.7)  176/250 (-39.2) |
| **HAQ-DI**  PBO to UPA  ADA continued  ADA to UPA  UPA continued  UPA to ADA | 622/648 (-0.32)  155/168 (-0.63)  157/157 (-0.46)  373/397 (-0.80)  246/249 (-0.43) | 604/648 (-0.59)  137/168 (-0.79)  156/157 (-0.60)  358/397 (-0.93)  245/249 (-0.51) | 579/648 (-0.76)  134/168 (-0.79)  145/157 (-0.75)  345/397 (-0.99)  232/249 (-0.59) | 566/648 (-0.77)  132/168 (-0.84)  140/157 (-0.81)  339/397 (-0.98)  219/249 (-0.67) | 551/648 (-0.81)  130/168 (-0.83)  137/157 (-0.84)  331/397 (-1.00)  204/249 (-0.67) | 539/648 (-0.82)  128/168 (-0.78)  132/157 (-0.79)  327/397 (-1.02)  200/249 (-0.70) | 524/648 (-0.84)  125/168 (-0.82)  130/157 (-0.84)  325/397 (-1.01)  192/249 (-0.73) | 512/648 (-0.82)  124/168 (-0.84)  127/157 (-0.84)  318/397 (-1.01)  184/249 (-0.73) | 501/648 (-0.86)  123/168 (-0.79)  125/157 (-0.84)  316/397 (-1.02)  182/249 (-0.75) | 488/648 (-0.84)  118/168 (-0.81)  124/157 (-0.84)  310/397 (-1.00)  181/249 (-0.70) | 463/648 (-0.86)  117/168 (-0.86)  116/157 (-0.91)  297/397 (-1.01)  175/249 (-0.71) |
| **hsCRP**  PBO to UPA  ADA continued  ADA to UPA  UPA continued  UPA to ADA | 595/650 (-1.8)  146/167 (-8.4)  153/159 (-12.5)  357/399 (-12.9)  233/252 (-12.4) | 605/650 (-8.1)  137/167 (-10.4)  157/159 (-15.1)  361/399 (-13.8)  248/252 (-12.1) | 578/650 (-11.9)  134/167 (-9.3)  146/159 (-17.1)  346/399 (-12.2)  237/252 (-10.4) | 563/650 (-13.1)  130/167 (-8.0)  140/159 (-17.6)  335/399 (-12.3)  219/252 (-9.9) | 550/650 (-12.4)  128/167 (-9.5)  134/159 (-17.5)  330/399 (-13.2)  206/252 (-10.7) | 527/650 (-12.2)  127/167 (-8.2)  132/159 (-16.8)  324/399 (-13.8)  201/252 (-10.6) | 510/650 (-12.6)  124/167 (-9.1)  129/159 (-15.8)  320/399 (-12.3)  188/252 (-10.2) | 502/650 (-13.9)  123/167 (-9.2)  120/159 (-17.1)  315/399 (-13.6)  182/252 (-9.7) | 499/650 (-12.9)  121/167 (-8.8)  124/159 (-17.8)  312/399 (-13.1)  181/252 (-10.5) | 474/650 (-13.4)  115/167 (-7.0)  116/159 (-17.5)  310/399 (-12.9)  177/252 (-12.5) | 435/650 (-13.5)  112/167 (-7.9)  108/159 (-18.7)  283/399 (-13.5)  164/252 (-12.6) |
| **Weeks** | | | | | | | | | | | |
| **n/N (mean change from BL)** | **156** | **168** | **180** | **192** | **204** | **216** | **228** | **240** | **252** | **264** | **276** |
| **TJC68**  PBO to UPA  ADA continued  ADA to UPA  UPA continued  UPA to ADA | 448/650 (-23.5)  113/168 (-21.5)  117/159 (-24.8)  291/399 (-21.8)  164/252 (-24.7) | 456/650 (-23.4)  112/168 (-21.7)  114/159 (-25.8)  293/399 (-22.2)  162/252 (-24.9) | 450/650 (-23.6)  112/168 (-21.1)  110/159 (-25.6)  290/399 (-21.7)  157/252 (-25.5) | 453/650 (-23.5)  113/168 (-21.2)  113/159 (-25.0)  289/399 (-21.6)  155/252 (-25.8) | 445/650 (-23.7)  109/168 (-21.5)  111/159 (-25.6)  284/399 (-21.6)  149/252 (-26.5) | 439/650 (-23.7)  106/168 (-21.4)  109/159 (-25.8)  281/399 (-21.8)  145/252 (-26.7) | 441/650 (-23.7)  105/168 (-21.9)  108/159 (-26.0)  282/399 (-21.6)  147/252 (-26.5) | 431/650 (-23.4)  106/168 (-21.9)  103/159 (-26.1)  277/399 (-21.8)  149/252 (-26.4) | 426/650 (-23.2)  101/168 (-22.0)  103/159 (-26.2)  275/399 (-21.4)  143/252 (-27.3) | 422/650 (-23.5)  101/168 (-22.0)  100/159 (-25.9)  269/399 (-22.1)  141/252 (-27.6) | 417/650 (-23.6)  100/168 (-22.0)  101/159 (-26.0)  263/399 (-21.9)  141/252 (-27.1) |
| **SJC66**  PBO to UPA  ADA continued  ADA to UPA  UPA continued  UPA to ADA | 448/650 (-14.9)  113/168 (-13.6)  117/159 (-16.3)  291/399 (-15.0)  164/252 (-14.4) | 456/650 (-14.9)  112/168 (-13.8)  114/159 (-16.6)  293/399 (-15.1)  162/252 (-14.8) | 450/650 (-15.0)  112/168 (-13.7)  110/159 (-16.4)  290/399 (-14.7)  157/252 (-15.4) | 453/650 (-15.0)  113/168 (-13.1)  113/159 (-16.0)  289/399 (-14.9)  155/252 (-15.2) | 455/650 (-15.3)  109/168 (-13.9)  111/159 (-16.5)  284/399 (-14.6)  149/252 (-15.4) | 439/650 (-15.2)  106/168 (-13.4)  109/159 (-16.9)  281/399 (-14.9)  145/252 (-15.4) | 441/650 (-15.2)  105/168 (-13.6)  108/159 (-16.3)  282/399 (-14.8)  147/252 (-15.6) | 431/650 (-15.2)  106/168 (-13.7)  103/159 (-16.8)  277/399 (-14.8)  149/252 (-15.5) | 426/650 (-15.2)  101/168 (-13.6)  103/159 (-16.9)  275/399 (-14.5)  143/252 (-16.0) | 422/650 (-15.1)  101/168 (-13.5)  100/159 (-16.9)  269/399 (-15.0)  141/252 (-16.2) | 417/650 (-14.9)  100/168 (-13.5)  101/159 (-16.9)  263/399 (-14.8)  141/252 (-15.6) |
| **PtGA**  PBO to UPA  ADA continued  ADA to UPA  UPA continued  UPA to ADA | 445/648 (-43.6)  112/168 (-44.4)  115/157 (-45.6)  288/397 (-48.4)  164/250 (-35.7) | 450/648 (-42.8)  112/168 (-45.7)  111/157 (-45.0)  289/397 (-48.9)  161/250 (-36.0) | 441/648 (-44.2)  111/168 (-47.6)  109/157 (-47.3)  289/397 (-48.4)  156/250 (-37.5) | 448/648 (-43.6)  113/168 (-47.8)  112/157 (-43.5)  286/397 (-48.6)  154/250 (-36.9) | 441/648 (-43.0)  109/168 (-47.0)  109/157 (-44.7)  281/397 (-48.2)  147/250 (-37.8) | 434/648 (-44.1)  106/168 (-45.7)  107/157 (-45.9)  279/397 (-48.2)  145/250 (-37.2) | 438/648 (-43.2)  105/168 (-49.4)  107/157 (-45.0)  280/397 (-47.5)  148/250 (-34.8) | 428/648 (-44.0)  105/168 (-49.5)  102/157 (-45.6)  273/397 (-47.8)  148/250 (-38.3) | 419/648 (-42.8)  99/168 (-48.1)  101/157 (-42.8)  271/397 (-47.4)  139/250 (-38.9) | 419/648 (-42.6)  101/168 (-47.9)  99/157 (-45.5)  266/397 (-48.8)  140/250 (-38.5) | 414/648 (-44.6)  100/168 (-48.0)  99/157 (-44.4)  260/397 (-47.7)  141/250 (-37.3) |
| **PhGA**  PBO to UPA  ADA continued  ADA to UPA  UPA continued  UPA to ADA | 418/619 (-56.4)  105/160 (-54.2)  102/145 (-55.9)  268/375 (-56.8)  152/241 (-51.0) | 420/619 (-57.1)  106/160 (-53.7)  102/145 (-56.2)  275/375 (-56.0)  153/241 (-50.3) | 411/619 (-56.6)  106/160 (-53.8)  99/145 (-56.6)  273/375 (-56.1)  145/241 (-51.8) | 410/619 (-56.1)  106/160 (-53.5)  99/145 (-55.2)  269/375 (-55.1)  147/241 (-50.7) | 413/619 (-56.6)  104/160 (-53.0)  99/145 (-55.9)  266/375 (-57.6)  142/241 (-51.7) | 409/619 (-56.1)  101/160 (-52.5)  98/145 (-54.6)  264/375 (-56.2)  137/241 (-51.1) | 410/619 (-56.1)  99/160 (-55.0)  96/145 (-54.9)  262/375 (-55.6)  140/241 (-51.6) | 399/619 (-56.3)  100/160 (-55.1)  92/145 (-57.5)  256/375 (-56.7)  137/241 (-52.5) | 392/619 (-55.1)  92/160 (-54.7)  92/145 (-56.8)  251/375 (-55.5)  131/241 (-54.7) | 394/619 (-54.4)  94/160 (-53.7)  90/145 (-56.4)  247/375 (-56.8)  132/241 (-55.2) | 387/619 (-56.1)  95/160 (-54.6)  89/145 (-57.9)  247/375 (-56.9)  136/241 (-51.7) |
| **PtPain**  PBO to UPA  ADA continued  ADA to UPA  UPA continued  UPA to ADA | 445/648 (-44.2)  112/167 (-42.6)  115/157 (-44.8)  288/397 (-49.0)  164/250 (-39.0) | 450/648 (-43.1)  112/168 (-44.4)  111/157 (-44.5)  289/397 (-49.2)  161/250 (-39.5) | 441/648 (-44.4)  111/168 (-46.8)  109/157 (-45.8)  289/397 (-48.0)  156/250 (-40.3) | 448/648 (-44.0)  113/168 (-47.3)  112/157 (-43.5)  286/397 (-48.6)  154/250 (-39.7) | 441/648 (-42.8)  109/168 (-46.7)  109/157 (-43.8)  281/397 (-48.7)  147/250 (-41.1) | 434/648 (-43.4)  106/168 (-44.6)  107/157 (-44.9)  279/397 (-48.9)  145/250 (-40.7) | 438/648 (-43.3)  105/168 (-47.8)  107/157 (-43.6)  280/397 (-47.6)  148/250 (-37.3) | 428/648 (-45.1)  105/168 (-49.0)  102/157 (-43.0)  273/397 (-48.5)  148/250 (-41.0) | 419/648 (-43.6)  99/168 (-46.8)  101/157 (-41.8)  271/397 (-48.8)  139/250 (-41.0) | 419/648 (-43.1)  101/168 (-47.6)  99/157 (-44.0)  266/397 (-48.4)  140/250 (-41.3) | 414/648 (-43.7)  100/168 (-47.9)  99/157 (-44.2)  260/397 (-48.9)  141/250 (-39.1) |
| **HAQ-DI**  PBO to UPA  ADA continued  ADA to UPA  UPA continued  UPA to ADA | 444/648 (-0.86)  112/168 (-0.84)  115/157 (-0.91)  288/397 (-1.00)  163/249 (-0.75) | 448/648 (-0.86)  112/168 (-0.83)  111/157 (-0.92)  289/397 (-1.00)  160/249 (-0.70) | 441/648 (-0.86)  110/168 (-0.90)  109/157 (-0.92)  289/397 (-0.99)  155/249 (-0.77) | 448/648 (-0.86)  113/168 (-0.85)  112/157 (-0.91)  286/397 (-1.01)  153/249 (-0.73) | 441/648 (-0.86)  109/168 (-0.89)  109/157 (-0.91)  281/397 (-1.01)  146/249 (-0.74) | 434/648 (-0.87)  106/168 (-0.80)  107/157 (-0.86)  279/397 (-0.98)  144/249 (-0.75) | 437/648 (-0.88)  105/168 (-0.88)  107/157 (-0.91)  280/397 (-0.99)  147/249 (-0.70) | 428/648 (-0.87)  105/168 (-0.91)  102/157 (-0.83)  273/397 (-1.00)  147/249 (-0.76) | 419/648 (-0.86)  99/168 (-0.85)  101/157 (-0.87)  271/397 (-0.98)  138/249 (-0.75) | 419/648 (-0.86)  101/168 (-0.88)  99/157 (-0.89)  266/397 (-0.98)  138/249 (-0.78) | 414/648 (-0.88)  100/168 (-0.84)  99/157 (-0.85)  260/397 (-0.98)  140/249 (-0.75) |
| **hsCRP**  PBO to UPA  ADA continued  ADA to UPA  UPA continued  UPA to ADA | 406/650 (-14.9)  101/167 (-9.5)  107/159 (-18.4)  268/399 (-14.1)  153/252 (-12.0) | 403/650 (-13.5)  96/167 (-10.2)  100/159 (-21.1)  271/399 (-13.4)  148/252 (-10.3) | 405/650 (-14.0)  96/167 (-9.8)  95/159 (-19.5)  262/399 (-13.8)  145/252 (-13.3) | 417/650 (-13.9)  103/167 (-9.3)  105/159 (-18.0)  264/399 (-13.5)  145/252 (-11.7) | 410/650 (-13.5)  98/167 (-9.7)  102/159 (-16.5)  262/399 (-12.7)  137/252 (-12.5) | 395/650 (-14.5)  94/167 (-7.6)  101/159 (-17.3)  259/399 (-12.6)  137/252 (-13.1) | 414/650 (-13.7)  92/167 (-7.3)  102/159 (-17.1)  269/399 (-11.9)  140/252 (-9.7) | 404/650 (-13.8)  97/167 (-10.3)  97/159 (-16.0)  256/399 (-12.5)  139/252 (-13.3) | 389/650 (-12.7)  90/167 (-8.2)  94/159 (-16.7)  257/399 (-13.1)  134/252 (-12.3) | 392/650 (-13.5)  96/167 (-9.9)  94/159 (-19.2)  254/399 (-12.3)  132/252 (-13.3) | 388/650 (-13.3)  96/167 (-9.1)  91/159 (-19.1)  249/399 (-13.4)  129/252 (-13.0) |
| **Weeks** | | | | | | | | | | | |
| **n/N (mean change from BL)** | **288** | **300** | **312** | **324** | **336** | **348** | **360** | **372** |  |  |  |
| **TJC68**  PBO to UPA  ADA continued  ADA to UPA  UPA continued  UPA to ADA | 411/650 (-23.5)  97/168 (-22.2)  100/159 (-26.1)  265/399 (-21.6)  138/252 (-26.9) | 401/650 (-24.0)  97/168 (-22.4)  100/159 (-25.4)  258/399 (-22.0)  132/252 (-28.0) | 384/650 (-23.7)  96/168 (-22.3)  99/159 (-25.7)  256/399 (-22.1)  133/252 (-27.7) | 384/650 (-23.7)  95/168 (-22.1)  98/159 (-26.1)  252/399 (-22.2)  129/252 (-27.3) | 372/650 (-24.0)  94/168 (-22.4)  98/159 (-26.0)  244/399 (-22.6)  126/252 (-27.0) | 365/650 (-24.0)  93/168 (-21.9)  98/159 (-26.6)  243/399 (-23.0)  120/252 (-27.7) | 364/650 (-23.9)  90/168 (-22.1)  97/159 (-26.6)  239/399 (-22.7)  121/252 (-27.8) | 357/650 (-24.2)  87/168 (-22.3)  93/159 (-27.5)  235/399 (-22.5)  116/252 (-27.6) |  |  |  |
| **SJC66**  PBO to UPA  ADA continued  ADA to UPA  UPA continued  UPA to ADA | 411/650 (-15.0)  97/168 (-13.7)  100/159 (-16.9)  265/399 (-14.8)  138/252 (-15.7) | 401/650 (-15.0)  97/168 (-13.6)  100/159 (-16.8)  258/399 (-15.0)  132/252 (-16.1) | 384/650 (-14.8)  96/168 (-13.7)  99/159 (-17.1)  256/399 (-15.0)  133/252 (-16.1) | 384/650 (-15.0)  95/168 (-13.5)  98/159 (-17.0)  252/399 (-15.0)  129/252 (-15.7) | 372/650 (-15.0)  94/168 (-13.7)  98/159 (-17.1)  244/399 (-15.1)  126/252 (-15.5) | 365/650 (-15.0)  93/168 (-13.5)  98/159 (-16.9)  243/399 (-15.3)  120/252 (-15.8) | 364/650 (-14.9)  90/168 (-13.6)  97/159 (-17.0)  239/399 (-15.3)  121/252 (-16.3) | 357/650 (-15.0)  87/168 (-13.8)  93/159 (-16.9)  235/399 (-15.3)  116/252 (-16.2) |  |  |  |
| **PtGA**  PBO to UPA  ADA continued  ADA to UPA  UPA continued  UPA to ADA | 405/648 (-42.4)  96/168 (-48.8)  99/157 (-41.7)  262/397 (-46.3)  139/250 (-37.0) | 398/648 (-43.7)  97/168 (-49.5)  99/157 (-43.9)  257/397 (-47.6)  132/250 (-38.3) | 381/648 (-43.7)  95/168 (-47.8)  97/157 (-43.7)  255/397 (-46.9)  132/250 (-37.7) | 379/648 (-44.4)  95/168 (-46.4)  96/157 (-41.7)  251/397 (-46.8)  126/250 (-35.6) | 368/648 (-44.2)  94/168 (-49.4)  97/157 (-42.8)  243/397 (-46.9)  124/250 (-35.2) | 355/648 (-44.9)  93/168 (-48.1)  97/157 (-43.0)  242/397 (-47.3)  120/250 (-35.8) | 355/648 (-44.0)  90/168 (-48.2)  96/157 (-41.9)  237/397 (-45.6)  117/250 (-36.6) | 351/648 (-44.8)  87/168 (-48.8)  90/157 (-44.1)  231/397 (-47.2)  113/250 (-38.2) |  |  |  |
| **PhGA**  PBO to UPA  ADA continued  ADA to UPA  UPA continued  UPA to ADA | 382/619 (-54.7)  90/160 (-53.3)  87/145 (-54.0)  246/375 (-55.2)  128/241 (-51.6) | 372/619 (-56.8)  92/160 (-55.7)  89/145 (-54.8)  242/375 (-55.7)  120/241 (-53.6) | 357/619 (-56.7)  90/160 (-54.6)  88/145 (-55.2)  234/375 (-55.8)  126/241 (-53.4) | 355/619 (-56.6)  88/160 (-54.6)  90/145 (-55.5)  238/375 (-55.6)  118/241 (-51.6) | 342/619 (-57.2)  87/160 (-53.8)  88/145 (-55.6)  230/375 (-55.9)  118/241 (-51.7) | 331/619 (-56.6)  87/160 (-52.4)  90/145 (-55.3)  227/375 (-55.8)  113/241 (-53.7) | 333/619 (-56.5)  84/160 (-55.7)  88/145 (-54.9)  219/375 (-55.6)  112/241 (-55.7) | 328/619 (-56.9)  82/160 (-55.2)  83/145 (-56.3)  218/375 (-56.0)  106/241 (-53.2) |  |  |  |
| **PtPain**  PBO to UPA  ADA continued  ADA to UPA  UPA continued  UPA to ADA | 405/648 (-42.4)  96/168 (-48.9)  99/157 (-41.3)  262/397 (-47.2)  138/250 (-39.8) | 398/648 (-43.8)  97/168 (-48.9)  99/157 (-42.6)  157/397 (-47.6)  132/250 (-42.1) | 381/648 (-44.5)  95/168 (-46.7)  97/157 (-43.0)  255/397 (-47.5)  132/250 (-40.4) | 378/648 (-44.4)  95/168 (-47.6)  96/157 (-41.9)  251/397 (-47.3)  126/250 (-39.7) | 368/648 (-44.2)  94/168 (-48.4)  97/157 (-44.5)  243/397 (-47.8)  124/250 (-38.9) | 355/648 (-44.6)  93/168 (-47.2)  97/157 (-41.5)  242/397 (-47.5)  120/250 (-39.7) | 355/648 (-45.0)  90/168 (-46.8)  96/157 (-41.1)  237/397 (-46.5)  117/250 (-40.1) | 351/648 (-44.6)  87/168 (-47.1)  90/157 (-42.3)  231/397 (-47.6)  113/250 (-41.3) |  |  |  |
| **HAQ-DI**  PBO to UPA  ADA continued  ADA to UPA  UPA continued  UPA to ADA | 405/648 (-0.86)  96/168 (-0.82)  99/157 (-0.80)  262/397 (-0.94)  137/249 (-0.76) | 397/648 (-0.88)  97/168 (-0.82)  99/157 (-0.82)  256/397 (-0.99)  131/249 (-0.78) | 380/648 (-0.87)  95/168 (-0.80)  97/157 (-0.84)  255/397 (-0.99)  130/249 (-0.76) | 378/648 (-0.89)  95/168 (-0.81)  96/157 (-0.87)  251/397 (-0.97)  125/249 (-0.75) | 368/648 (-0.89)  94/168 (-0.83)  97/157 (-0.90)  242/397 (-0.96)  123/249 (-0.68) | 355/648 (-0.90)  93/168 (-0.87)  97/157 (-0.82)  242/397 (-0.96)  119/249 (-0.74) | 355/648 (-0.90)  90/168 (-0.82)  96/157 (-0.83)  237/397 (-0.93)  116/249 (-0.76) | 351/648 (-0.91)  87/168 (-0.83)  90/157 (-0.84)  231/397 (-0.96)  113/249 (-0.73) |  |  |  |
| **hsCRP**  PBO to UPA  ADA continued  ADA to UPA  UPA continued  UPA to ADA | 380/650 (-13.8)  96/167 (-8.4)  98/159 (-18.1)  254/399 (-11.2)  129/252 (-14.9) | 381/650 (-13.1)  90/167 (-8.7)  96/159 (-18.6)  244/399 (-12.9)  120/252 (-14.1) | 361/650 (-12.9)  95/167 (-9.0)  97/159 (-17.6)  241/399 (-12.2)  127/252 (-11.0) | 356/650 (-12.8)  92/167 (-9.9)  94/159 (-17.6)  240/399 (-11.4)  118/252 (-13.2) | 337/650 (-13.8)  92/167 (-9.4)  94/159 (-15.7)  232/399 (-13.4)  116/252 (-11.5) | 333/650 (-13.5)  88/167 (-8.7)  90/159 (-14.8)  232/399 (-13.3)  112/252 (-14.7) | 321/650 (-12.9)  84/167 (-6.6)  90/159 (-17.3)  220/399 (-12.1)  110/252 (-14.5) | 319/650 (-12.0)  81/167 (-9.3)  89/159 (-16.3)  216/399 (-12.3)  108/252 (-15.4) |  |  |  |

ACR, American College of Rheumatology; ADA, adalimumab; AO, as observed; BL, baseline; HAQ-DI, Health Assessment Questionnaire-Disability Index; hsCRP, high-sensitivity C-reactive protein; NRS, numerical rating scale; PBO, placebo; PhGA, physician’s global assessment of disease activity; PtGA, patient’s global assessment of disease activity; SJC66, swollen joint count based on 66 joints; TJC68, tender joint count based on 68 joints; UPA, upadacitinib.

**Table S14**  Mean change from baseline in TJC68, SJC66, PtGA (0–100 mm VAS), PhGA (0–100 mm VAS), PtPain (0–100 mm VAS), HAQ-DI (0–3 scale) and hsCRP (mg/L) in patients who were non-responders or incomplete responders to the initial therapy and switched to the alternate therapy by week 26 (AO)

| **Weeks post-switch** | | | | | | | | | | | | | | | | | | | | |
| --- | --- | --- | --- | --- | --- | --- | --- | --- | --- | --- | --- | --- | --- | --- | --- | --- | --- | --- | --- | --- |
| **N (mean change)** | | **Switch** | | **12** | | **24** | | **36** | | **48** | | **60** | **72** | **84** | **96** | | **108** | | **120** | |
| **TJC68**  UPA to ADA – NR  UPA to ADA – IR  ADA to UPA – NR  ADA to UPA – IR | | 125 (-5.0)  126 (-21.0)  77 (-1.9)  82 (-18.9) | | 120 (-15.7)  124 (-23.1)  75 (-14.0)  79 (-23.2) | | 114 (-17.5)  123 (-23.8)  72 (-17.2)  77 (-24.3) | | 113 (-20.3)  115 (-24.9)  69 (-17.4)  73 (-25.5) | | 107 (-20.4)  104 (-26.1)  69 (-18.8)  71 (-26.2) | | 103 (-21.3)  103 (-26.3)  67 (-20.5)  68 (-26.1) | 99 (-22.9)  99 (-26.3)  66 (-20.2)  68 (-25.4) | 93 (-21.9)  95 (-26.6)  63 (-20.2)  66 (-27.0) | 91 (-23.1)  94 (-26.9)  62 (-20.4)  64 (-26.6) | | 91 (-22.5)  93 (-26.6)  62 (-20.6)  64 (-27.1) | | 90 (-21.9)  90 (-27.0)  61 (-21.0)  59 (-28.8) | |
| **SJC66**  UPA to ADA – NR  UPA to ADA – IR  ADA to UPA – NR  ADA to UPA – IR | | 125 (-2.1)  126 (-13.6)  77 (-3.8)  82 (-13.9) | | 120 (-9.5)  124 (-14.4)  75 (-10.8)  79 (-16.3) | | 114 (-11.7)  123 (-15.3)  72 (-11.2)  77 (-16.5) | | 113 (-11.9)  115 (-15.3)  69 (-12.3)  73 (-16.6) | | 107 (-12.0)  104 (-15.9)  69 (-12.5)  71 (-17.3) | | 103 (-12.1)  103 (-16.0)  67 (-13.1)  68 (-16.9) | 99 (-12.5)  99 (-16.3)  66 (-12.7)  68 (-16.8) | 93 (-12.7)  95 (-16.2)  63 (-13.5)  66 (-17.3) | 91 (-13.2)  94 (-16.1)  62 (-13.2)  64 (-18.0) | | 91 (-12.9)  93 (-16.0)  62 (-13.6)  64 (-17.8) | | 90 (-13.2)  90 (-16.4)  61 (-14.1)  59 (-18.4) | |
| **PtGA**  UPA to ADA – NR  UPA to ADA – IR  ADA to UPA – NR  ADA to UPA – IR | | 125 (-15.4)  124 (-27.0)  76 (-16.8)  81 (-25.3) | | 120 (-22.1)  122 (-29.6)  75 (-32.3)  78 (36.8) | | 114 (-24.5)  119 (-32.2)  72 (-34.6)  76 (-40.6) | | 113 (-28.5)  112 (-34.8)  69 (-35.7)  72 (-41.6) | | 107 (-27.9)  102 (-35.1)  68 (-35.5)  70 (-42.5) | | 103 (-28.9)  102 (-39.7)  67 (-37.2)  67 (-44.2) | 98 (-36.1)  98 (-40.8)  65 (-37.2)  67 (-45.1) | 93 (-30.3)  94 (-40.1)  62 (-42.4)  65 (-45.4) | 91 (-31.5)  93 (-41.0)  62 (-34.8)  63 (-47.0) | | 91 (-29.5)  92 (-39.9)  62 (-38.9)  63 (-46.4) | | 90 (-32.2)  89 (-40.0)  61 (-39.3)  58 (-47.4) | |
| **PhGA**  UPA to ADA – NR  UPA to ADA – IR  ADA to UPA – NR  ADA to UPA – IR | | 120 (-21.4)  120 (-40.4)  69 (-21.1)  74 (-39.9) | | 115 (-33.2)  116 (-41.7)  68 (-40.9)  68 (-46.9) | | 110 (-36.8)  114 (-43.5)  66 (-45.1)  68 (-52.2) | | 103 (-41.4)  106 (-48.5)  63 (-45.4)  65 (-54.0) | | 100 (-41.1)  98 (-50.3)  62 (-48.4)  63 (-56.3) | | 98 (-42.1)  98 (-52.2)  59 (-50.2)  59 (-57.3) | 93 (-45.7)  93 (-51.1)  59 (-49.8)  60 (-57.5) | 90 (-44.8)  90 (-52.2)  57 (-51.8)  57 (-56.8) | 85 (-44.9)  89 (-54.9)  55 (-51.6)  56 (-56.8) | | 83 (-46.5)  86 (-54.1)  56 (-50.6)  56 (-58.1) | | 87 (-45.1)  86 (-52.6)  56 (-50.5)  51 (-59.0) | |
| **PtPain**  UPA to ADA – NR  UPA to ADA – IR  ADA to UPA – NR  ADA to UPA – IR | | 125 (-17.5)  124 (-29.7)  76 (-18.3)  81 (-25.8) | | 120 (-25.3)  122 (-34.5)  75 (-32.9)  78 (-37.9) | | 114 (-26.5)  119 (-34.4)  72 (-34.6)  76 (-41.0) | | 113 (-31.6)  112 (-37.5)  69 (-36.1)  72 (-40.8) | | 107 (-30.3)  102 (-37.8)  68 (-36.3)  70 (-42.8) | | 103 (-28.5)  102 (-40.0)  67 (-39.1)  67 (-44.1) | 98 (-33.4)  98 (-42.1)  65 (-38.3)  67 (-44.4) | 93 (-32.6)  94 (-42.8)  63 (-44.7)  65 (-43.7) | 91 (-35.3)  93 (-44.3)  62 (-37.4)  63 (-44.6) | | 91 (-32.3)  92 (-42.0)  62 (-37.5)  63 (-45.2) | | 90 (-33.1)  89 (-43.8)  61 (-39.7)  58 (-44.2) | |
| **HAQ-DI**  UPA to ADA – NR  UPA to ADA – IR  ADA to UPA – NR  ADA to UPA – IR | | 125 (-0.38)  123 (-0.57)  76 (-0.40)  81 (-0.54) | | 120 (-0.49)  121 (-0.62)  75 (-0.63)  78 (-0.75) | | 114 (-0.52)  118 (-0.63)  72 (-0.67)  76 (-0.78) | | 113 (-0.55)  111 (-0.73)  69 (-0.74)  72 (-0.82) | | 107 (-0.61)  101 (-0.72)  68 (-0.79)  70 (-0.93) | | 103 (-0.58)  101 (-0.76)  67 (-0.75)  67 (-0.85) | 98 (-0.66)  97 (-0.78)  65 (-0.70)  67 (-0.91) | 93 (-0.64)  93 (-0.81)  63 (-0.78)  65 (-0.92) | 90 (-0.71)  92 (-0.79)  62 (-0.76)  63 (-0.92) | | 91 (-0.66)  91 (-0.78)  62 (-0.75)  63 (-0.92) | | 90 (-0.65)  88 (-0.78)  61 (-0.75)  58 (-1.00) | |
| **hsCRP**  UPA to ADA – NR  UPA to ADA – IR  ADA to UPA – NR  ADA to UPA – IR | | 121 (-11.6)  126 (-13.2)  74 (-9.7)  82 (-13.3) | | 119 (-11.2)  114 (-12.9)  74 (-13.6)  76 (-19.3) | | 109 (-10.8)  123 (-11.3)  70 (-13.4)  78 (-18.2) | | 113 (-9.3)  114 (-10.8)  68 (-16.5)  72 (-19.7) | | 104 (-7.7)  103 (-13.2)  68 (-14.7)  68 (-18.9) | | 103 (-8.6)  102 (-10.7)  66 (-15.1)  67 (-18.1) | 97 (-9.9)  97 (-10.6)  65 (-16.5)  67 (-14.8) | 90 (-10.3)  93 (-10.9)  62 (-17.1)  59 (-20.6) | 89 (-8.7)  91 (-10.3)  60 (-12.2)  64 (-19.5) | | 90 (-10.9)  89 (-13.8)  61 (-17.1)  60 (-19.6) | | 87 (-11.6)  81 (-12.3)  56 (-15.8)  56 (-21.0) | |
| **Weeks post-switch** | | | | | | | | | | | | | | | | | | |  |  |
| **N (mean change)** | | **132** | | **144** | | **156** | | **168** | | **180** | **192** | **204** | **216** | **228** | **240** | | **252** | |  |  |
| **TJC68**  UPA to ADA – NR  UPA to ADA – IR  ADA to UPA – NR  ADA to UPA – IR | | 86 (-23.3)  86 (-26.2)  58 (-20.8)  59 (-28.8) | | 76 (-23.7)  84 (-26.6)  58 (-21.5)  60 (-28.4) | | 79 (-23.0)  81 (-27.6)  53 (-23.1)  56 (-28.7) | | 76 (-23.1)  79 (-27.0)  55 (-21.5)  59 (-28.1) | | 76 (-24.9)  76 (-28.4)  53 (-22.1)  57 (-28.2) | 73 (-24.3)  72 (-28.4)  54 (-22.7)  54 (-29.1) | 73 (-25.2)  74 (-28.1)  55 (-23.0)  54 (-29.4) | 73 (-24.8)  75 (-27.4)  53 (-23.0)  51 (-30.0) | 74 (-25.4)  70 (-28.9)  52 (-22.3)  51 (-29.8) | 72 (-25.9)  72 (-28.9)  52 (-22.6)  49 (-29.7) | | 69 (-26.0)  72 (-28.5)  51 (-22.2)  50 (-29.4) | |  |  |
| **SJC66**  UPA to ADA – NR  UPA to ADA – IR  ADA to UPA – NR  ADA to UPA – IR | | 86 (-13.3)  86 (-15.8)  58 (-13.9)  59 (-18.6) | | 76 (-13.0)  84 (-16.2)  58 (-14.1)  60 (-18.3) | | 79 (-13.6)  81 (-16.7)  53 (-14.6)  56 (-18.1) | | 76 (-13.9)  79 (-16.2)  55 (-14.1)  59 (-17.6) | | 76 (-14.3)  79 (-16.5)  53 (-14.4)  57 (-18.2) | 73 (-14.1)  72 (-16.5)  54 (-15.1)  54 (-18.9) | 73 (-14.5)  74 (-16.8)  55 (-15.0)  54 (-18.5) | 73 (-14.5)  75 (-16.4)  53 (-14.3)  51 (-19.1) | 74 (-14.6)  70 (-17.0)  52 (-14.5)  51 (-19.4) | 72 (-14.9)  72 (-16.7)  52 (-14.5)  49 (-19.4) | | 69 (-15.4)  72 (-16.6)  51 (-14.5)  50 (-19.6) | |  |  |
| **PtGA**  UPA to ADA – NR  UPA to ADA – IR  ADA to UPA – NR  ADA to UPA – IR | | 85 (-32.6)  85 (-38.3)  58 (-42.3)  57 (-50.3) | | 77 (-34.6)  83 (-38.9)  57 (-42.6)  58 (-46.8) | | 79 (-34.6)  80 (-41.9)  53 (-41.8)  55 (-51.5) | | 76 (-32.4)  78 (-39.3)  55 (-43.1)  58 (-48.7) | | 76 (-33.7)  74 (-42.9)  53 (-37.2)  55 (-46.4) | 73 (-32.7)  72 (-41.2)  54 (-43.1)  52 (-49.5) | 73 (-33.8)  74 (-35.6)  55 (-42.4)  53 (-49.5) | 74 (-34.3)  75 (-38.0)  53 (-42.0)  50 (-51.5) | 73 (-38.1)  67 (-41.7)  52 (-39.2)  50 (-44.4) | 71 (-36.0)  71 (-39.9)  51 (-42.3)  48 (-48.1) | | 69 (-36.8)  72 (-39.2)  51 (-42.2)  49 (-48.8) | |  |  |
| **PhGA**  UPA to ADA – NR  UPA to ADA – IR  ADA to UPA – NR  ADA to UPA – IR | | 81 (-44.2)  82 (-54.5)  52 (-51.8)  50 (-60.0) | | 69 (-48.1)  79 (-54.6)  52 (-52.3)  53 (-59.3) | | 75 (-46.0)  73 (-57.8)  48 (-53.1)  50 (-59.7) | | 72 (-45.3)  73 (-54.5)  50 (-52.4)  49 (-57.8) | | 74 (-47.4)  71 (-56.3)  49 (-53.2)  49 (-57.1) | 71 (-46.4)  68 (-56.1)  50 (-55.4)  47 (-57.8) | 69 (-46.9)  70 (-55.2)  51 (-51.5)  47 (-58.9) | 69 (-47.3)  69 (-57.4)  48 (-51.9)  44 (-61.7) | 68 (-48.1)  63 (-57.9)  48 (-53.9)  45 (-59.3) | 68 (-52.2)  67 (-56.0)  47 (-54.0)  43 (-61.6) | | 65 (-52.1)  69 (-56.1)  47 (-52.3)  44 (-61.0) | |  |  |
| **PtPain**  UPA to ADA – NR  UPA to ADA – IR  ADA to UPA – NR  ADA to UPA – IR | | 85 (-34.0)  85 (-42.9)  58 (-41.3)  57 (-48.7) | | 77 (-36.6)  83 (-43.9)  57 (-41.9)  58 (-46.5) | | 79 (-36.7)  80 (-46.5)  53 (-41.8)  55 (-49.3) | | 76 (-32.5)  78 (-43.4)  55 (-42.4)  58 (-47.6) | | 76 (-35.8)  74 (-47.4)  53 (-38.8)  55 (-45.9) | 73 (-34.4)  72 (-46.2)  54 (-42.1)  52 (-46.6) | 73 (-36.0)  74 (-39.6)  55 (-42.9)  53 (-45.4) | 74 (-35.4)  75 (-44.7)  53 (-42.5)  50 (-47.6) | 73 (-36.4)  67 (-46.0)  52 (-37.8)  50 (-41.9) | 71 (-36.7)  71 (-43.2)  51 (-43.5)  48 (-45.7) | | 69 (-38.0)  72 (-43.3)  51 (-41.8)  49 (-46.4) | |  |  |
| **HAQ-DI**  UPA to ADA – NR  UPA to ADA – IR  ADA to UPA – NR  ADA to UPA – IR | | 85 (-0.63)  84 (-0.81)  58 (-0.81)  57 (-1.01) | | 77 (-0.69)  82 (-0.77)  57 (-0.84)  58 (-0.98) | | 79 (-0.66)  79 (-0.83)  53 (-0.85)  55 (-0.98) | | 76 (-0.69)  77 (-0.77)  55 (-0.86)  58 (-0.97) | | 76 (-0.69)  73 (-0.82)  53 (-0.83)  55 (-0.93) | 73 (-0.64)  71 (-0.81)  54 (-0.90)  52 (-0.90) | 73 (-0.71)  73 (-0.74)  55 (-0.84)  53 (-0.89) | 74 (-0.68)  74 (-0.83)  53 (-0.80)  50 (-0.92) | 73 (-0.69)  66 (-0.81)  52 (-0.73)  50 (-0.88) | 71 (-0.68)  69 (-0.85)  51 (-0.86)  48 (-0.98) | | 69 (-0.71)  71 (-0.83)  51 (-0.80)  49 (-0.93) | |  |  |
| **hsCRP**  UPA to ADA – NR  UPA to ADA – IR  ADA to UPA – NR  ADA to UPA – IR | | 81 (-12.1)  78 (-11.4)  52 (-16.8)  55 (-20.2) | | 74 (-12.9)  78 (-12.4)  52 (-16.2)  52 (-23.1) | | 71 (-7.9)  72 (-14.0)  46 (-18.2)  46 (-23.5) | | 72 (-12.9)  74 (-10.5)  51 (-15.7)  55 (-20.2) | | 71 (-12.7)  68 (-15.2)  49 (-13.7)  51 (-19.2) | 69 (-8.3)  69 (-15.4)  51 (-15.8)  51 (-17.6) | 69 (-12.3)  70 (-11.3)  50 (-16.9)  51 (-17.0) | 69 (-8.3)  69 (-15.5)  49 (-18.3)  48 (-16.6) | 71 (-10.3)  64 (-15.2)  50 (-15.2)  45 (-16.6) | 69 (-9.3)  70 (-16.1)  49 (-16.7)  45 (-19.1) | | 62 (-8.8)  65 (-17.4)  49 (-19.8)  42 (-18.6) | |  |  |

| **Weeks post-switch** | | | | | | | | | | | | |
| --- | --- | --- | --- | --- | --- | --- | --- | --- | --- | --- | --- | --- |
| **N (mean change)** | **264** | **276** | **288** | **300** | **312** | **324** | **336** |  |  |  |  |  |
| **TJC68**  UPA to ADA – NR  UPA to ADA – IR  ADA to UPA – NR  ADA to UPA – IR | 69 (-26.0)  69 (-28.7)  50 (-23.0)  50 (-29.2) | 69 (-25.3)  67 (-29.6)  49 (-22.4)  51 (-29.1) | 64 (-26.2)  68 (-29.5)  49 (-21.6)  51 (-29.0) | 65 (-25.8)  66 (-28.8)  48 (-22.1)  51 (-29.4) | 62 (-25.7)  65 (-28.9)  47 (-22.4)  51 (-29.5) | 61 (-25.2)  62 (-29.6)  47 (-23.1)  51 (-29.5) | 57 (-26.1)  63 (-29.2)  47 (-23.4)  51 (-29.4) |  |  |  |  |  |
| **SJC66**  UPA to ADA – NR  UPA to ADA – IR  ADA to UPA – NR  ADA to UPA – IR | 69 (-14.9)  69 (-16.4)  50 (-14.5)  50 (-19.1) | 69 (-15.1)  67 (-16.9)  49 (-14.8)  51 (-19.3) | 64 (-15.0)  68 (-16.9)  49 (-14.1)  51 (-19.4) | 65 (-15.2)  66 (-16.6)  48 (-14.5)  51 (-19.4) | 62 (-14.8)  65 (-16.1)  47 (-14.4)  51 (-19.5) | 61 (-14.8)  62 (-16.6)  47 (-14.6)  51 (-19.5) | 57 (-15.1)  63 (-16.8)  47 (-14.1)  51 (-19.4) |  |  |  |  |  |
| **PtGA**  UPA to ADA – NR  UPA to ADA – IR  ADA to UPA – NR  ADA to UPA – IR | 69 (-36.2)  69 (-38.4)  49 (-39.2)  49 (-48.6) | 69 (-35.6)  67 (-41.0)  49 (-35.7)  50 (-45.8) | 64 (-36.5)  67 (-40.0)  49 (-42.1)  50 (-46.0) | 64 (-33.7)  65 (-37.2)  46 (-40.8)  50 (-46.5) | 61 (-33.7)  64 (-37.5)  47 (-37.7)  50 (-44.2) | 60 (-32.9)  62 (-39.2)  47 (-41.1)  50 (-44.9) | 57 (-33.0)  61 (-38.3)  47 (-40.7)  50 (-43.5) |  |  |  |  |  |
| **PhGA**  UPA to ADA – NR  UPA to ADA – IR  ADA to UPA – NR  ADA to UPA – IR | 67 (-48.3)  63 (-56.9)  44 (-54.3)  42 (-58.7) | 64 (-47.3)  60 (-58.4)  44 (-49.8)  45 (-57.2) | 60 (-48.0)  64 (-56.6)  44 (-52.4)  44 (-56.2) | 61 (-51.1)  60 (-54.4)  44 (-54.0)  45 (-59.4) | 57 (-48.2)  61 (-57.6)  45 (-51.4)  43 (-59.9) | 58 (-45.7)  57 (-58.3)  45 (-52.0)  45 (-58.7) | 55 (-49.8)  58 (-58.3)  45 (-51.8)  45 (-58.5) |  |  |  |  |  |
| **PtPain**  UPA to ADA – NR  UPA to ADA – IR  ADA to UPA – NR  ADA to UPA – IR | 69 (-36.2)  69 (-44.7)  49 (-40.9)  49 (-46.7) | 69 (-35.9)  67 (-46.1)  49 (-37.5)  50 (-43.5) | 64 (-37.8)  67 (-44.6)  49 (-41.8)  50 (-42.8) | 64 (-35.2)  65 (-44.2)  46 (-43.0)  50 (-44.5) | 61 (-34.3)  64 (-44.0)  47 (-39.6)  50 (-44.9) | 60 (-34.1)  62 (-45.0)  47 (-43.7)  50 (-43.2) | 57 (-34.8)  61 (-44.3)  47 (-39.5)  50 (-43.3) |  |  |  |  |  |
| **HAQ-DI**  UPA to ADA – NR  UPA to ADA – IR  ADA to UPA – NR  ADA to UPA – IR | 69 (-0.68)  68 (-0.85)  49 (-0.78)  49 (-0.86) | 69 (-0.67)  66 (-0.87)  49 (-0.72)  50 (-0.86) | 64 (-0.67)  66 (-0.86)  49 (-0.77)  50 (-0.88) | 63 (-0.68)  64 (-0.83)  46 (-0.79)  50 (-0.91) | 61 (-0.64)  63 (-0.79)  47 (-0.83)  50 (-0.95) | 60 (-0.57)  61 (-0.81)  47 (-0.87)  50 (-0.89) | 57 (-0.69)  60 (-0.84)  47 (-0.77)  50 (-0.83) |  |  |  |  |  |
| **hsCRP**  UPA to ADA – NR  UPA to ADA – IR  ADA to UPA – NR  ADA to UPA – IR | 64 (-9.6)  64 (-17.9)  48 (-19.7)  49 (-19.4) | 64 (-13.3)  60 (-17.5)  48 (-16.3)  47 (-20.1) | 60 (-8.8)  64 (-18.4)  49 (-15.7)  49 (-19.3) | 62 (-4.6)  62 (-14.9)  48 (-17.3)  48 (-17.9) | 56 (-11.2)  60 (-16.6)  45 (-17.6)  49 (-14.0) | 54 (-5.5)  58 (-17.7)  45 (-17.0)  47 (-16.7) | 54 (-11.6)  59 (-18.8)  44 (-13.0)  45 (-16.9) |  |  |  |  |  |

ACR, American College of Rheumatology; ADA, adalimumab; AO, as observed; HAQ-DI, Health Assessment Questionnaire-Disability Index; hsCRP, high-sensitivity C-reactive protein; IR, incomplete responders; NR, non-responders; PhGA, physician’s global assessment of disease activity; PtGA, patient’s global assessment of disease activity; PtPain, patient’s assessment of pain; SJC66, swollen joint count based on 66 joints; TJC68, tender joint count based on 68 joints; UPA, upadacitinib; VAS, visual analogue scale.

**Table S15**  Cumulative steroid dose (mg) by analysis visit through week 372

| **Statistics** | **PBO to UPA (N=564)** | **ADA continued (N=127)** | **ADA to UPA**  **(N=141)** | **UPA continued (N=342)** | **UPA to ADA (N=228)** |
| --- | --- | --- | --- | --- | --- |
| **Week 2** | | | | | |
| N | 336 | 79 | 90 | 215 | 133 |
| Mean (SD) | 131.72 (54.205) | 133.18 (51.946) | 147.18 (64.510) | 129.56 (49.567) | 136.52 (50.078) |
| Median [95% CI] | 105.00 [105.00, 105.00] | 105.00 [105.00, 105.00] | 105.00 [105.00, 168.00] | 105.00 [105.00, 105.00] | 105.00 [105.00, 105.00] |
| **Week 4** | | | | | |
| N | 338 | 79 | 89 | 215 | 134 |
| Mean (SD) | 263.44 (111.530) | 265.88 (104.239) | 298.12 (128.826) | 258.75 (99.423) | 271.30 (101.801) |
| Median [95% CI] | 210.00 [210.00, 210.00] | 210.00 [210.00, 210.00] | 210.00 [210.00, 420.00] | 210.00 [210.00, 210.00] | 210.00 [210.00, 210.00] |
| **Week 8** | | | | | |
| N | 336 | 80 | 89 | 214 | 134 |
| Mean (SD) | 440.67 (182.092) | 438.22 (176.844) | 496.12 (213.519) | 431.20 (165.468) | 452.72 (169.103) |
| Median [95% CI] | 350.00 [350.00, 350.00] | 350.00 [350.00, 350.00] | 350.00 [350.00, 700.00] | 350.00 [350.00, 350.00] | 350.00 [350.00, 350.00] |
| **Week 12** | | | | | |
| N | 335 | 80 | 89 | 213 | 133 |
| Mean (SD) | 571.80 (236.038) | 571.58 (227.392) | 639.01 (285.069) | 559.13 (215.264) | 591.84 (217.112) |
| Median [95% CI] | 455.00 [455.00, 455.00] | 455.00 [455.00, 455.00] | 455.00 [455.00, 910.00] | 455.00 [455.00, 455.00] | 455.00 [455.00, 455.00] |
| **Week 14** | | | | | |
| N | 333 | 80 | 89 | 213 | 133 |
| Mean (SD) | 704.38 (288.583) | 704.04 (278.262) | 785.65 (350.644) | 687.53 (264.944) | 728.18 (267.393) |
| Median [95% CI] | 560.00 [560.00, 560.00] | 560.00 [560.00, 560.00] | 560.00 [560.00, 1120.00] | 560.00 [560.00, 560.00] | 560.00 [560.00, 560.00] |
| **Week 18** | | | | | |
| N | 335 | 80 | 88 | 212 | 133 |
| Mean (SD) | 874.04 (363.216) | 878.77 (347.018) | 990.85 (428.784) | 859.82 (330.801) | 907.69 (332.461) |
| Median [95% CI] | 700.00 [700.00, 700.00] | 700.00 [700.00, 717.50] | 700.00 [700.00, 1335.00] | 700.00 [700.00, 700.00] | 700.00 [700.00, 700.00] |
| **Week 22** | | | | | |
| Cumulative steroid dose (mg) |  |  |  |  |  |
| N | 333 | 80 | 88 | 212 | 133 |
| Mean (SD) | 1049.83 (432.610) | 1053.02 (415.431) | 1186.67 (512.484) | 1030.75 (396.433) | 1083.78 (409.631) |
| Median [95% CI] | 840.00 [840.00, 840.00] | 840.00 [840.00, 890.00] | 840.00 [840.00, 1475.00] | 840.00 [840.00, 840.00] | 840.00 [840.00, 880.00] |
| **Week 26** | | | | | |
| N | 335 | 80 | 88 | 212 | 133 |
| Mean (SD) | 1217.23 (510.606) | 1225.90 (483.185) | 1382.83 (596.934) | 1200.33 (461.737) | 1261.56 (475.619) |
| Median [95% CI] | 980.00 [980.00, 980.00] | 980.00 [980.00, 1030.00] | 980.00 [980.00, 1615.00] | 980.00 [980.00, 980.00] | 980.00 [980.00, 1030.00] |
| **Week 30** | | | | | |
| N | 334 | 81 | 88 | 213 | 135 |
| Mean (SD) | 1433.33 (599.247) | 1421.54 (587.661) | 1626.43 (701.131) | 1402.81 (547.411) | 1460.54 (576.437) |
| Median [95% CI] | 1155.00 [1155.00, 1155.00] | 1155.00 [1155.00, 1205.00] | 1155.00 [1155.00, 1848.00] | 1155.00 [1155.00, 1155.00] | 1155.00 [1155.00, 1205.00] |
| **Week 36** | | | | | |
| N | 333 | 78 | 88 | 207 | 132 |
| Mean (SD) | 1677.97 (717.839) | 1718.91 (661.230) | 1911.26 (835.206) | 1661.10 (648.901) | 1744.99 (662.321) |
| Median [95% CI] | 1365.00 [1365.00, 1365.00] | 1365.00 [1365.00, 1545.00] | 1410.00 [1365.00, 2184.00] | 1365.00 [1365.00, 1365.00] | 1365.00 [1365.00, 1415.00] |
| **Week 42** | | | | | |
| N | 328 | 78 | 89 | 206 | 131 |
| Mean (SD) | 1952.51 (809.488) | 1967.74 (770.349) | 2184.12 (964.402) | 1912.43 (745.241) | 2029.63 (786.168) |
| Median [95% CI] | 1575.00 [1575.00, 1575.00] | 1575.00 [1575.00, 1625.00] | 1575.00 [1575.00, 2500.00] | 1575.00 [1575.00, 1575.00] | 1575.00 [1575.00, 1690.00] |
| **Week 48** | | | | | |
| N | 328 | 77 | 88 | 204 | 132 |
| Mean (SD) | 2309.17 (985.216) | 2349.06 (932.690) | 2637.17 (1132.305) | 2273.32 (891.369) | 2402.17 (972.253) |
| Median [95% CI] | 1890.00 [1890.00, 1890.00] | 1890.00 [1890.00, 1940.00] | 2181.50 [1890.00, 3024.00] | 1890.00 [1890.00, 1890.00] | 1890.00 [1890.00, 2015.00] |
| **Week 60** | | | | | |
| N | 324 | 75 | 88 | 198 | 133 |
| Mean (SD) | 2800.59 (1200.082) | 2851.53 (1235.499) | 3178.58 (1384.233) | 2769.80 (1070.673) | 2882.44 (1206.603) |
| Median [95% CI] | 2310.00 [2310.00, 2310.00] | 2310.00 [2310.00, 2472.50] | 2570.17 [2310.00, 3465.00] | 2310.00 [2310.00, 2310.00] | 2310.00 [2310.00, 2662.50] |
| **Week 72** | | | | | |
| N | 316 | 72 | 86 | 193 | 124 |
| Mean (SD) | 3301.89 (1389.036) | 3379.31 (1390.772) | 3782.43 (1574.090) | 3234.50 (1271.805) | 3428.49 (1400.640) |
| Median [95% CI] | 2730.00 [2730.00, 2730.00] | 2730.00 [2730.00, 2780.00] | 3238.00 [2730.00, 4110.00] | 2730.00 [2730.00, 2730.00] | 2730.00 [2730.00, 3047.50] |
| **Week 84** | | | | | |
| N | 304 | 72 | 83 | 182 | 119 |
| Mean (SD) | 3809.98 (1586.559) | 3844.54 (1676.711) | 4371.92 (1753.562) | 3706.64 (1482.193) | 4005.40 (1563.435) |
| Median [95% CI] | 3150.00 [3150.00, 3150.00] | 3150.00 [3150.00, 3200.00] | 3620.00 [3150.00, 4725.00] | 3150.00 [3150.00, 3150.00] | 3150.00 [3150.00, 3690.00] |
| **Week 96** | | | | | |
| N | 291 | 68 | 80 | 176 | 116 |
| Mean (SD) | 4303.63 (1798.654) | 4427.87 (1922.434) | 4925.65 (1989.964) | 4216.13 (1627.642) | 4357.21 (1842.191) |
| Median [95% CI] | 3570.00 [3570.00, 3570.00] | 3570.00 [3570.00, 3620.00] | 3855.00 [3570.00, 5355.00] | 3570.00 [3570.00, 3570.00] | 3570.00 [3570.00, 3870.00] |
| **Week 108** | | | | | |
| N | 286 | 66 | 81 | 171 | 110 |
| Mean (SD) | 4796.53 (2038.300) | 5053.72 (2077.382) | 5379.57 (2310.006) | 4694.27 (1818.419) | 4895.56 (1958.445) |
| Median [95% CI] | 3990.00 [3990.00, 3990.00] | 3990.00 [3990.00, 4377.50] | 4263.33 [3990.00, 5985.00] | 3990.00 [3990.00, 3990.00] | 3990.00 [3990.00, 4200.00] |
| **Week 120** | | | | | |
| N | 278 | 66 | 75 | 171 | 108 |
| Mean (SD) | 5251.26 (2338.902) | 5578.63 (2320.446) | 6046.06 (2462.334) | 5103.43 (2006.482) | 5432.89 (2071.937) |
| Median [95% CI] | 4410.00 [4410.00, 4410.00] | 4410.00 [4410.00, 4876.00] | 4880.00 [4410.00, 6615.00] | 4410.00 [4410.00, 4410.00] | 4410.00 [4410.00, 4961.00] |
| **Week 132** | | | | | |
| N | 267 | 64 | 76 | 161 | 105 |
| Mean (SD) | 5843.13 (2523.790) | 6127.29 (2636.577) | 6529.94 (2771.374) | 5587.42 (2185.943) | 5951.71 (2205.068) |
| Median [95% CI] | 4830.00 [4830.00, 4830.00] | 4830.00 [4830.00, 5355.00] | 5115.00 [4830.00, 7245.00] | 4830.00 [4830.00, 4830.00] | 4830.00 [4830.00, 6305.00] |
| **Week 144** | | | | | |
| N | 253 | 62 | 74 | 161 | 101 |
| Mean (SD) | 6422.01 (2748.480) | 6531.28 (2658.594) | 7150.97 (2941.326) | 6019.29 (2400.471) | 6510.32 (2357.466) |
| Median [95% CI] | 5250.00 [5250.00, 5250.00] | 5250.00 [5250.00, 5812.50] | 5535.00 [5250.00, 7875.00] | 5250.00 [5250.00, 5250.00] | 5250.00 [5250.00, 6445.00] |
| **Week 156** | | | | | |
| N | 241 | 61 | 72 | 156 | 97 |
| Mean (SD) | 6945.59 (3072.174) | 6924.29 (2893.771) | 7743.67 (3248.969) | 6490.07 (2585.116) | 6964.59 (2643.074) |
| Median [95% CI] | 5670.00 [5670.00, 5670.00] | 5670.00 [5670.00, 5887.50] | 6472.00 [5670.00, 8505.00] | 5670.00 [5670.00, 5670.00] | 5670.00 [5670.00, 7160.00] |
| **Week 168** | | | | | |
| N | 239 | 58 | 71 | 157 | 90 |
| Mean (SD) | 7401.05 (3352.266) | 7636.49 (2906.378) | 8339.59 (3485.818) | 6770.26 (2912.028) | 7328.17 (2836.075) |
| Median [95% CI] | 6090.00 [6090.00, 6090.00] | 6090.00 [6090.00, 6606.67] | 7308.00 [6090.00, 9135.00] | 6090.00 [6090.00, 6090.00] | 6090.00 [6090.00, 6525.00] |
| **Week 180** | | | | | |
| N | 230 | 57 | 69 | 151 | 90 |
| Mean (SD) | 7907.59 (3346.634) | 8046.35 (3020.240) | 9084.75 (3608.702) | 7370.50 (3012.492) | 7767.62 (3099.990) |
| Median [95% CI] | 6510.00 [6510.00, 6510.00] | 6510.00 [6510.00, 7122.50] | 7945.00 [6510.00, 9765.00] | 6510.00 [6510.00, 6510.00] | 6510.00 [6510.00, 7365.00] |
| **Week 192** | | | | | |
| N | 224 | 56 | 70 | 152 | 89 |
| Mean (SD) | 8404.70 (3558.968) | 8615.60 (3106.800) | 9517.18 (3973.493) | 7692.18 (3336.581) | 8144.51 (3515.580) |
| Median [95% CI] | 6930.00 [6930.00, 6930.00] | 6930.00 [6930.00, 7496.67] | 8340.50 [6930.00, 10395.00] | 6930.00 [6930.00, 6930.00] | 6930.00 [6930.00, 8205.00] |
| **Week 204** | | | | | |
| N | 221 | 55 | 70 | 150 | 85 |
| Mean (SD) | 8861.42 (3849.290) | 8777.50 (3456.435) | 9945.05 (4342.695) | 8122.78 (3450.175) | 9007.27 (3467.658) |
| Median [95% CI] | 7350.00 [7350.00, 7350.00] | 7350.00 [7350.00, 7430.00] | 8457.50 [7350.00, 11025.00] | 7350.00 [7350.00, 7350.00] | 7350.00 [7350.00, 9328.33] |
| **Week 216** | | | | | |
| N | 215 | 52 | 65 | 141 | 81 |
| Mean (SD) | 9356.84 (4173.170) | 9449.78 (3472.042) | 10776.97 (4346.651) | 8677.22 (3698.183) | 9731.03 (3578.123) |
| Median [95% CI] | 7770.00 [7770.00, 7770.00] | 7770.00 [7770.00, 8336.67] | 9324.00 [7770.00, 11655.00] | 7770.00 [7770.00, 7770.00] | 7770.00 [7770.00, 10849.00] |
| **Week 228** | | | | | |
| N | 210 | 47 | 63 | 140 | 76 |
| Mean (SD) | 9845.17 (4402.804) | 10047.24 (3609.735) | 11242.18 (4623.329) | 9012.17 (3895.247) | 10308.87 (3835.840) |
| Median [95% CI] | 8190.00 [8190.00, 8190.00] | 8190.00 [8190.00, 9667.50] | 9625.00 [8190.00, 12285.00] | 8190.00 [8190.00, 8190.00] | 8215.00 [8190.00, 11865.00] |
| **Week 240** | | | | | |
| N | 206 | 46 | 62 | 136 | 75 |
| Mean (SD) | 10341.65 (4489.875) | 10378.65 (3932.203) | 11820.35 (4962.799) | 9607.65 (3979.788) | 10909.82 (3953.901) |
| Median [95% CI] | 8610.00 [8610.00, 8610.00] | 8610.00 [8610.00, 10297.50] | 10088.13 [8610.00, 12915.00] | 8610.00 [8610.00, 8610.00] | 8660.00 [8610.00, 12075.00] |
| **Week 252** | | | | | |
| N | 205 | 44 | 60 | 132 | 74 |
| Mean (SD) | 10685.41 (4730.942) | 10997.69 (3994.048) | 12608.03 (5022.543) | 9854.20 (4186.181) | 11388.75 (4160.295) |
| Median [95% CI] | 9030.00 [9030.00, 9030.00] | 9030.00 [9030.00, 9596.67] | 10641.88 [9030.00, 13545.00] | 9030.00 [9030.00, 9030.00] | 9055.00 [9030.00, 12285.00] |
| **Week 264** | | | | | |
| N | 201 | 43 | 58 | 129 | 72 |
| Mean (SD) | 11231.13 (4864.540) | 11098.98 (4232.975) | 13151.40 (5304.167) | 10163.41 (4375.925) | 11607.40 (4608.325) |
| Median [95% CI] | 9450.00 [9450.00, 9450.00] | 9450.00 [9450.00, 10016.67] | 11018.75 [9450.00, 13900.00] | 9450.00 [9450.00, 9450.00] | 9450.00 [9450.00, 12529.00] |
| **Week 276** | | | | | |
| N | 194 | 44 | 60 | 122 | 69 |
| Mean (SD) | 11733.18 (4902.900) | 11522.93 (4480.465) | 13386.91 (5751.667) | 10734.27 (4600.358) | 12392.70 (4673.385) |
| Median [95% CI] | 9870.00 [9870.00, 9870.00] | 9870.00 [9870.00, 10436.67] | 10637.50 [9870.00, 14320.00] | 9870.00 [9870.00, 9870.00] | 9870.00 [9870.00, 14085.00] |
| **Week 288** | | | | | |
| N | 194 | 43 | 55 | 124 | 72 |
| Mean (SD) | 12018.94 (5270.168) | 12259.23 (4323.854) | 14375.11 (5894.721) | 11038.15 (4909.082) | 12458.13 (5284.242) |
| Median [95% CI] | 10290.00 [10290.00, 10290.00] | 10290.00 [10290.00, 12010.00] | 11725.00 [10290.00, 15435.00] | 10290.00 [10290.00, 10290.00] | 10290.00 [10290.00, 13369.00] |
| **Week 300** | | | | | |
| N | 193 | 42 | 54 | 121 | 67 |
| Mean (SD) | 12516.11 (5388.585) | 12716.93 (4516.356) | 14822.22 (6139.347) | 11660.14 (4962.416) | 13381.47 (5052.034) |
| Median [95% CI] | 10710.00 [10710.00, 10710.00] | 10710.00 [10710.00, 11276.67] | 11536.25 [10710.00, 16065.00] | 10710.00 [10710.00, 10710.00] | 10710.00 [10710.00, 13860.00] |
| **Week 312** | | | | | |
| N | 186 | 40 | 54 | 118 | 66 |
| Mean (SD) | 12917.22 (5696.802) | 13265.93 (4763.867) | 15233.13 (6377.585) | 12203.94 (5146.455) | 13863.54 (5182.260) |
| Median [95% CI] | 11130.00 [11130.00, 11130.00] | 11130.00 [11130.00, 11696.67] | 11577.50 [11130.00, 15580.00] | 11130.00 [11130.00, 11130.00] | 11155.00 [11130.00, 15330.00] |
| **Week 324** | | | | | |
| N | 180 | 43 | 55 | 114 | 65 |
| Mean (SD) | 13445.88 (5667.980) | 13072.80 (5433.409) | 15602.73 (6891.947) | 12585.09 (5473.203) | 14483.67 (5427.278) |
| Median [95% CI] | 11550.00 [11550.00, 11550.00] | 11550.00 [11550.00, 11567.50] | 11842.50 [11550.00, 17325.00] | 11550.00 [11550.00, 11550.00] | 11550.00 [11550.00, 17325.00] |
| **Week 336** | | | | | |
| N | 184 | 39 | 55 | 113 | 63 |
| Mean (SD) | 13556.05 (6168.740) | 13303.95 (5621.483) | 18957.45 (20638.990) | 12724.65 (5832.586) | 15110.43 (5676.158) |
| Median [95% CI] | 11970.00 [11970.00, 11970.00] | 11970.00 [11970.00, 11987.50] | 13210.00 [11970.00, 17955.00] | 11970.00 [11970.00, 11970.00] | 12270.00 [11970.00, 17955.00] |
| **Week 348** | | | | | |
| N | 181 | 39 | 53 | 109 | 62 |
| Mean (SD) | 13992.01 (6484.625) | 13923.80 (5448.510) | 16741.76 (7494.928) | 13317.34 (5778.365) | 15301.77 (6120.683) |
| Median [95% CI] | 12390.00 [12390.00, 12390.00] | 12390.00 [12390.00, 12407.50] | 12490.00 [12390.00, 18585.00] | 12390.00 [12390.00, 12390.00] | 12540.00 [12390.00, 18585.00] |
| **Week 360** | | | | | |
| N | 176 | 36 | 53 | 103 | 58 |
| Mean (SD) | 14703.23 (6450.338) | 14663.53 (5679.067) | 20064.95 (21118.630) | 13998.38 (5939.744) | 16151.47 (5958.662) |
| Median [95% CI] | 12810.00 [12810.00, 12810.00] | 12810.00 [12810.00, 12827.50] | 13027.50 [12810.00, 19215.00] | 12810.00 [12810.00, 12810.00] | 12960.00 [12810.00, 19215.00] |
| **Week 372** | | | | | |
| N | 170 | 34 | 48 | 100 | 58 |
| Mean (SD) | 15476.49 (6439.799) | 15241.33 (5975.914) | 18398.70 (7677.785) | 14588.33 (6124.062) | 16527.15 (6377.492) |
| Median [95% CI] | 13230.00 [13230.00, 13230.00] | 13230.00 [13187.50, 13951.67] | 14661.75 [13230.00, 19845.00] | 13230.00 [13230.00, 13230.00] | 13680.00 [13230.00, 19733.33] |

ADA, adalimumab; CI, confidence interval; PBO, placebo; SD, standard deviation; UPA, upadacitinib.

**Figure S1**  Proportions of patients achieving CDAI remission (≤2.8), CDAI LDA (≤10), DAS28(CRP) ≤2.6 and DAS28(CRP) ≤3.2 through 372 weeks (NRI).


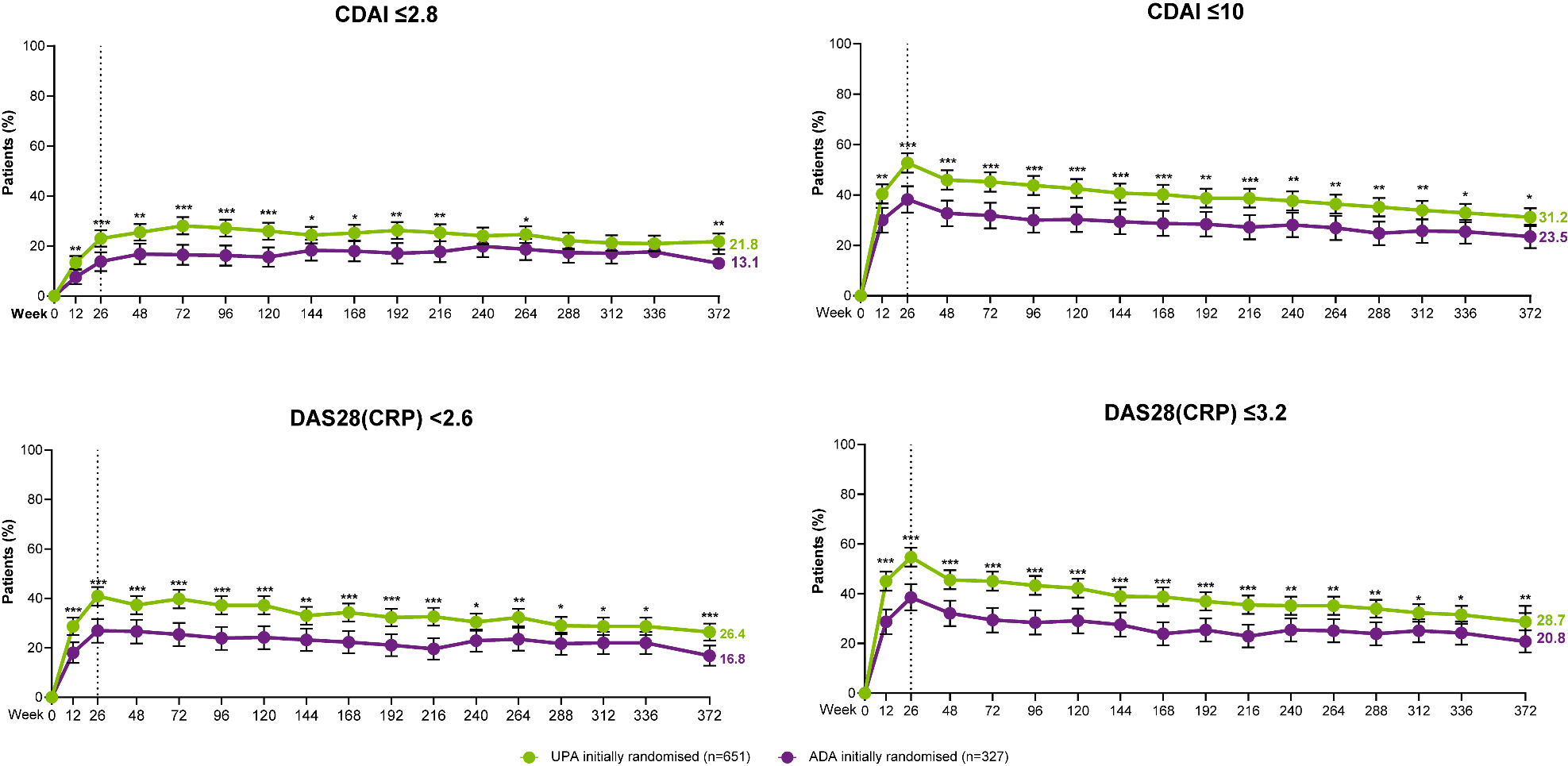


*p<0.05, **p<0.01, ***p<0.001 for UPA 15 mg once daily versus ADA 40 mg every other week.

Treatment groups are by initial randomisation. NRI was used for patients who were rescued or prematurely discontinued study drug, as well as for missing data. Data points plotted here are shown in supplemental table S11.

ADA, adalimumab; CDAI, Clinical Disease Activity Index; DAS28(CRP), 28-joint Disease Activity Score based on C-reactive protein; LDA, low disease activity; NRI, non-responder imputation; UPA, upadacitinib.


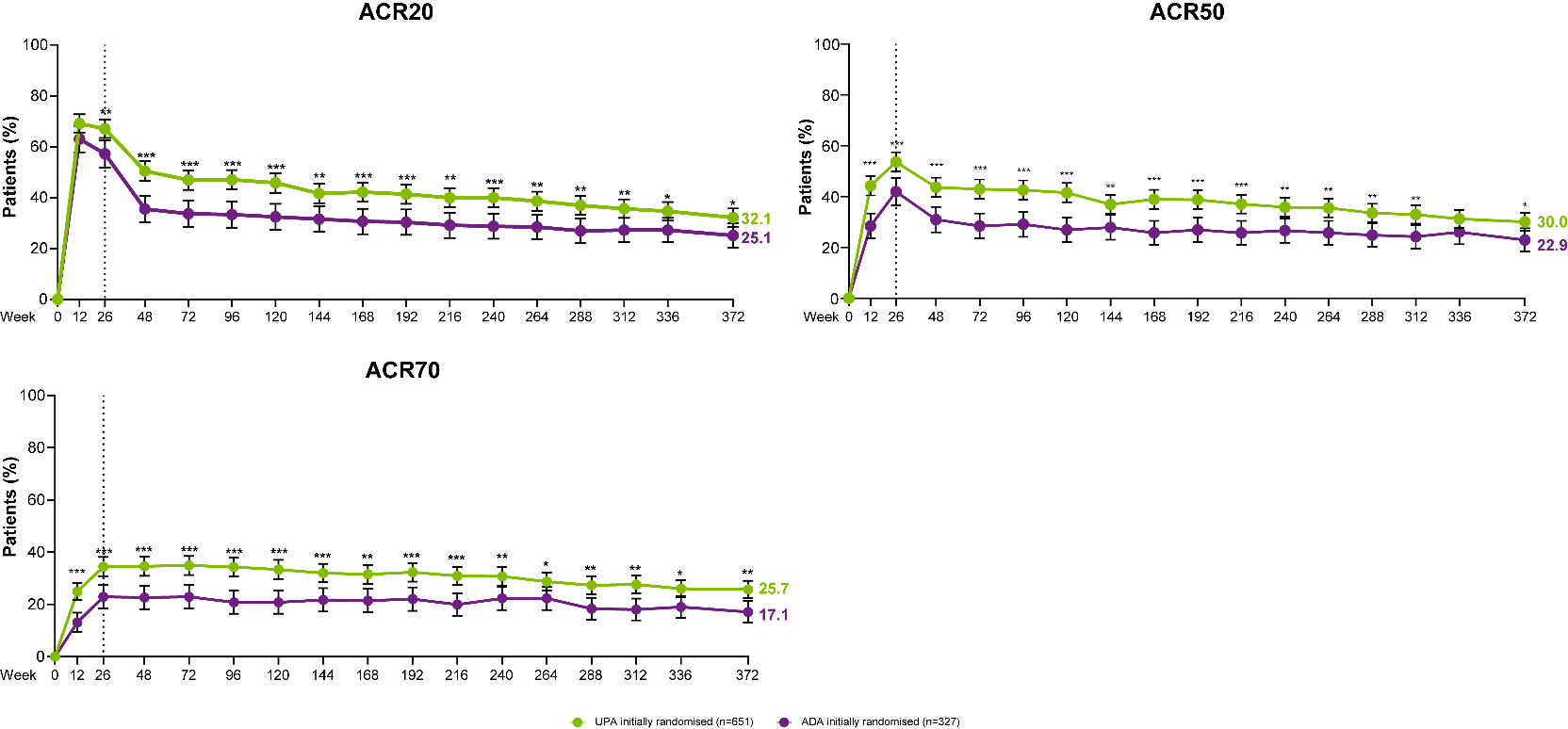
**Figure S2** Proportions of patients achieving ACR20, ACR50 and ACR70 through 372 weeks (NRI).

*p<0.05, **p<0.01, ***p<0.001 for UPA 15 mg once daily versus ADA 40 mg every other week.

Treatment groups are by initial randomisation. NRI was used for patients who were rescued or prematurely discontinued study drug, as well as for missing data.

Data points plotted here are shown in supplemental table S12.

ACR20/50/70, ≥20/50/70% improvement in American College of Rheumatology response criteria; ADA, adalimumab; NRI, non-responder imputation; UPA, upadacitinib.

**Figure S3** Mean change from baseline in core components of ACR criteria through 372 weeks (AO)

**
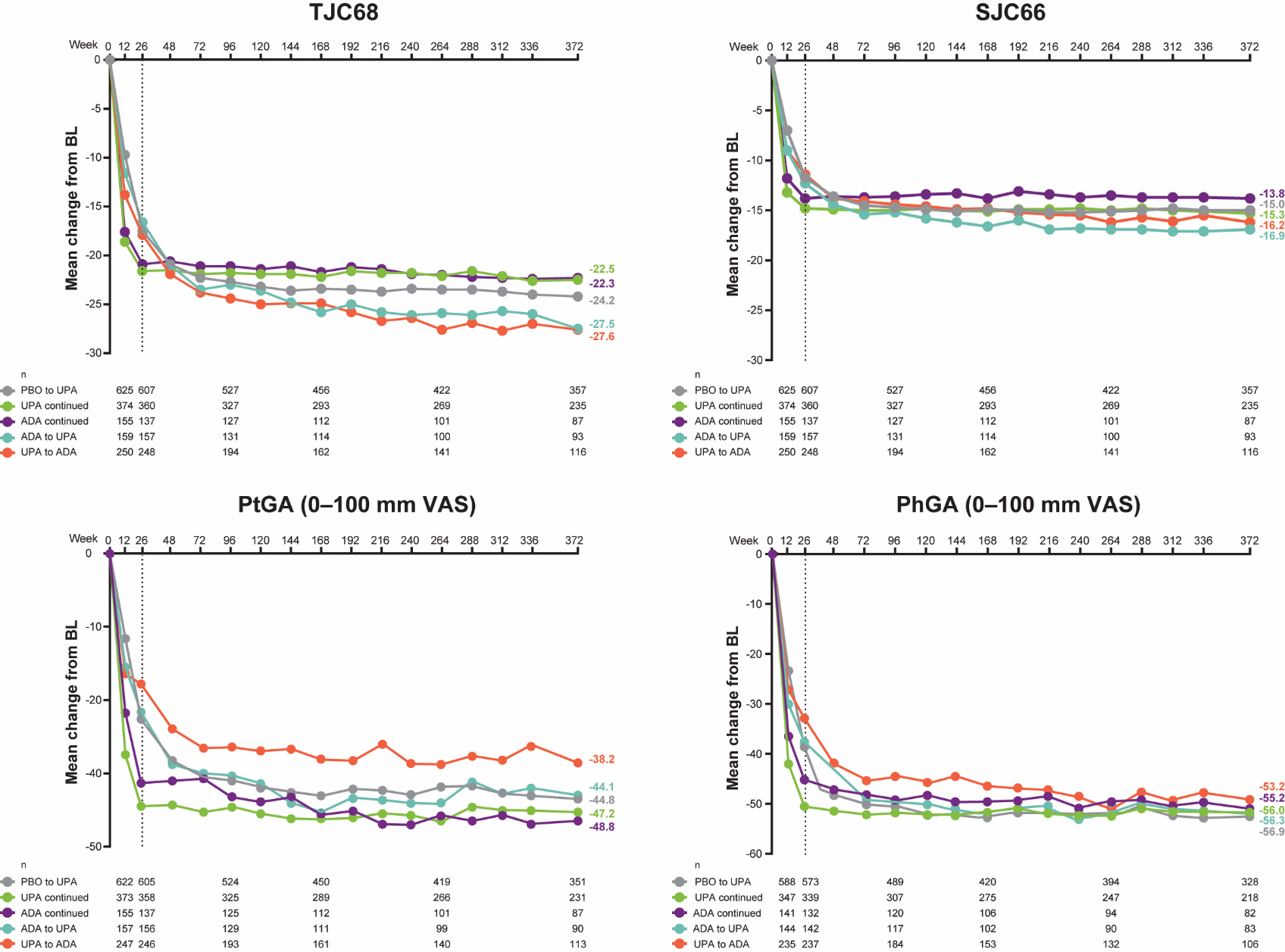
**

**
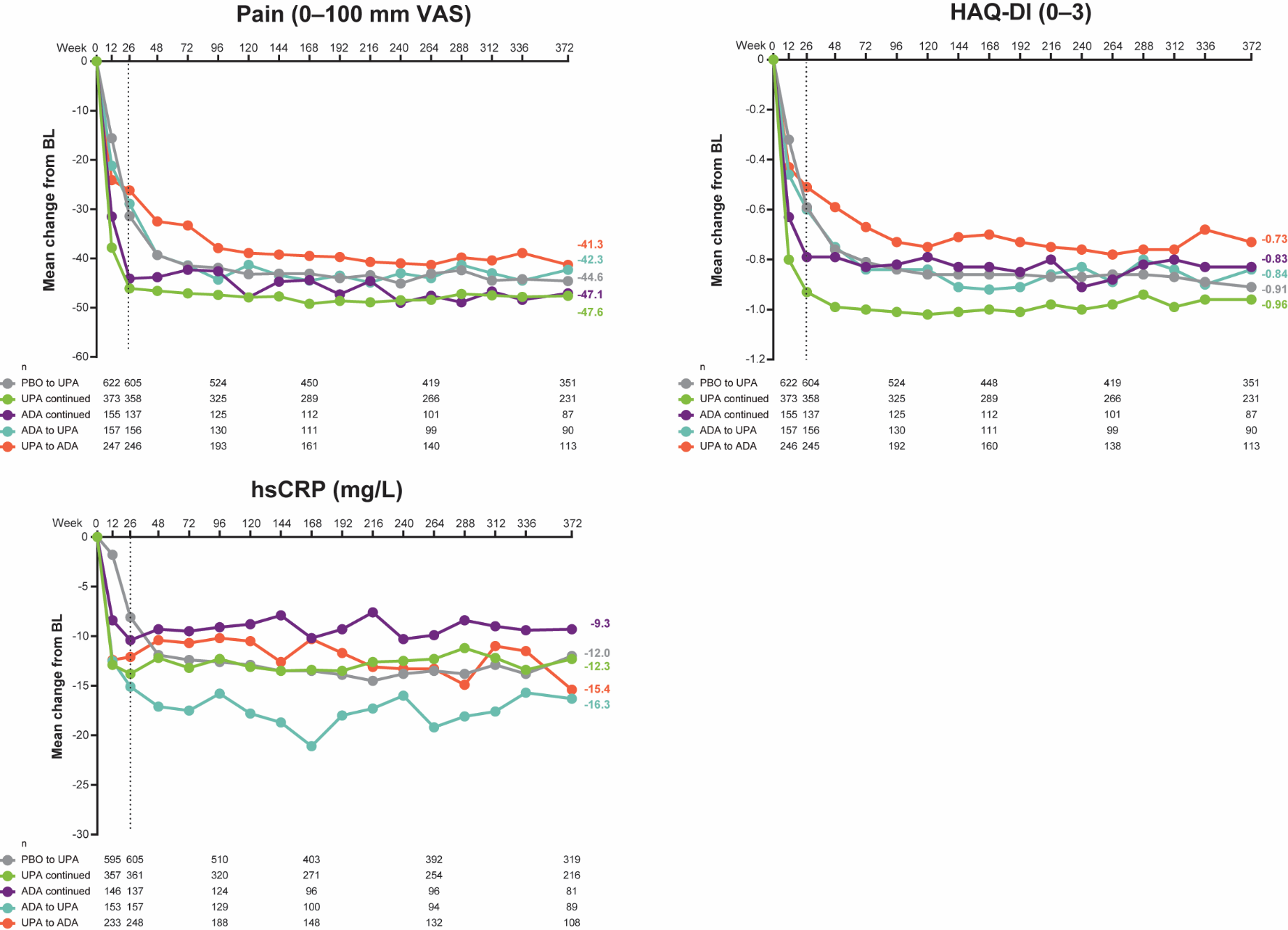
**

Groups are by treatment sequence AO, without imputation for missing data. All patients in the PBO group who were not previously rescued were switched to UPA at week 26.

Sample sizes (n) are shown below each graph. Data points plotted here are shown in supplemental table S13.

ACR, American College of Rheumatology; ADA, adalimumab; AO, as observed; BL, baseline; HAQ-DI, Health Assessment Questionnaire Disability Index; hsCRP, high-sensitivity C-reactive protein; PBO, placebo; PhGA, physician’s global assessment of disease activity; PtGA, patient’s global assessment of disease activity; PtPain, patient’s assessment of pain; SJC66, swollen joint count of 66 joints; TJC68, tender joint count of 68 joints; UPA, upadacitinib; VAS, visual analogue scale.

**Figure S4** Mean change from baseline for the following endpoints: TJC68, SJC66, PtGA (0–100 mm VAS), PhGA (0–100 mm VAS), PtPain (0–100 mm VAS), HAQ-DI (0–3 scale) and hsCRP (mg/L) in patients who were non-responders or incomplete responders to the initial therapy and switched to the alternate therapy by week 26 (AO).


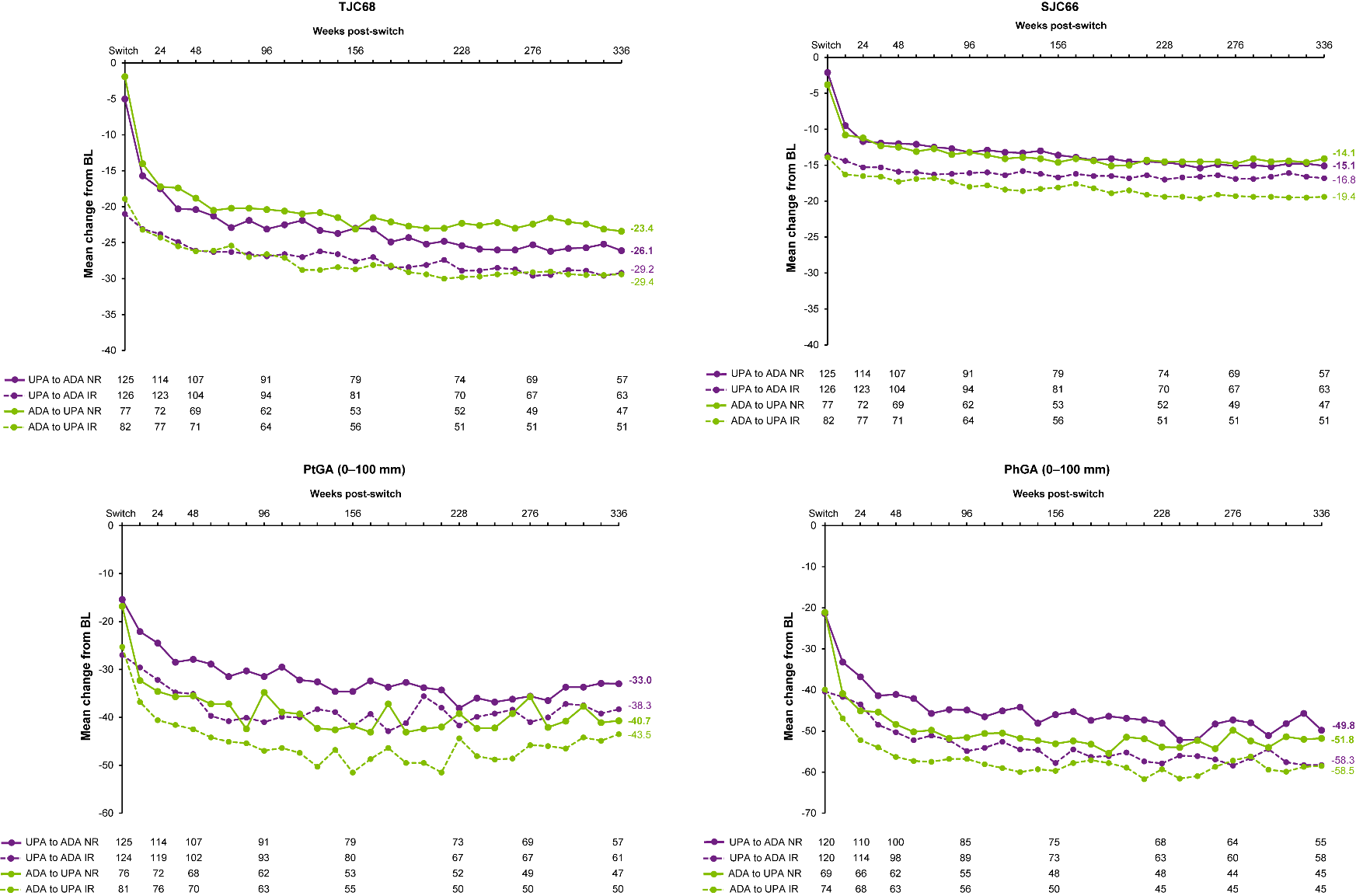


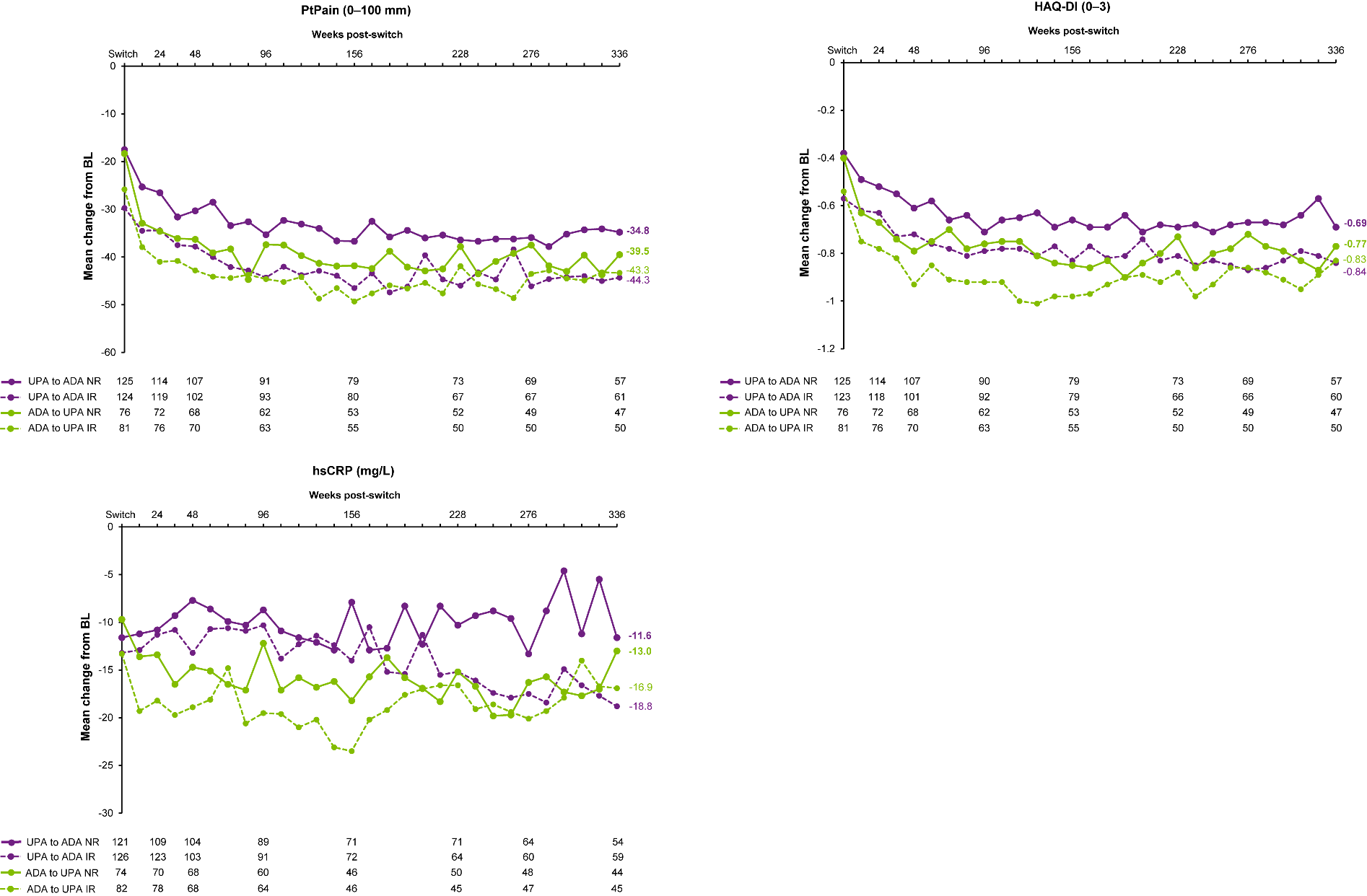


Groups are by treatment sequence AO, without imputation for missing data.

Sample sizes (n) are shown below each graph. Data points plotted here are shown in supplemental table S14.

ACR, American College of Rheumatology; ADA, adalimumab; AO, as observed; HAQ-DI, Health Assessment Questionnaire-Disability Index; hsCRP, high-sensitivity C-reactive protein; IR, incomplete responders; NR, non-responders; PhGA, physician’s global assessment of disease activity; PtGA, patient’s global assessment of disease activity; PtPain, patient’s assessment of pain; SJC66, swollen joint count based on 66 joints; TJC68, tender joint count based on 68 joints; UPA, upadacitinib; VAS, visual analogue scale.

**Figure S5** Cumulative steroid dose by analysis visit through 372 weeks in patients who entered the long-term extension period on study drug (AO).


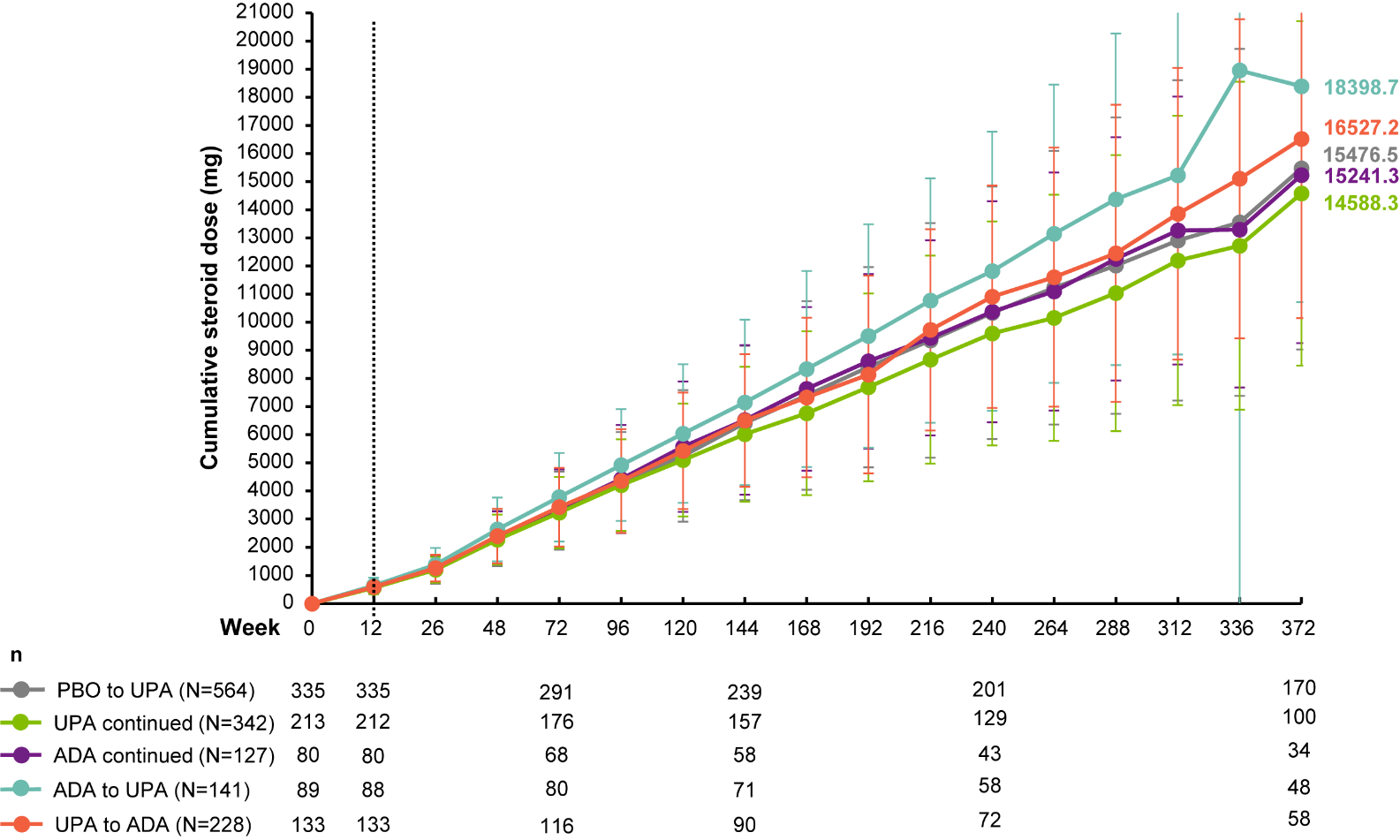


Values are shown as mean with standard deviation. Groups are shown by treatment sequence as observed, without imputation for missing data. All patients in the PBO group who were not previously rescued were switched to UPA at week 26.

Sample sizes (n) are shown below each graph. Data points plotted here are shown in supplemental table S15.

ADA, adalimumab; AO, as observed; PBO, placebo; UPA, upadacitinib.
